# ﻿A new classification system and taxonomic synopsis for Malpighiaceae (Malpighiales, Rosids) based on molecular phylogenetics, morphology, palynology, and chemistry

**DOI:** 10.3897/phytokeys.242.117469

**Published:** 2024-05-22

**Authors:** Rafael F. de Almeida, Isa L. de Morais, Thais Alves-Silva, Higor Antonio-Domingues, Marco O. O. Pellegrini

**Affiliations:** 1 Universidade Estadual de Goiás, Campus Sudoeste, Quirinópolis, Goiás, Brazil Universidade Estadual de Goiás Quirinópolis Brazil; 2 Royal Botanical Gardens, Kew, Richmond, UK Royal Botanical Gardens Richmond United Kingdom

**Keywords:** Conservation, Elatinaceae, integrated monography, phylogeny, systematics, taxonomy

## Abstract

Malpighiaceae has undergone unprecedented changes in its traditional classification in the past two decades due to several phylogenetic studies shedding light on the non-monophyly of all subfamilies and most tribes and genera. Even though morphological characters were used to reconstruct the last molecular generic phylogeny of Malpighiaceae, a new classification system has never been proposed for this family. Based on a comprehensive review of the last twenty years of published studies for this family, we propose a new classification system and provide a taxonomic synopsis for Malpighiaceae based on molecular phylogenetics, morphology, palynology, and chemistry as a baseline for the systematics, conservation, and taxonomy of this family worldwide. Malpighiaceae currently comprises two subfamilies (Byrsonimoideae and Malpighioideae), 12 tribes ( Acmanthereae, Acridocarpeae**trib. nov.**, Barnebyeae**trib. nov.**, Bunchosieae**trib. nov.**, Byrsonimeae, Galphimieae, Gaudichaudieae, Hiptageae, Hiraeeae, Malpighieae, Mcvaughieae**trib. nov.**, and Ptilochaeteae**trib. nov.**), 72 genera (incl. *Mamedea***gen. nov.**), and 1,499 accepted species (715 of which are currently under some kind of extinction threat). We present identification keys for all subfamilies, tribes, and genera, a full morphological description for the proposed new genus, the re-circumscription of ten genera alongside the needed new combinations, the proposition of several new synonyms, the typification of several names, and notes on the taxonomy, distribution, conservation, and ecology up to the genus rank. Morphological plates are also provided to illustrate the immense diversity of morphological traits used in the new classification and synopsis.

## ﻿Introduction

Malpighiaceae (Malpighiales) is a family of flowering plants currently comprising 75 genera and ca. 1,350 species of trees, shrubs, subshrubs, and lianas distributed in tropical and subtropical regions of the world (Almeida et al. 2020, 2023a; POWO 2024). This family includes several economically important species in the Neotropics, such as the Barbados cherry (*Malpighia* ssp.), murici or nanche berry (*Byrsonima* ssp.), and the Ayahuasca hallucinogenic tea [*Banisteriopsiscaapi* (Spruce ex Griseb.) C.V.Morton] (Almeida et al. 2020). Neotropical Malpighiaceae show a conspicuous floral conservatism characterised by monosymmetric (i.e., zygomorphic) and bisexual flowers with five sepals adnate at the base and abaxially (to the flower axis) bearing a pair of oil-secreting glands (i.e., elaiophores) near the base (sometimes absent from the anterior sepal or completely absent in few genera). This conspicuous floral conservatism of Neotropical Malpighiaceae was lost in the Old World genera due to a shift in their pollination system as an evolutionary adaptation to the absence of oil-collecting bees in the Paleotropics (Cameron et al. 2001; Davis et al. 2014; Almeida et al. 2023a). Consequently, Old World genera frequently show actinomorphic flowers with eglandular or nectariferous sepals and pantoporate pollen grains (Cameron et al. 2001; Davis et al. 2014).

**Figure 1.**
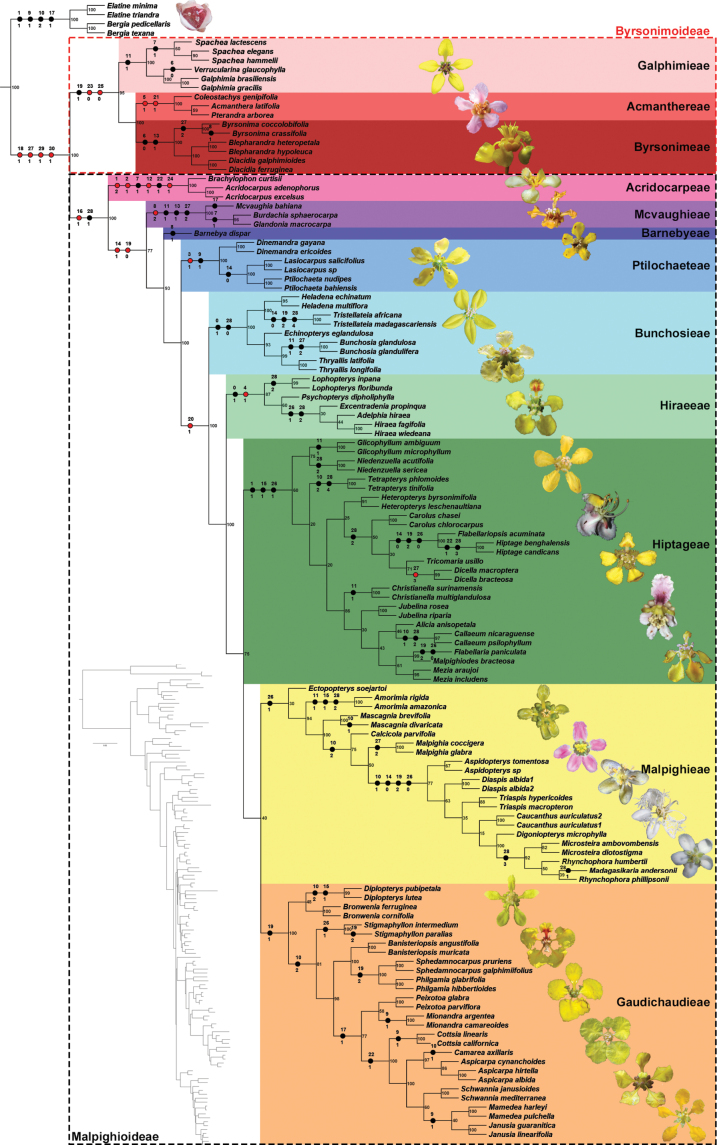
Molecular phylogenetic tree recovered by the ML of the reduced alignment for *matK*, *ndhF*, *rbcL*, and PHYC presented by Davis and Anderson (2010) with the taxonomic sampling reduced to one or three terminals according to the accepted genera in the present study. Left tree shows branch lengths recovered. Right tree shows the names of families, subfamilies, and tribes coloured according to the current classification recognised in this study. Bootstrap support values from the ML presented on the nodes. Black circles represent homoplasies, and red circles represent synapomorphies/autapomorphies recovered for each lineage. Numbers above circles represent character numbers from Suppl. material 2, while the numbers below the circles represent character states from the same file. Galphimieae: flower of *Galphimiagracilis* Bartl. by M.O.O. Pellegrini. Acmanthereae: flower of *Pterandrapyroidea* L. by R.F. Almeida. Byrsonimeae: flower of *Byrsonimasericea* DC. by R.F. Almeida. Acridocarpeae: flower of *Acridocarpuslongifolius* (G.Don) Hook.f. by E. Bidault. Mcvaughieae: flower of *Mcvaughiasergipana* Amorim & R.F.Almeida by R.F. Almeida. Barnebyeae: flower of *Barnebyaharleyi* W.R.Anderson & B.Gates by F. Flores. Ptilochaeteae: flower of *Ptilochaetabahiensis* Turcz. by R.F. Almeida. Bunchosieae: flowers of *Tristellateiamadagascariensis* Poir. and *Bunchosiaglandulifera* (Jacq.) Kunth by N. Rakotonirina and R.F. Almeida, respectively. Hiraeeae: flower of *Hiraearestingae* C.E.Anderson by R.F. Almeida. Hiptageae: flowers of *Niedenzuellamultiglandulosa* (A.Juss.) W.R.Anderson, *Hiptagebenghalensis* (L.) Kurz, *Christianellamultiglandulosa* (Nied.) W.R.Anderson, *Aliciaanisopetala* (A.Juss.) W.R.Anderson, and *Callaeumpsilophyllum* (A.Juss.) D.M.Johnson all by R.F. Almeida. Malpighieae: flowers of *Amorimiaandersonii* R.F.Almeida, *Mascagniacordifolia* (A.Juss.) Griseb., *Aspidopteryswallichii* Hook.f., *Triaspismacropteron* Welw. ex Oliv., and *Madagasikariaandersonii* C.Davis by F. Michelangeli, M.O.O. Pellegrini, N. Singh, E. Bidault, and C.C. Davis, respectively. Gaudichaudieae: flowers of *Bronweniamegaptera* (B.Gates) W.R.Anderson & C.Davis, *Diplopteryslutea* (Ruiz ex Griseb.) W.R.Anderson & C.Davis, *Stigmaphyllonparalias* A.Juss., *Peixotoahispidula* A.Juss., *Camareaaxillaris* A.St.-Hil., and *Mamedeapulchella* (Griseb.) R.F.Almeida & M.Pell. by R.F. Almeida, M.O.O. Pellegrini, M.O.O. Pellegrini, R.F. Almeida, R.F. Almeida, and N. Taniguti, respectively.

In the past two decades, Malpighiaceae has gone through unparalleled changes in its traditional classification as a direct result of several molecular phylogenetic studies (Cameron et al. 2001; Davis et al. 2001; Davis and Anderson 2010; Almeida et al. 2017, 2018, 2023a, b; Almeida and van den Berg 2021, 2022). Key morphological characters of its traditional classification system (i.e., fruit types) were shown to be highly homoplastic (Cameron et al. 2001; Davis et al. 2001). The inevitable recognition of unforeseen relationships within Malpighiaceae brought to light deep taxonomic issues regarding the monophyly of several genera [e.g., *Banisteriopsis* C.R.Rob., *Mascagnia* (Bertero ex DC.) Bertero, *Stigmaphyllon* A.Juss., and *Tetrapterys* Cav.], tribes (i.e., only Gaudichaudieae was recovered as monophyletic), and all its subfamilies (i.e., Byrsonimoideae and Malpighioideae) (Cameron et al. 2001; Davis and Anderson 2010; Davis et al. 2001). Since then, different authors have gradually proposed new genera and combinations to accommodate these newly identified lineages (Anderson 2006; Anderson and Davis 2006; Anderson 2011; Davis et al. 2020; Almeida and van den Berg 2021; Almeida et al. 2023a, b). Even though morphological characters were used to reconstruct the last generic phylogeny of Malpighiaceae, no morphological characters were ever recovered and/or discussed for its major clades (Almeida et al. 2023a, b). As a result, its traditional classification was rejected, and several informal clades, without any morphological circumscription, were recognised in the most recent generic phylogeny for Malpighiaceae: 1. Byrsonimoids, 2. Acridocarpoids, 3. Mcvaughioids, 4. Barnebyoids, 5. Ptilochaetoids, 6. Bunchosioids, 7. Hiraeoids, 8. Tetrapteroids, 9. Malpighioids, and 10. Stigmaphylloids (Fig. 1; Davis and Anderson 2010; Almeida et al. 2023a).

As a result of over ten years of integrative studies worldwide, and based on a comprehensive review of the last twenty years of published studies for this family, we present a new classification system for Malpighiaceae based on molecular phylogenetics, morphology, palynology, and chemistry. We also present a taxonomic synopsis, including descriptions or diagnoses for the two subfamilies and 12 tribes currently recognised, besides identification keys for subfamilies, tribes, and genera. Some genera are re-circumscribed, while others are proposed, together with the necessary new combinations. Several taxonomic changes are proposed for subfamilial, tribal, generic, and species ranks. A checklist of all currently accepted species of this family is also presented alongside their extinction threat risk (Suppl. material 1).

## ﻿Materials and methods

### ﻿Phylogenetics

We sampled one to three species and all 72 accepted Malpighiaceae genera recognised by us in the taxonomic treatment. The aligned matrix for *matK*, *ndhF*, *rbcL*, and PHYC from Davis and Anderson (2010) was retrieved from TreeBase (accession no. 10998) and reduced to our proposed sampling and edited using Geneious (Kearse et al. 2012) for gaps removal made manually by eye. All trees were rooted in Elatinaceae (*Bergia* + *Elatine*), the sister-group of Malpighiaceae, according to Davis and Anderson (2010). A combined analysis of plastid + nuclear regions was carried out using Maximum Likelihood (ML). We selected the model using hierarchical likelihood ratio tests (HLRT) on J Modeltest 2 (Darriba et al. 2012). The model-based method was conducted with a mixed model (GTR+G+I) and unlinked parameters using RAxML 8 (Stamatakis 2014) implemented on RAxMLGUI2 (Edler et al. 2021). ML analyses were performed with ten independent replicates, and default settings and support values were estimated using parametric bootstrapping with 500 replicates, with bootstrap support values presented on nodes. Character coding followed the recommendations for morphological analyses of Sereno (2007). Primary homology hypotheses (De Pinna 1991) were proposed for leaf, inflorescence architecture, floral, pollen, fruit, and chromosomic characters. A total of 31 characters were scored and coded (Suppl. material 2). All characters were optimised on the concatenated tree with the Maximum Likelihood function (mk1 model) using Mesquite 2.73 (Maddison and Maddison 2006) and visualised on Winclada (Nixon 1999).

### ﻿Taxonomy

The analyses of morphological data were based on specimens from the following herbaria: ALCB, AMAZ, ASE, BAH, BM, BHCB, BOTU, CEN, CEPEC, CESJ, CGMS, COL, CPAP, CVRD, CTES, CUZ, EAC, ESA, F, FLOR, FUEL, FURB, FZB, G, GH, GUA, HAS, HB, HCF, HEPH, HISA, HRB, HRCB, HSJRP, HST, HUCP, HUCS, HUEFS, HUEM, HUESC, HUFG, HUFU, HUPG, HURB, HUT, HUVA, IAC, IAN, ICN, INPA, IPA, JPB, L, LIL, K, MAC, MBM, MBML, MG, MICH, MO, MOSS, MPU, NY, OUPR, P, PACA, PAMG, PEUFR, PMSP, R, RB, RBR, RFA, PRE, S, SI, SMF, SP, SPF, SPSF, TEPB, U, UB, UEC, UFP, UFMS, UFMT, UFRN, UPCB, US, USZ, W, VIC, and VIES (acronyms according to Thiers, continuously updated). Indumentum terminology and structure shapes follow Almeida and Morais (2022), inflorescence terminology and morphology follow Weberling (1965, 1989), and fruit terminology follows Spjut (1994). Chromosome numbers were retrieved from Anderson (1993) and Lombello and Forni-Martins (2002, 2003), and secondary metabolites were retrieved from Mannochio-Russo et al. (2022). Palynological data were retrieved from Lowrie (1982), Silva et al. (2023), and Almeida et al. (2024) and complemented by the slide collections from the Instituto de Pesquisas Ambientais (São Paulo, Brazil) and the Royal Botanic Gardens, Kew.

## ﻿Results

### ﻿Phylogenetics

All ten major informal phylogenetic clades of Malpighiaceae recognised in the taxonomy section were well-supported by bootstrap values ranging from 60–100% (Fig. 1). *Rhynchophora* was the only genus recognised here recovered as paraphyletic due to the positioning of *Madagasikaria* nested within it (Fig. 1). *Ectopopterys* was the only genus not confidently placed within one of the ten major clades in Malpighiaceae, with it being positioned as the first lineage to diverge in Malpighioids or Stigmaphylloids depending on the number of species sampled within each genus (Fig. 1). Subfamily Byrsonimoideae was supported by one homoplasy (connective glands prominent) and two synapomorphies (styles subulate and stigmas punctiform; Fig. 1). Tribe Acmanthereae was supported by two synapomorphies (leaf venation camptodromous and carpels free), tribe Galphimieae by one homoplasy (bracteoles glandular), and tribe Byrsonimeae by two homoplasies (leaves eglandular and petals cucullate; Fig. 1). Subfamily Malpighioideae was supported by one homoplasy (androecium heteromorphic) and one synapomorphy (winged mericarps; Fig. 1). Tribe Acridocarpeae was supported by four synapomorphies (stipules absent, leaves alternate, bracts glandular, and styles reflexed in fruit) and two homoplasies (inflorescence main axis deflexed and styles two; Fig. 1). Tribe Mcvaughieae was supported by one synapomorphy (2–7-flowered cincinni) and three homoplasies (bracteoles glandular, petals cucullate, and drupes; Fig. 1). Tribe Barnebyeae was supported by a single homoplasy (2-flowered cincinni), while tribe Ptilochaeteae by one synapomorphy (leaf blades revolute when young) and one homoplasy (only 4 1-flowered cincinni; Fig. 1). Tribe Bunchosieae was supported by two homoplasies (stipules epipetiolar and mericarps without wings; Fig. 1), while tribe Hiraeeae by one homoplasy (stipules epipetiolar) and one synapomorphy (leaf blades glandular at apex; Fig. 1). Tribe Hiptageae was supported by three homoplasies (stipules inconspicuous, petals pubescent, and stigmas lateral), while tribe Malpighieae by a single homoplasy (stigmas lateral; Fig. 1). Finally, tribe Gaudichaudieae was supported by a single homoplasy (connective glands prominent; Fig. 1).

### ﻿Taxonomy

Despite *Madagasikaria* causing the non-monophyly of *Rhynchophora* (Fig. 1), the bootstrap support value for this clade is below 60%. Therefore, we have chosen to retain both genera as independent until further phylogenetic evidence sheds some light on the matter. Regarding the placement of *Ectopopterys*, which is either recovered as sister to the Malpighioids or the Stigmaphylloids, we have chosen what we believe to be the most parsimonious approach. Since the clade that includes the Malpighioids + *Ectopopterys* is supported by morphology, we have tentatively retained it as a member of that clade to prevent the unnecessary recognition of another monogeneric tribe. Finally, at this time, we refrain from recognising subtribes despite the large size of several tribes. This is due to the generally low statistical support for the relationships within the tribes or the lack of morphological characters circumscribing internal clades. Therefore, all previously proposed subtribes are temporarily treated under the synonymy of the tribe they are members of until future phylogenetic/omic studies shed some needed light on this matter.

#### ﻿ Malpighiaceae

Taxon classificationPlantaeMalpighialesMalpighiaceae

Juss., Gen. Pl.: 252. 1789 nom. cons.

28872965-D674-5D43-B1CA-4594B3A92622

Figs 2 , 3 , 4 , 5 , 6 , 7 , 8 , 9 , 10 , 11

##### Type species.

*Malpighiaglabra* L.

##### Description.

**Figure 2.**
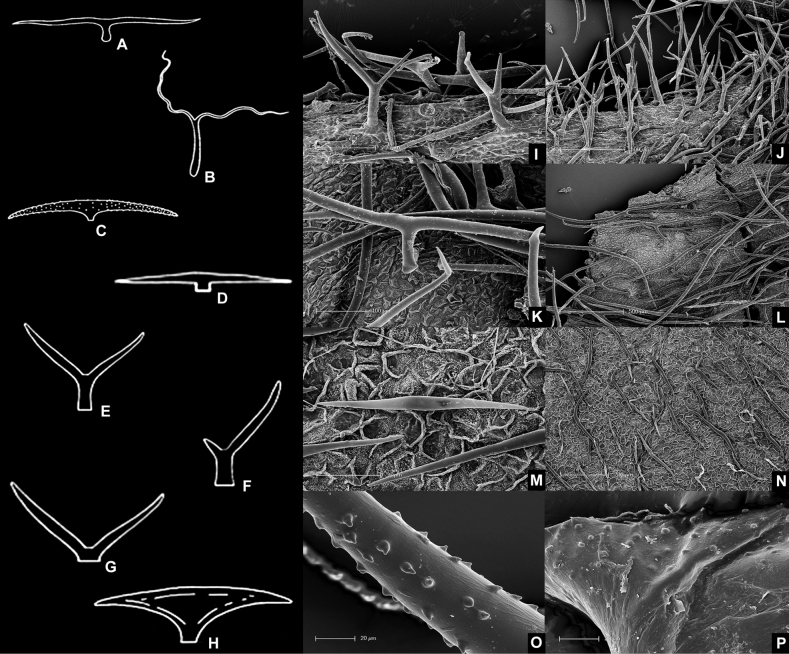
Line drawings and scanning electron micrographs of common types of malpighiaceous hairs **A** T-shaped with a short base (i.e., foot) **B** T-shaped with a long base **C** T-shaped with a very reduced base and branches with spiked cell wall **D** T-shaped with a very reduced base and branches with smooth cell wall **E** Y-shaped with a long base and two equally long branches **F** Y-shaped with a long base, one long and one very reduced branch **G** Y-shaped with a very reduced base **H** T-shaped with reduced base and laterally flattened (i.e., scaly) **I, J** detail of a velutine indumentum comprising Y-shaped hairs **K, L** detail of a tomentose indumentum comprising T-shaped hairs with long bases **M, N** detail of a sericeous indumentum comprising T-shaped hairs with very reduced bases **O** detail of the spikes on the cell wall of a hair branch **P** detail of the rugae on the cell wall of a hair branch (all line drawings and SEMs by R.F. Almeida).

**Figure 3.**
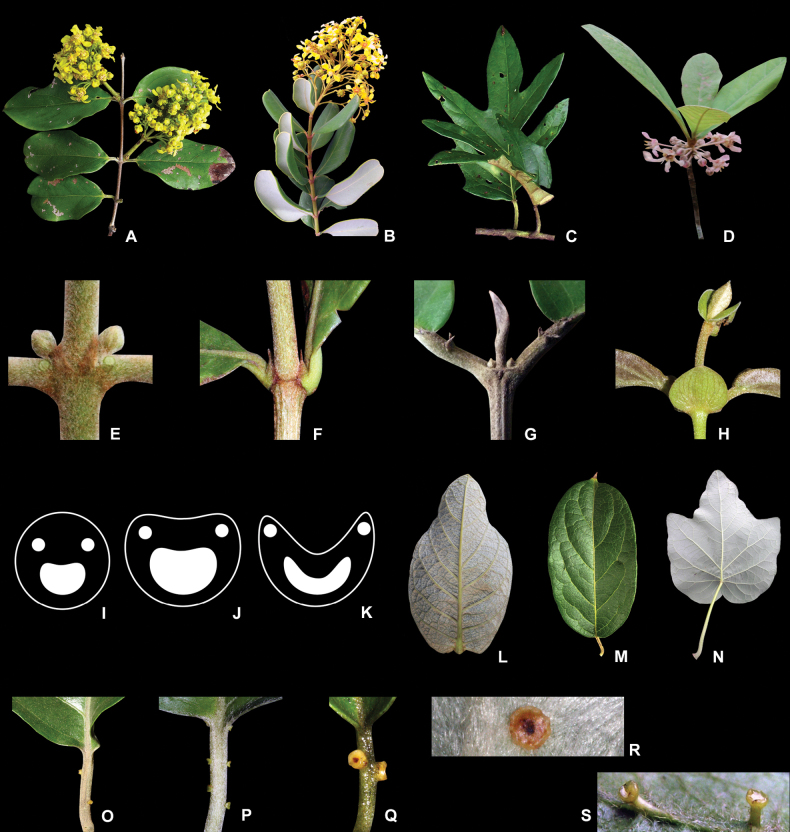
Phyllotaxis, stipules, and petioles of Malpighiaceae**A** branch with opposite leaves of *Bronweniamegaptera***B** branch with decussate leaves of *Verrucularinaglaucophylla***C** branch with alternate leaves of *Stigmaphyllonangustilobum***D** branch with verticillate leaves of *Pterandrapyroidea***E** interpetiolar stipules of *Mascagniacordifolia***F** epipetiolar stipules of *Byrsonimaintermedia***G** free stipules of *Hiraeahatschbachii***H** connate stipules of *Peixotoacatarinensis***I** transverse section of a circular petiole **J** transverse section of a plane-convex petiole **K** transverse section of a canaliculate petiole **L** leaf with very reduced petiole of *Byrsonimabasiloba***M** leaf with short petiole of *Banisteriopsisadenopoda***N** leaf with long petiole of *Stigmaphylloncaatingicola***O** alternate glands on the petiole of *Banisteriopsismembranifolia***P** opposite to alternate glands on the petiole of *Schwanniamediterranea***Q** subopposite glands on the petiole of *Banisteriopsismembranifolia***R** discoid and sessile gland of *Banisteriopsislaevifolia***S** cupuliform and stalked glands of *Banisteriopsisadenopoda* (line drawings and photographs **A–C, G, I–K, L–O, Q–S** by R.F. Almeida; **D** by C. Silva, **E, F, H, P** by M.O.O. Pellegrini).

**Figure 4.**
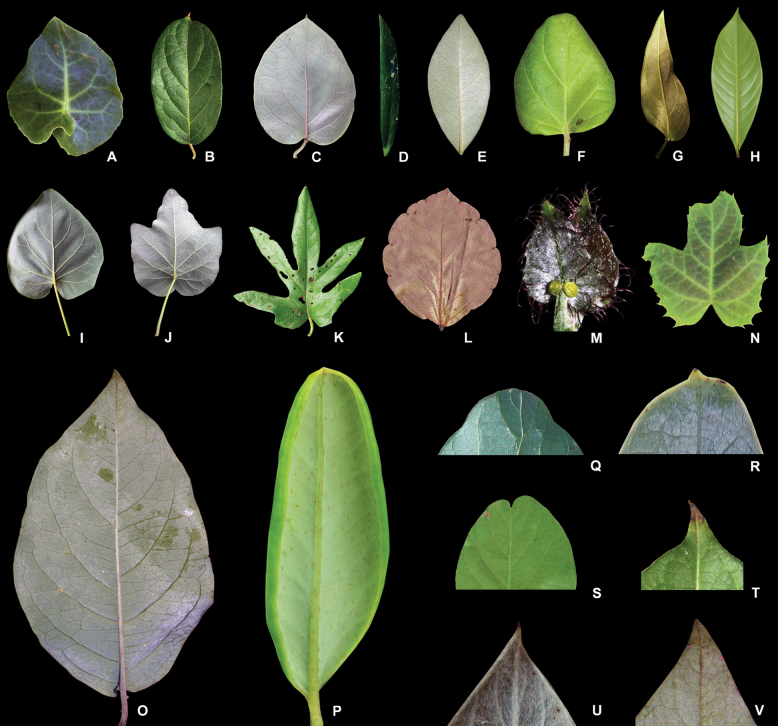
Leaf blades of Malpighiaceae**A** leaf with sagittate base of *Stigmaphyllonciliatum***B** leaf with rounded base of *Banisteriopsisadenopoda***C** leaf with cordate base of *Stigmaphyllonblanchetii***D** leaf with cuneate base of *Banisteriopsisvernoniifolia***E** leaf with obtuse base of *Stigmaphyllonparalias***F** leaf with truncate base of *Stigmaphyllongayanum***G** leaf with oblique base of *Stigmaphyllonlanceolatum***H** leaf with attenuate base of *Acmantheraminima***I** leaf with entire margin of *Stigmaphylloncaatingicola***J** leaf with 3-lobed margin of *Stigmaphylloncaatingicola***K** leaf with 5-lobed margin of *Stigmaphyllonangustilobum***L** leaf with crenate margin of *Stigmaphylloncrenatum***M** leaf with ciliate margin of *Stigmaphyllonciliatum***N** leaf with dentate margin of *Stigmaphyllonvitifolium***O** leaf with plane blade margin of *Banisteriopsismembranifolia***P** leaf with revolute blade margin of *Verrucularinaglaucophylla***Q** rounded leaf apex of *Tetrapterysphlomoides***R** mucronate leaf apex of *Banisteriopsismagdalenensis***S** emarginate leaf apex of *Hiraeacuiabensis***T** cuspidate leaf apex of *Banisteriopsisadenopoda***U** acuminate leaf apex of *Mamedeaharleyi***V** acute leaf apex of *Banisteriopsismembranifolia* (photographs **A–D, F, G, I, L, O, P, T–V** by R.F. Almeida; **E, M** by M.O.O. Pellegrini; **H** by F. Farronay, **Q** by G.A. Dettke, **N** by A.C. Dal Col, **R** by C. Baez, **S** by I.L. Morais).

**Figure 5.**
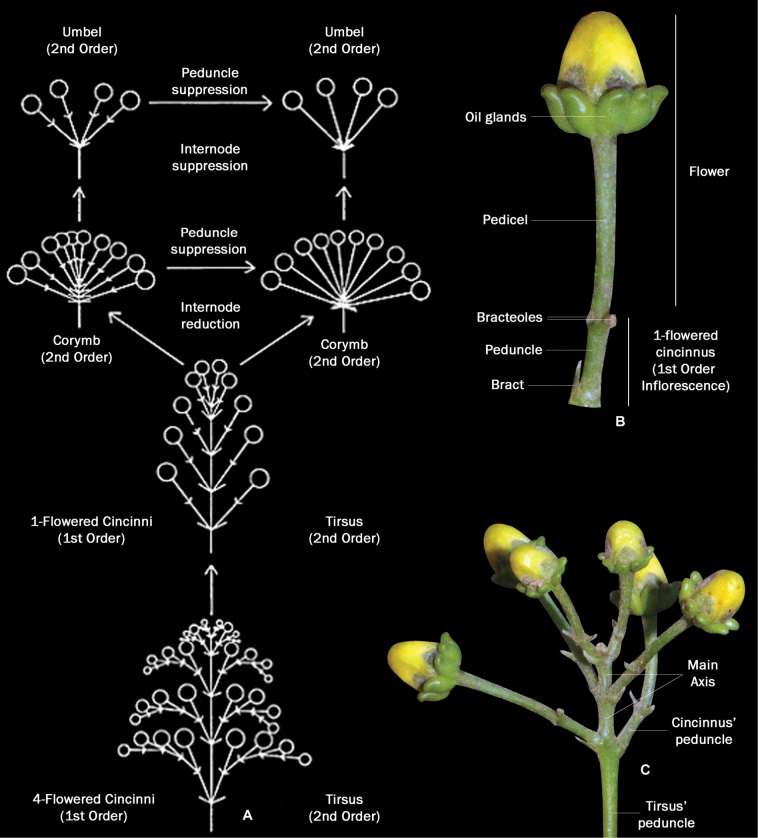
Inflorescence architecture of Malpighiaceae**A** inflorescence evolution in Malpighiaceae according to Anderson (1981)**B** 1-flowered cincinnus of *Niedenzuellalasiandra***C** Thyrse of 1-flowered cincinni of *Niedenzuellalasiandra* (line drawings modified from Anderson 1981; photographs **A** modified from Anderson 1981; **B, C** by M.O.O. Pellegrini).

**Figure 6.**
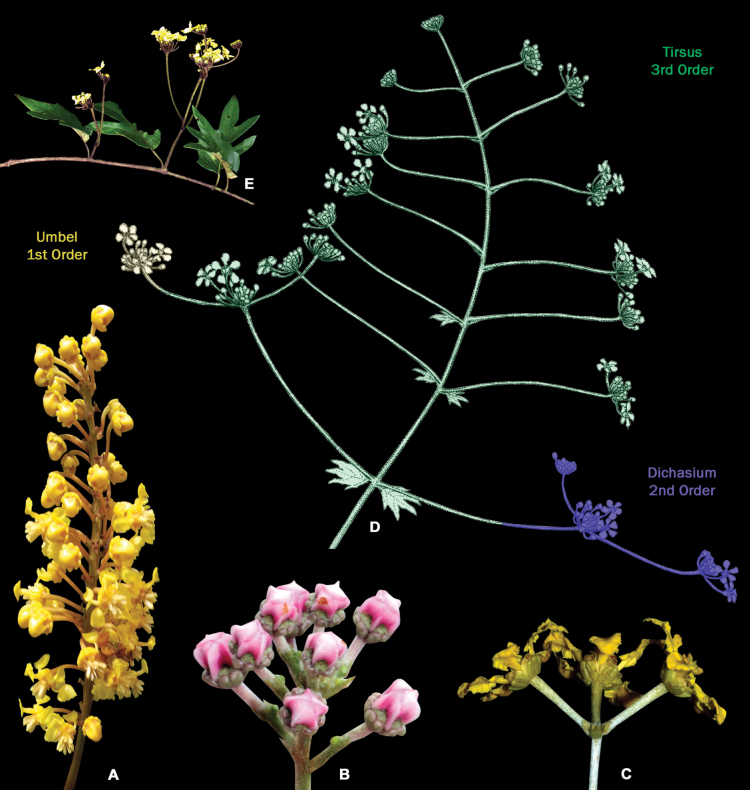
Compound inflorescences of Malpighiaceae**A** thyrse of 1-flowered cincinni of *Byrsonimasericea***B** corymb of 1-flowered cincinni of *Mascagniacordifolia***C** umbel of 1-flowered cincinni of *Banisteriopsisargyrophylla***D** line drawing of an inflorescence of *Stigmaphyllonangustilobum* showing 1-flowered cincinni arranged in umbels (1^st^ order inflorescence), arranged in dichasia (2^nd^ order inflorescence), arranged in a thyrse (3^rd^ order inflorescence) **E** photograph of the inflorescence branch of *Stigmaphyllonangustilobum* (photographs **B, C** by M.O.O. Pellegrini; **A, E** by R.F. Almeida; line drawing **D** by K. Souza).

**Figure 7.**
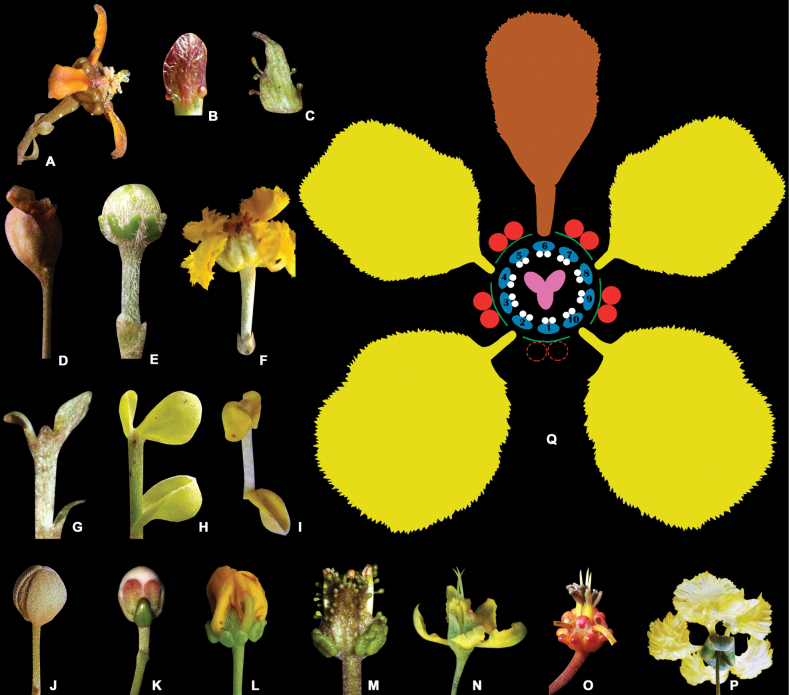
Flowers of Malpighiaceae**A** side view of the flower of *Amorimiacoriacea* showing the cincinnus peduncle with a bract at base and two bracteoles at apex **B** detail of the 2-glandular bracteoles of *Glicophyllumcardiophyllum***C** details of the glandular margin of the bracteole of *Christianellamultiglandulosa***D** bracteoles of *Meziaaraujoi* concealing the floral bud at pre-anthesis (floral pedicel is absent) **E** floral bud of *Niedenzuellamultiglandulosa*, showing a very short peduncle with a bract at base and two bracteoles at apex **F** flower of *Banisteriopsislaevifolia* showing pedicel with bract and bracteoles at base (peduncle absent) **G** plane and patent bract and bracteoles of *Aliciaanisopetala***H** cucullate bract and bracteoles of *Dicellabracteosa***I** deflexed bract and bracteoles of *Dicellanucifera***J** eglandular sepals concealing petals at pre-anthesis in *Thryallislongifolia***K** 1-glandular calyx of *Hiptagebenghalensis***L** 10-glandular calyx of *Camareaaxillaris***M** 8-glandular calyx of *Christianellamultiglandulosa* showing the multi-glandular margin of sepals **N** erect sepals of *Galphimiaaustralis***O** revolute apex of sepals of *Byrsonimabasiloba***P** revolute and reflexed sepals of *Thryallislongifolia***Q** floral diagram of a Malpighiaceae flower with sepals in green, sepal glands in red, lateral petals in yellow, posterior petal in brown, androecium in blue (connectives) and white (pollen sacs), and gynoecium in pink (diagram and photographs **B, E, F, H, K, L, N, O, Q** by R.F. Almeida; **A–D, G, M** by M.O.O. Pellegrini; **I** by Amaury Jr.; **J, P** by J.V. Santos).

**Figure 8.**
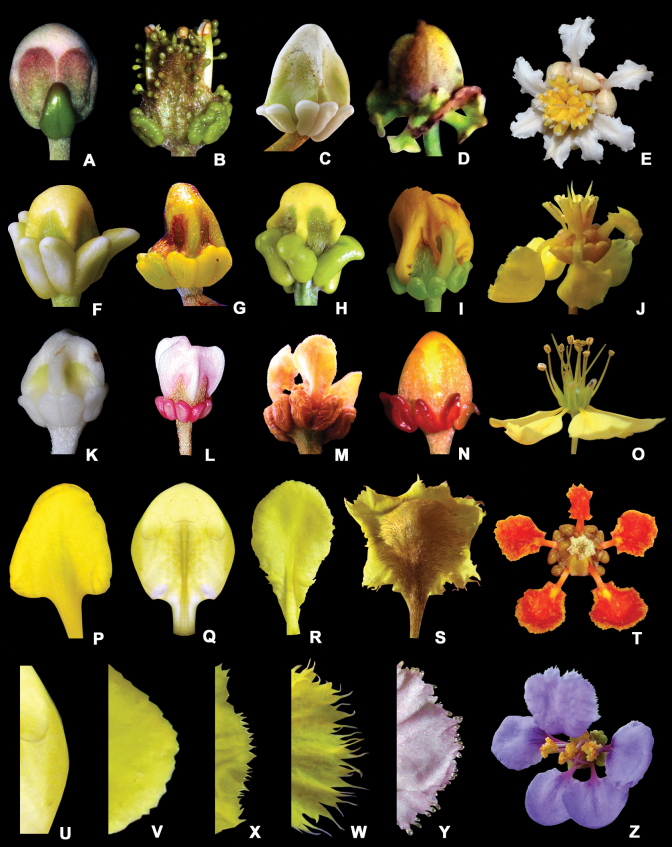
Sepals, glands, and petals of Malpighiaceae flowers **A** sepals with rounded apex of *Hiptagebenghalensis* and sessile sepal gland **B** sepals with acute apex of *Christianellamultiglandulosa* and sessile sepal glands **C** sepals with acute apex of *Byrsonimagardneriana* and sessile sepal glands **D** stalked sepal glands of *Heladenamultiflora***E** white petals of *Acmantheralatifolia***F** pale yellow glands of *Bunchosiaglandulifera***G** yellow glands of *Mcvaughiasergipana***H** pale green glands of *Bronweniamegaptera***I** green glands of *Camareaaxillaris***J** yellow and deflexed petals of *Byrsonimasericea***K** white glands of *Acmantheraparviflora***L** pink glands of *Heteropterysrubiginosa***M** brown glands of *Amorimiapellegrinii***N** red glands of *Niedenzuellapoeppigiana***O** yellow and patent petals of *Ptilochaetabahiensis***P** oval petal limb of *Galphimiagracilis***Q** elliptic petal limb of *Heteropterysoberdanii***R** obovate petal limb of *Bronweniamegaptera***S** pubescent petal surface of *Diplopterysbahiana***T** orange-red petals of *Tetrapterysphlomoides***U** entire margin of the petal of *Heteropterysoberdanii***V** erose margin of the petal of *Bronweniamegaptera***W** fimbriate margin of the petal of *Schwanniaschwannioides***X** dentate margin of the petal of *Peixotoahispidula***Y** glandular-fimbriate margin of the petal of *Aliciaanisopetala***Z** lilac petals of *Mascagnialilacina* (**A, C, F–H, I, J, L–S, U–W** by R.F. Almeida; **B, N, T, Y** by M.O.O. Pellegrini; **D** by A. Francener; **E** by R. Goldenberg; **Z** by O.J.A. Ayala).

**Figure 9.**
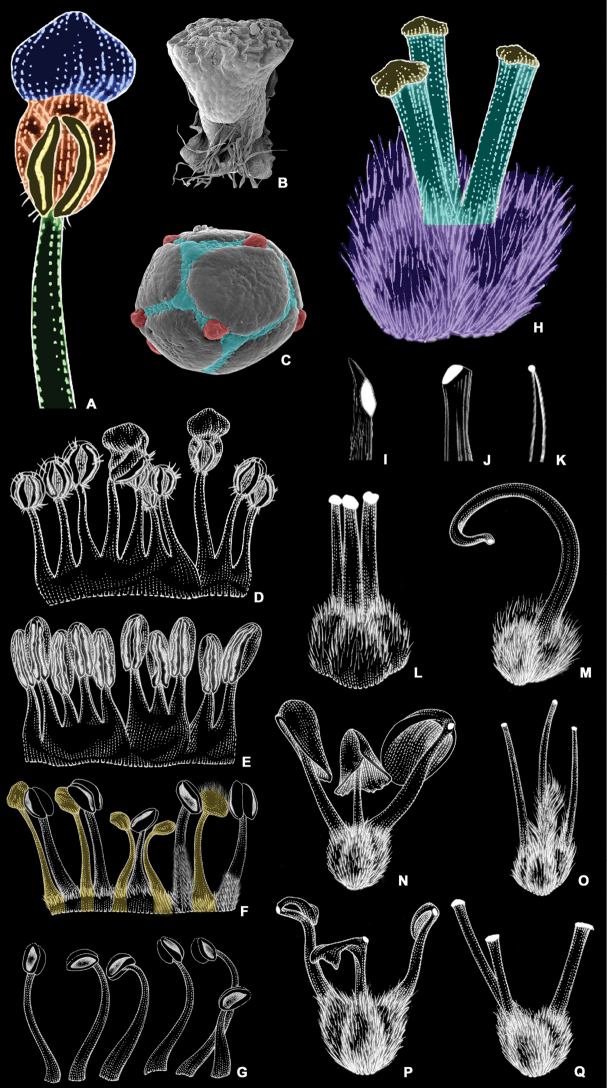
Androecium and gynoecium of Malpighiaceae**A** stamen of *Banisteriopsismultifoliolata* showing filament in green, anther in orange, glandular connective in blue, and pollen sacs in yellow **B** scanning electron micrograph of an anther of *Banisteriopsismultifoliolata***C** SEM of a pollen grain of *Banisteriopsismultifoliolata* showing colpi in blue and endoaperture in red **D** heteromorphic stamen ring with 10 fertile stamens of *Banisteriopsismultifoliolata***E** homomorphic stamen ring of *Bronweniamegaptera* with 10 fertile stamens **F** heteromorphic stamen ring of *Peixotoahispidula* showing 5 fertile stamens and 5 staminodes in yellow **G** 6 fertile and free stamens of *Schwanniahexandra***H** gynoecium of *Banisteriopsismultifoliolata* showing ovary in lilac, styles in blue and stigmas in yellow **I** uncinate apex of styles of *Amorimiaseptentrionalis***J** truncate style apex of *Amorimiarigida***K** subulate apex of style of *Byrsonimasericea***L** 3 parallel and erect styles of *Bronweniamegaptera***M** single curved style of *Schwanniahexandra***N** 3 divergent styles of *Stigmaphyllonblanchetii* with foliate apices **O** 3 erect, slightly curved, and pubescent styles of *Diplopteryslutea***P** 3 divergent styles of *Stigmaphyllonlalandianum* with reduced foliate apices **Q** 3 divergent styles of *Stigmaphyllonglabrum* without foliate apex (all line drawings by K. Souza; SEMs by R.F. Almeida; all scales: 1 mm, except for the pollen grain: 10 µm).

**Figure 10.**
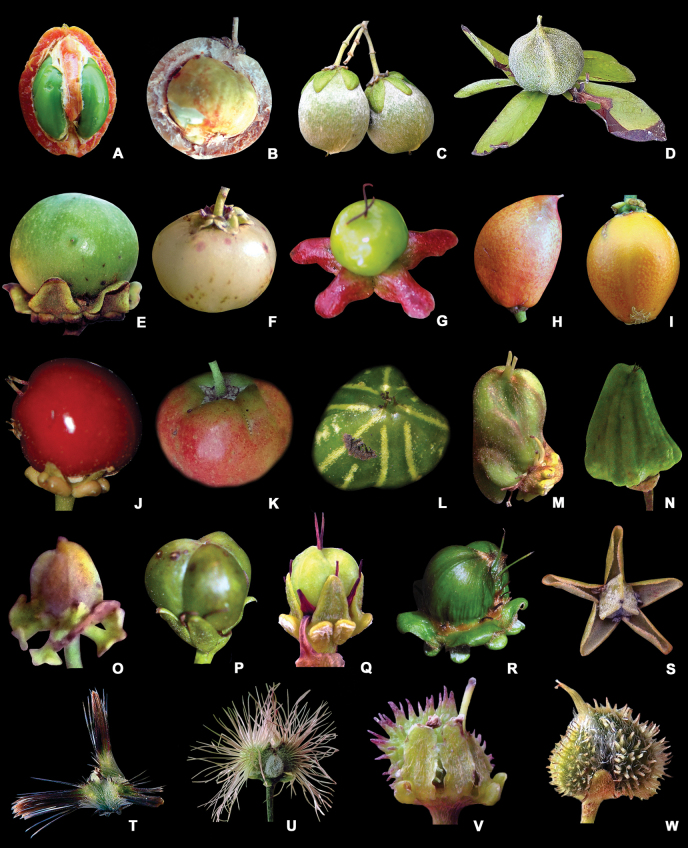
Types of fleshy fruits, nuts, and smooth to setose schizocarpic fruits in Malpighiaceae**A** transversely sliced drupe of *Bunchosiamaritima* showing seeds **B** transversely sliced nut of *Dicellanucifera* showing the seed **C** nuts of *Dicellabracteosa***D** nuts of *Dicellamacroptera***E** green drupe of *Byrsonimablanchetiana***F** cream-coloured drupe of *Byrsonimaligustrifolia***G** green drupe of *Byrsonimamelanocarpa* with concrescent sepals **H** orange drupe of *Bunchosiaglandulifera***I** orange drupe of *Bunchosiamaritima***J** red drupe of *Malpighiaglabra***K** reddish-orange drupe of *Malpighiamexicana***L** green drupe of *Malpighiafucata***M** green and twisted drupe of *Mcvaughiasergipana***N** striated drupe of *Burdachiaprismatocarpa***O** smooth mericarp of *Heladenamultiflora***P** smooth mericarp of *Galphimiagracilis***Q** smooth and immature mericarp of *Verrucularinaglaucophylla***R** green and smooth mericarps of *Acmantheralatifolia***S** smooth mericarps of *Thryallislongifolia* with concrescent sepals **T** setose mericarps of *Tricomariausillo***U** setose mericarps of *Lasiocarpusferrugineus***V** setose mericarp of *Camareaaxillaris***W** setose mericarp of *Echinopteryseglandulosa* (photographs **A, B** by Amaury Jr.; **C, D** by A. Assis; **E, H, I, M, Q, V** by R.F. Almeida; **F** by S.E. Martins; **G** by N. Bigio; **J** by P. Acevedo-Rodriguez; **K, L** by M.R. Pace; **N** by L.S.B. Calazans; **O** by A. Francener; **P** by M.O.O. Pellegrini; **R** by R. Goldenberg; **S** by J.V. Santos; **T** by I. Specogna; **U** by A. Nuno; **W** by S. Carnaham).

**Figure 11.**
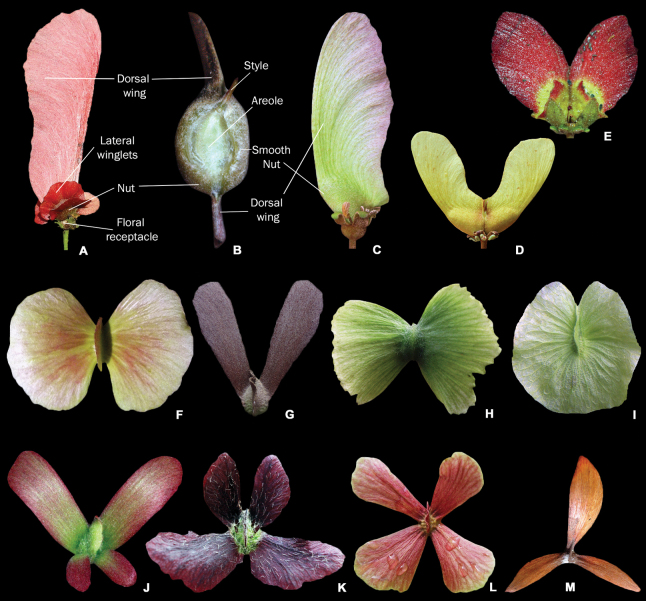
Types of winged schizocarpic fruits in Malpighiaceae**A** single dorsal winged mericarp of *Diplopteryspubipetala* showing lateral wings **B** detail of part of the winged mericarp of *Banisteriopsisargyrophylla***C, D** 1 dorsal winged mericarp(s) of *Heteropterysbyrsonimifolia***E** winged mericarp of *Peixotoacatharinensis* showing lateral winglets **F** winged mericarp of *Amorimiacandidae* with two lateral wings more developed than the reduced dorsal wing **G** winged mericarp of *Lophopterysfloribunda***H** winged mericarp of *Caroluschasei* with two lateral wings more developed (dorsal wing absent) **I** winged mericarp of *Mascagniasepium* with 1 lateral orbicular wing **J** winged mericarp of *Tetrapterysphlomoides* with 4 free, lateral wings (superior ones longer, inferior ones shorter) **K** winged mericarp of *Glicophyllumcardiophyllum* with 4 free, lateral wings (superior ones shorter, inferior ones longer) **L** winged mericarp of *Niedenzuellaacutifolia* with 4 free, equalling lateral wings **M** winged mericarp of *Hiptagebenghalensis* with three free, lateral wings more developed (photographs **A** by A. Popovkin; **B, E, I, J** by M.O.O. Pellegrini; **C, D, F, H, K, L** by R.F. Almeida; **G** by G. Shimizu; **M** by G. Cahyadi).

**Trees, shrubs, subshrubs** (erect, monopodial or scandent) or **lianas**, monoecious, rarely functionally dioecious or androdioecious, perennial; hairs unicellular, foot present, conspicuous or not, 2-branched (malpighiaceous), T-, Y- or V-shaped, rarely acicular or stellate, branches straight, undulate, or curled, surface smooth, rough or spiny (Fig. 2). ***Roots*** fibrous or tuberous, generally becoming woody with age. ***Xylopodium*** present or not. ***Branches*** woody, rarely herbaceous, lenticelate or not. ***Stipules*** present, rarely absent, inter- or epipetiolar, minute to expanded, free to connate, pair of stipules free or connate, deciduous or persistent (Fig. 3E–H). ***Leaves*** opposite, decussate, rarely verticillate, ternate, subopposite or alternate (Fig. 3A–D), petiolate; petioles short to very long (Fig. 3L–N), circular, plane-convex to canaliculate (Fig. 3I–K) in cross-section, usually glandular (Fig. 3O-S); blades simple, entire, rarely lobed (Fig. 4I–K), usually glandular, margin plane to revolute (Fig. 4O, P), entire, sometimes dentate, crenate or lobulate, glabrous, ciliate or pubescent (Fig. 4I–N). ***Inflorescences*** solitary or compound, terminal to axillary, pedunculate, rarely sessile (Fig. 5A); flowers arranged in a 1–multi-flowered cincinnus (Fig. 5B), cincinni opposite to alternate, usually pedunculate (Fig. 5C), rarely sessile, solitary or arranged in 2–5-degrees of ramification into thyrses, corymbs, umbels, or dichasia (Fig. 6); leaves associated with the inflorescences similar to vegetative leaves but reduced in size (Fig. 6D); cincinni bract 1, minute to expanded, plane or concave, persistent, rarely deciduous, glandular or eglandular (Fig. 7A–I); bracteoles 2, opposite or alternate, usually inserted at the apex of peduncles, rarely subapical, medial or basal, minute to expanded, plane or concave, persistent, rarely deciduous, glandular or eglandular (Fig. 7A–I). ***Flowers*** chasmogamous, rarely cleistogamous, bisexual, rarely unisexual, zygomorphic, rarely actinomorphic, hypogynous, rarely perigynous (Fig. 7Q); pedicel well-developed, rarely absent or inconspicuous, straight, rarely circinate (Fig. 7D–F); sepals 5(–7), free to connate at base, imbricate in bud, rarely valvate, erect, rarely deflexed, apex erect to revolute, persistent in fruit, usually not accrescent in fruit, rarely accrescent, abaxially (0–)1–2(–many)-glandular, sometimes the anterior sepal eglandular, rarely all sepals eglandular (Fig. 7J–L), glands multicellular, usually sessile (Fig. 8C), rarely pedunculate (Fig. 8C), secreting oil (Fig. 8B), rarely nectar (Fig. 8A); petals 5(–7), free, imbricate in bud, rarely valvate, clawed, patent (Fig. 8O) or deflexed (Fig. 8J), usually yellow (Fig. 8F–J), sometimes white (Fig. 8C, E, K), pink (Fig. 8A, L, Y), orange (Fig. 8M) or red (Fig. 8T), rarely green, lilac (Fig. 8Z) or purple, usually changing colour after pollination, the posterior petal usually differing from the 4 lateral ones in size, colour, shape and/or presence of glands, limb generally crumpled in bud, plane, concave or cucullate at anthesis, margin entire (Fig. 8U), erose (Fig. 8V), denticulate (Fig. 8X), fimbriate (Fig. 8W), or glandular-fimbriate (Fig. 8Y); androecium with (3–5–6–)10 stamens (Fig. 9D–G), in two whorls, fertile stamens (2–6–)10 (Fig. 9E), staminodes 0(–2–5; Fig. 9F, G), filaments free (Fig. 9G) or connate (Fig. 9D, E) at base, rarely connate up to the middle, short to long, glabrous (Fig. 9E), rarely pubescent (Fig. 9F), homo- (Fig. 9E) or heteromorphic (Fig. 9D), anthers basifixed (Fig. 9A), connectives minute or expanded (Fig. 9A, B), usually glandular (secreting non-volatile oils, rarely essential oils; Fig. 9B), with or without an apical projection, glabrous (Fig. 9E) to pubescent (Fig. 9D), thecae 2, parallel, rarely divergent at base and connivant at apex, apex free, rarely confluent, rimose (Fig. 9A), rarely poricidal; gynoecium superior, (1–2–)3-carpelate (Fig. 9H), all fertile, rarely 1 abortive, carpels syncarpic, rarely basally syncarpic and apically apocarpic, locules 1-ovulate, ovules pendulous, anatropous, styles (1–2–)3 (Fig. 9L, M), free, rarely connate, basal, lateral, subapical or apical, straight (Fig. 9L), curved (Fig. 9O) or lyrate (Fig. 9N, P), apex subulate (Fig. 9K), cylindrical (Fig. 9L), laterally flattened, truncate (Fig. 9J) or uncinate (Fig. 9I), rarely expanded (Fig. 9N, P), stigmas terminal (Fig. 9L, M) or lateral (i.e., facing the centre of the flower or the posterior petal; Fig. 9I, N, P), subulate (Fig. 9K), punctiform (Fig. 9K), capitate (Fig. 9L) or crateriform (Fig. 9I). ***Fruits*** dry (Fig. 10O–W) or fleshy (Fig. 10A–N), schizocarps (Fig. 10O–W), nuts (Fig. 10B–D) or drupes (Fig. 10A, E–N), glabrous or pubescent, mericarps (1–2–)3, indehiscent or splitting at maturity, smooth (Fig. 10O–S), setose (Fig. 10T–W), or winged (Fig. 11A–M), when winged mericarps with 1 dorsal (Fig. 11A–E) and/or 1–several lateral (Fig. 11F–M) wings, free (Fig. 11F–H, J–M) or connate (Fig. 11I). ***Seeds*** 1 per locule (Fig. 10A), globose or ovoid (Fig. 10A), smooth (Fig. 10A) to rugose, without endosperm; embryo curved, bent or spiralled. Chromosome number *n* = 6–10.

##### Notes.

Malpighiaceae is here circumscribed with two subfamilies, 12 tribes, 72 genera, and 1,499 species accepted (Table 1; Suppl. material 1). From this total, 60 genera and 715 species are currently under some kind of extinction threat (Bachman et al. 2024), representing 84.5% of the accepted genera and 47.82% of the species in our study (Suppl. material 1). Most of Malpighiaceae’s diversity is confined to the American continent, with 55 genera (53 endemic) and 1,274 species (1,272 endemic), and just 15 genera (13 endemic) and 125 species (123 endemic) in Africa, seven genera (four endemic) and 84 species (77 endemic) in Asia, and four genera (all not endemic) and 21 species (19 endemic) in Oceania (Suppl. material 1). Most threatened species are found in the Americas (564 species), with 86 threatened species in Africa, 57 in Asia, and 12 in Oceania (Suppl. material 1). Five African genera, more specifically endemic to Madagascar (i.e., *Digoniopterys*, *Madagasikaria*, *Microsteira*, *Philgamia*, and *Rhynchophora*) stand out, with all their current accepted species under some kind of extinction threat (Suppl. material 1). *Mcvaughia* was the only American genus to present all its species under some kind of extinction threat (Suppl. material 1). *Malpighia* was the American genus with most species under some kind of extinction threat (Suppl. material 1). *Hiptage* was the genus with highest number of threatened species in Asia, and *Stigmaphyllon* was the most threatened in Oceania (Suppl. material 1).

**Table 1.** Classification system proposed for Malpighiaceae in the present study.

Malpighiaceae Juss.	
**Byrsonimoideae** W.R.Anderson	**Malpighioideae** Burnett emend. R.F.Almeida
**Acmanthereae** W.R.Anderson	**Acridocarpeae** R.F.Almeida
3 genera, 23 species	2 genera, 38 species
**Byrsonimeae** W.R.Anderson	**Mcvaughieae** R.F.Almeida
3 genera, 181 species	3 genera, 12 species
**Galphimieae** Nied.	**Barnebyeae** R.F.Almeida
3 genera, 40 species	1 genus, 2 species
	**Ptilochaeteae** R.F.Almeida
	3 genera, 10 species
	**Bunchosieae** R.F.Almeida
	5 genera, 122 species
	**Hiraeeae** A.Juss. emend. R.F.Almeida
	5 genera, 105 species
	**Hiptageae** DC. emend. R.F.Almeida
	17 genera, 377 species
	**Malpighieae** DC. emend. R.F.Almeida
	13 genera, 253 species
	**Gaudichaudieae** Horan emend. R.F.Almeida
	14 genera, 337 species

Identification keys for all subfamilies, tribes, and genera are presented, alongside a full morphological description for the proposed new genus, the recircumscription of ten genera accompanied by the needed new combinations, the proposition of several new synonyms, typification of miscellaneous names and notes on conservation, distribution, ecology, and taxonomy up to the genus rank.

### ﻿Key to the subfamilies of Malpighiaceae

**Table:** 

1	Posterior petal eglandular (Fig. 8O, P), androecium homomorphic (Fig. 9E), fertile stamens 10 (occasionally 6–10 in *Diacidia*), styles apex subulate (Fig. 9K), stigmas punctate (Fig. 9K); drupes or mericarps smooth (i.e., never winged or setose; Fig. 10A–S)	**Byrsonimoideae**
–	Posterior petal glandular (Fig. 8Y), androecium heteromorphic (Fig. 9D), fertile stamens 2–10, styles apex capitate, uncinate, truncate or expanded (Fig. 9L–Q), rarely subulate (Fig. 9K), stigmas capitate or crateriform (Fig. 9I, J, L), rarely punctate (Fig. 9K); nuts or mericarps, frequently winged (Fig. 11A–M) or setose (Fig. 10T–W), rarely smooth	**Malpighioideae**

#### ﻿1. Byrsonimoideae

Taxon classificationPlantaeMalpighialesMalpighiaceae

W.R.Anderson, Leandra 7: 6. 1977.

6198D022-3174-5C57-8657-29DC2439E647

##### Type genus.

*Byrsonima* Rich. ex Kunth.

##### Diagnosis.

Posterior petal eglandular, fertile stamens 10 (occasionally 6–10 in *Diacidia*), pollen 3-aperturate, zonoaperturate, colporate, styles apex subulate, mericarps smooth (i.e., never winged, or setose), chromosome number *n* = 6, presence of macrolactams and sulfenyl compounds.

##### Notes.

The subfamily Byrsonimoideae currently comprises the original three tribes published by Anderson (1977). However, tribe Byrsonimeae is re-circumscribed to exclude *Burdachia*, *Glandonia* and *Mcvaughia*, which made this tribe paraphyletic and are, thus, placed by us in their own tribe in Malpighioideae. In its new circumscription, Byrsonimoideae comprises nine genera and 243 species (91 threatened species; Suppl. material 1) of shrubs and trees endemic to the Americas.

### ﻿Key to the tribes of Byrsonimoideae

**Table:** 

1	Leaf veins camptodromous (Fig. 4H); sepals enclosing petals in bud (Fig. 8K), carpels free, styles basal, lateral or subapical (Fig. 9I, J)	**Acmanthereae**
–	Leaf veins brochidodromous (Fig. 4P); sepals not enclosing petals in bud (Fig. 8C), carpels connate, styles apical (Fig. 9K)	**2**
2	Leaves eglandular; bracteoles eglandular (Fig. 7G–I); petals smooth in bud (Fig. 8C), cucullate at anthesis (Fig. 8J); fruits indehiscent (Fig. 10E–G)	**Byrsonimeae**
–	Leaves glandular (except in *Verrucularina*); bracteoles glandular (Fig. 7B, C); petals keeled in bud, plane at anthesis (Fig. 8P), fruits dehiscent (Fig. 10P, Q)	**Galphimieae**

#### 1.1. Acmanthereae

Taxon classificationPlantaeMalpighialesMalpighiaceae

W.R.Anderson, Leandra 7: 7. 1977.

25294044-9483-5FE4-81AF-85EC9DAE92AD

##### Type genus.

*Acmanthera* (A.Juss.) Griseb.

##### Diagnosis.

Stipules absent, leaf veins camptodromous, sepals enclosing petals in buds, carpels free, styles ventrally to subapically inserted on ovaries, presence of diazanapaphthalenes, propargyl-type 1,3-dipolar organic compounds, absence of benzopyrans, lactams, lignam glycosides, pyrimidine nucleosides, pyrimidine nucleotides, saccharolipids, sulfenyl compounds.

##### Notes.

Acmanthereae currently comprises only three accepted genera (*Acmanthera*, *Coleostachys*, and *Pterandra*) and 23 species (15 threatened species; Suppl. material 1) of trees, shrubs, or subshrubs endemic to the Americas (POWO 2024).

### ﻿Key to the genera of Acmanthereae

**Table:** 

1	Leaves lanceolate; flowers sessile, sepals eglandular, anthers poricidal	***Coleostachys***
–	Leaves elliptic, ovate to obovate; flowers pedicellate, sepals glandular, anthers rimose	**2**
2	Flowers arranged in thyrses; sepals 2(–many)-glandular, petals 5(–7), glabrous, anthers with dorsal projections	***Acmanthera* (Figs 4H, 8E, K, 10R)**
–	Flowers arranged in umbels; sepals 0–2-glandular, petals 5, pubescent, anthers with lateral projections	***Pterandra* (Fig. 3D)**

#### ﻿1.1.1. Acmanthera

Taxon classificationPlantaeMalpighialesMalpighiaceae

(A.Juss.) Griseb. in Martius, Fl. Bras. 12(1): 28. 1858.

A1D711A1-A803-576D-9F67-3C43EE78A796

Figs 4H , 8E, K , 10R

≡ Pterandrasect.Acmanthera A.Juss., Ann. Sci. Nat. Bot., ser. 2, 13: 328. 1840.

##### Type species.

*Acmantheralatifolia* (A.Juss.) Griseb.

##### Notes.

*Acmanthera* currently comprises seven accepted species (four threatened species; Suppl. material 1) of trees, shrubs or subshrubs endemic to flooded forests of the Amazon rainforest, South America, and just a single species occurring within the Cerrado biome (Almeida et al. 2020; POWO 2024). For an identification key for all species of *Acmanthera*, see Anderson (1980) or Almeida et al. (2020).

#### ﻿1.1.2. Coleostachys

Taxon classificationPlantaeMalpighialesMalpighiaceae

A.Juss., Ann. Sci. Nat., Bot., sér. 2, 13: 329. 1840.

661D4F9B-F947-5BAE-8EBC-DD1D44D0048D

##### Type species.

*Coleostachysgenipifolia* A.Juss.

##### Notes.

*Coleostachys* is represented by a single species (not threatened; Suppl. material 1) of monopodial shrub endemic to non-flooded forests of the Amazon rainforest, South America (Almeida et al. 2020; POWO 2024). A comprehensive taxonomic revision was presented by Almeida and Hall (2016), but the information on type specimens presented by these authors was incomplete. Jussieu (1840) did not specify which specimen is the holotype nor in which herbarium it was deposited, therefore needing the lectotypification presented below.

#### ﻿1.1.2.a. Coleostachys genipifolia

Taxon classificationPlantaeMalpighialesMalpighiaceae

A.Juss., Ann. Sci. Nat., Bot., sér. 2, 13: 329. 1840.

634E7CCF-DC32-5AC6-92D0-8D48E1A7FE29

##### Lectotype (designated here).

French Guiana: Cayenne., s.d., *Martin s.n.* (P-JU barcode P00671745!; isolectotypes: BR barcode BR0000008577450!, F barcode V0062669F!, K barcode K000427026!, MICH barcode MICH1102137!, P barcodes P02428718!, P02428719!, P02428720!, P02428721!, RB barcode 540728!).

#### ﻿1.1.3. Pterandra

Taxon classificationPlantaeMalpighialesMalpighiaceae

A.Juss., Fl. Bras. Merid. (quarto ed.) 3(22): 72. 1832 [1833].

C409D6B9-F00F-58EA-934E-141FCB87BB3D

Fig. 3D

##### Type species.

*Pterandrapyroidea* A.Juss.

##### Notes.

*Pterandra* currently comprises 15 accepted species (11 threatened species; Suppl. material 1) of trees, shrubs or subshrubs endemic to non-flooded forests of the South American Amazon rainforest and Cerrado biomes, with just a single species occurring in non-flooded rainforests of Panama, Central America (Anderson 1997a; POWO 2024). For an identification key for all species of *Pterandra*, see Anderson (1997a).

#### 1.2. Byrsonimeae

Taxon classificationPlantaeMalpighialesMalpighiaceae

W.R.Anderson, Leandra 7: 11. 1977.

5B809C4C-6C9E-5EC4-8592-CB0EEC44D979

≡ Byrsoniminae Nied. in Engler, Nat. Pflanzenr. 92: 17, 28. 1928.

##### Type genus.

*Byrsonima* Rich. ex Kunth.

##### Diagnosis.

Stipules epipetiolar, leaves smaller than inflorescences, at least one petal cucullate at anthesis, presence of hydroxy acids and derivatives, imidolactams, keto acids and derivatives, organic phosphoric acids and derivatives, organofluorides, absence of oxanes.

##### Notes.

Byrsonimeae currently comprises only three accepted genera (*Blepharandra*, *Byrsonima*, and *Diacidia*) and 181 species (57 threatened species; Suppl. material 1) of trees, shrubs or subshrubs endemic to the Americas (POWO 2024).

### ﻿Key to the genera of Byrsonimeae

**Table:** 

1	Lateral petals deflexed at anthesis, anther connectives expanded, anthers without stiff hairs; mericarps drupaceous	***Byrsonima* (Figs 3F, L, 6A, 7O, 8C, J, 9K, 10E–G)**
–	Lateral petals patent at anthesis, anther connectives inconspicuous, anthers glabrous or with soft hairs; mericarps dry	**2**
2	Leaf base usually cordate, leaf apex rounded to emarginate; cincinni 2–3-flowered, sepals coriaceous, not accrescent in fruit, petals white, pink to red, anthers pubescent	***Blepharandra***
–	Leaf base rounded, leaf apex acute to acuminate; cincinni 1-flowered, sepals membranous, accrescent in fruit, petals yellow, anthers glabrous	***Diacidia***

#### ﻿1.2.1. Blepharandra

Taxon classificationPlantaeMalpighialesMalpighiaceae

Griseb., Linnaea 22: 7. 1849.

2F39D044-E8EA-5F38-8AEB-1019083520B5

= Callyntranthele Nied., Index Lect. Lyceo Braunsbergiensis 1897: 4. 1897. Type species: Callyntrantheleangustifolia (Kunth) Nied. [≡ Blepharandraangustifolia (Kunth) W.R.Anderson].

##### Type species.

*Blepharandrahypoleuca* (Benth.) Griseb.

##### Notes.

*Blepharandra* currently comprises six accepted species (one threatened species; Suppl. material 1) of trees or shrubs endemic to islands of savanna (campinaranas) within the Amazon rainforest biome of South America (Almeida et al. 2020; POWO 2024). For an identification key for all species of *Blepharandra*, see Anderson (1981).

#### ﻿1.2.2. Byrsonima

Taxon classificationPlantaeMalpighialesMalpighiaceae

Rich. ex Kunth, Nov. Gen. Sp. (quarto ed.) 5: 147. 1821 [1822].

BCD8BA26-B878-50A9-A42B-C2D209A953AE

Figs 3F, L , 6A , 7O , 8C, J , 9K , 10E–G

= Alcoceratothrix Nied., Arbeiten Bot. Inst. Königl. Lyceums Hosianum Braunsberg 1: 45. 1901. Type species: Alcoceratothrixrugosa (Benth.) Nied. (≡ Byrsonimarugosa Benth.).

##### Type species.

*Byrsonimaspicata* (Cav.) DC.

##### Notes.

*Byrsonima* currently comprises 164 accepted species (49 threatened species; Suppl. material 1) of trees, shrubs, or subshrubs endemic to most biomes of the Neotropical region from swamps in the State of Florida (USA) to rainforests, savannas, dry forests, and grasslands of Southern Brazil (Almeida et al. 2020; POWO 2024). Two subgenera are currently recognised in Byrsonima (subg. Byrsonima and subg. Macrozeugma Nied.), but neither is monophyletic (Francener 2016). There is no updated identification key for all species of *Byrsonima*, but for regional treatments, see Anderson (1981) for the Guyana Highland, Almeida et al. (2020) for Brazil, Pool (in prep.) for Mesoamerica, and Anderson (2016) for North America.

#### ﻿1.2.3. Diacidia

Taxon classificationPlantaeMalpighialesMalpighiaceae

Griseb. in Martius, Fl. Bras. 12(1): 119. 1858.

D6F306FE-36B2-5A7A-8865-EC1EEBB14B78

= Sipapoa Maguire, Mem. New York Bot. Gard. 8: 124. 1953. ≡ Diacidiasubg.Sipapoa (Maguire) W.R.Anderson, Mem. New York Bot. Gard. 32: 63. 1981. Type species: Sipapoakunhardtii Maguire [≡ Diacidiakunhardtii (Maguire) W.R.Anderson].

##### Type species.

*Diacidiagalphimioides* Griseb.

##### Notes.

*Diacidia* currently comprises 11 accepted species (seven threatened species; Suppl. material 1) of trees, shrubs or subshrubs endemic to campos rupestres and tepuis within the Amazon rainforest biome of South America (Almeida et al. 2020; POWO 2024). For an identification key for all species of *Diacidia*, see Anderson (1981) for the Guyana Highland and Almeida et al. (2020) for Brazil.

#### ﻿1.3. Galphimieae

Taxon classificationPlantaeMalpighialesMalpighiaceae

Nied. in Engler & Prantl, Nat. Pflanzenfam. III, 4: 53, 67. 1890.

E53621B6-4DDC-5825-89F6-CAFA15B24060

≡ Galphimiinae Nied. in Engler & Prantl, Nat. Pflanzenfam. III, 4: 53, 69. 1890.

##### Type genus.

*Galphimia* Cav.

##### Diagnosis.

Peduncle of cincinni present, floral buds with petals keeled, anther projections laterally inserted on thecae, presence of naphthopyrans and oxazinanes.

##### Notes.

Galphimieae currently comprises only three accepted genera (*Galphimia*, *Spachea*, and *Verrucularina*) and 40 species (20 threatened species; Suppl. material 1) of trees, shrubs or subshrubs endemic to the Americas (POWO 2024).

### ﻿Key to the genera of Galphimieae

**Table:** 

1	Trees; leaves as long as the inflorescences; inflorescences pendulous, bracteoles glandular, glands stalked; filaments not changing colour at post-anthesis	***Spachea***
–	Shrubs to subshrubs; leaves shorter than the inflorescences; inflorescences erect, bracteoles eglandular; filaments changing colour at post-anthesis	**3**
2	Leaves many-glandular; cincinni 1-flowered; calyx 0–5-glandular, when present secreting nectar, anthers smooth, unappendaged	***Galphimia* (Figs 7N, 8P, 10P)**
–	Leaves eglandular; cincinni 1–3-flowered; calyx 10-glandular, glands secreting oil, anthers with 2 verrucose appendages at apex	***Verrucularina* (Figs 3B, 4P, 10Q)**

#### ﻿1.3.1. Galphimia

Taxon classificationPlantaeMalpighialesMalpighiaceae

Cav., Icon. 5: 61–62, pl. 489. 1799 nom. cons.

A1D74E4A-F397-5774-897D-6C32A0B182BE

Figs 7N , 8P , 10P

= Thryallis L., Sp. Pl., ed. 2: 554. 1762, nom. rej. ≡ Vorstia Adans., Fam. Pl. 2: (23). 1763, nom. superfl. Type species: Thryallisbrasiliensis L. [≡ Galphimiabrasiliensis (L.) A.Juss.].

##### Type species.

*Galphimiaglauca* Cav.

##### Notes.

*Galphimia* currently comprises 26 accepted species (13 threatened species; Suppl. material 1) of shrubs to subshrubs endemic to the seasonally dry tropical forest biome in the Neotropics from the U.S.A. to Brazil (Anderson 2007; POWO 2024). For an identification key for all species of *Galphimia*, see Anderson (2007).

#### ﻿1.3.2. Spachea

Taxon classificationPlantaeMalpighialesMalpighiaceae

A.Juss. in Deless., Icon. Sel. Pl. 3: 19. 1838 [1837].

E062209A-9266-5246-B64F-06A827D15CC9

= Lophanthera A.Juss., Ann. Sci. Nat., Bot., sér. 2, 13: 328. 1840, syn nov. Type species: Lophantherakunthiana A.Juss., nom. superfl. [≡ Spachealongifolia (Kunth) R.F.Almeida & M.Pell.].
= Spacheasect.Meckelia Mart. ex A.Juss., Ann. Sci. Nat., Bot., sér. 2, 13: 326. 1840 ≡ Meckelia (Mart. ex A.Juss.) Griseb. in Martius, Fl. Bras. 12(1): 25. 1858. Type species: Spacheatricarpa A.Juss.
= Andersoniella C.Davis & Amorim, Harvard Pap. Bot. 25(1): 51–56. 2020, nom. illeg., non Andersoniella K.J.F.Schmitz (1897) ≡ Andersoniodoxa C.Davis & Amorim, Phytotaxa 470(1): 121–122. 2020, syn. nov. Type: Andersoniodoxaspruceana (Nied.) C.Davis & Amorim [≡ Lophantheraspruceana (Nied.) R.F.Almeida & M.Pell.].

##### Type.

*Spacheaelegans* (G.Mey.) A.Juss.

##### Notes.

*Spachea* was described by Jussieu (1837) to accommodate the species previously placed in *Byrsonima* with unisexual flowers. *Lophanthera* was initially described by Jussieu (1840) based on *L.kunthiana* A.Juss., an illegitimate renaming of *Galphimialongifolia* Kunth. Grisebach (1858) transferred *G.longifolia* to *Lophanthera* and placed *L.kunthiana* in synonymy. Niedenzu (1914) described the second species of *Lophanthera*, *L.spruceana* Nied., ca. 50 years after Grisebach. With the expansion of the Amazonian frontier in Brazil, Ducke described the third and fourth new species of the genus almost two decades later (1925, 1937). Finally, Davis et al. (2020a, b) proposed *Andersoniodoxa* for the three species of *Lophanthera* with white to pink flowers and winged anthers. This was, in theory, strongly supported by molecular data. Nonetheless, the authors never made the sequences used in their article available in public repositories, and the analysis produced by us includes the type species of the three genera and recovers them as a strongly supported clade. Thus, we propose the recognition of a broadly circumscribed but morphologically cohesive *Spachea*, including all species of *Lophanthera* and *Andersoniodoxa*.

In the expanded circumscription presented here, *Spachea* includes 12 species (five threatened species; Suppl. material 1) of large trees distributed in flooded to non-flooded rainforests from the Amazon basin and Central America (POWO 2024). The highly unusual structure of the fruits in *S.longifolia* and *S.spruceana* is worth mentioning, as it might be a water dispersal adaptation that enables buoyancy in the mericarp. For an identification key for *Spachea*, see Anderson (1981) for the Guyana Highland, Almeida et al. (2020) for Brazil, and Pool (in prep.) for Mesoamerica.

#### ﻿1.3.2.a. Spachea hammelii

Taxon classificationPlantaeMalpighialesMalpighiaceae

(W.R.Anderson) R.F.Almeida & M.Pell. comb. nov.

001FC556-25EC-5B76-8E6E-A7E90C465094

urn:lsid:ipni.org:names:77342383-1

≡ Lophantherahammelii W.R.Anderson, Brittonia 35: 37. 1983 ≡ Andersoniellahammelii (W.R.Anderson) C.Davis & Amorim, Harvard Pap. Bot. 25: 53. 2020 ≡ Andersoniodoxahammelii (W.R.Anderson) C.Davis & Amorim, Phytotaxa 470: 121. 2020.

#### ﻿1.3.2.b. Spachea lactescens

Taxon classificationPlantaeMalpighialesMalpighiaceae

(Ducke) R.F.Almeida & M.Pell. comb. nov.

BEDE6343-5DFC-5132-93F7-9A4ABB873E7D

urn:lsid:ipni.org:names:77342384-1

≡ Lophantheralactescens Ducke, Arch. Jard. Bot. Rio de Janeiro 4: 103. 1925.

#### ﻿1.3.2.c. Spachea longifolia

Taxon classificationPlantaeMalpighialesMalpighiaceae

(Kunth) R.F.Almeida & M.Pell. comb. nov.

46DB0099-2470-5BD8-AD58-7FA0BAA8A097

urn:lsid:ipni.org:names:77342385-1

≡ Galphimialongifolia Kunth in F.W.H. von Humboldt, A.J.A. Bonpland & C.S. Kunth, Nov. Gen. Sp. 5: 173. 1822 ≡ Lophantheralongifolia (Kunth) Griseb. in C.F.P.von Martius & auct. suc. (eds.), Fl. Bras. 12(1): 25. 1858.

#### ﻿1.3.2.d. Spachea marcelae

Taxon classificationPlantaeMalpighialesMalpighiaceae

(W.R.Anderson) R.F.Almeida & M.Pell. comb. nov.

C6275321-A488-50EF-8BF8-CFD068ED3289

urn:lsid:ipni.org:names:77342386-1

≡ Lophantheramarcelae W.R.Anderson, Acta Bot. Mex. 109: 37 (2014) ≡ Andersoniellamarcelae (W.R.Anderson) C.Davis & Amorim, Harvard Pap. Bot. 25: 53. 2020 ≡ Andersoniodoxamarcelae (W.R.Anderson) C.Davis & Amorim, Phytotaxa 470: 121. 2020.

#### ﻿1.3.2.e. Spachea pendula

Taxon classificationPlantaeMalpighialesMalpighiaceae

(Ducke) R.F.Almeida & M.Pell. comb. nov.

7837FD40-1BB9-5559-8541-E9C0CD7E5025

urn:lsid:ipni.org:names:77342387-1

≡ Lophantherapendula Ducke, Trop. Woods 50: 34. 1937.

#### ﻿1.3.2.f. Spachea spruceana

Taxon classificationPlantaeMalpighialesMalpighiaceae

(Nied.) R.F.Almeida & M.Pell. comb. nov.

48AB04A7-8477-5A5A-A282-5B462D8E0F53

urn:lsid:ipni.org:names:77342388-1

≡ Lophantheraspruceana Nied., Arbeiten Bot. Inst. Königl. Lyceums Hosianum Braunsberg 5: 30. 1914 ≡ Andersoniellaspruceana (Nied.) C.Davis & Amorim, Harvard Pap. Bot. 25: 55. 2020 ≡ Andersoniodoxaspruceana (Nied.) C.Davis & Amorim, Phytotaxa 470: 121. 2020.

#### ﻿1.3.3. Verrucularina

Taxon classificationPlantaeMalpighialesMalpighiaceae

Rauschert, Taxon 31(3): 560. 1982.

512A8077-7638-51B2-B33E-1DF937616C9D

≡ Verrucularia A.Juss., Ann. Sci. Nat., Bot., sér. 2, 13: 327. 1840, nom. illeg., non Verrucularia Shur. Figs 3B, 4P, 10Q.

##### Type species.

*Verrucularinaglaucophylla* (A.Juss.) Rauschert (≡ *Verruculariaglaucophylla* A.Juss.).

##### Notes.

*Verrucularina* is a replacement name for *Verrucularia* A.Juss. since the latter is a posterior homonym of *Verrucularia* Suhr, a genus previously assigned to algae but currently belonging to Bryozoa. The genus currently comprises two accepted species (one threatened species; Suppl. material 1) of shrubs endemic to campos rupestres of the Amazon rainforest and Caatinga biomes of Brazil, South America (Almeida et al. 2020; POWO 2024). For an identification key for all species of *Verrucularina*, see Anderson (1981) for the Guyana Highland or Almeida et al. (2020) for Brazil.

#### ﻿2. Malpighioideae

Taxon classificationPlantaeMalpighialesMalpighiaceae

Burnett, Outlines Bot.: 894, 1093, 1126. 1835, emend. nov. R.F.Almeida.

973759A2-9734-5488-BB93-FB4EF0881C03

##### Type genus.

*Malpighia* L.

##### Diagnosis.

Posterior petal glandular, 2–10 fertile stamens, pollen 3–12-aperturate, zono- to pantoaperturate, porate or colporate, styles capitate, uncinate, truncate, expanded or rarely subulate, stigmas usually lateral, nuts or mericarps, frequently winged or setose, rarely smooth, chromosome number *n* = 9–10, presence of dithiols, furanoid lignans, organic phosphoric acids and derivatives, and propargyl-type 1,3-dipolar organic compounds.

##### Notes.

Aside from subfamily Byrsonimoideae, all previously proposed subfamilies are recovered nested within Malpighioideae, making it non-monophyletic. Furthermore, most of these subfamilies are non-monophyletic on their own since they were traditionally circumscribed based on fruit morphology (especially dry vs. fleshy) and the presence or absence of mericarp wings. Therefore, in our current circumscription, subfamily Malpighioideae comprises nine main lineages of mostly Neotropical genera of Malpighiaceae (including *Burdachia*, *Glandonia*, and *Mcvaughia*, which were previously placed by Anderson 1977 in Byrsonimoideae). In its new circumscription, Malpighioideae comprises most of the family’s diversity (i.e., 63 genera and 1,254 species, with 624 threatened species; Suppl. material 1), including lianas, subshrubs, shrubs and trees occurring in the Americas, Africa, Asia, and Oceania. We recognise nine tribes representing the main lineages within Malpighioideae, previously named by Davis and Anderson (2010) and Almeida et al. (2023a) as: 1. Acridocarpoid clade (Acridocarpeae), 2. Mcvaughioid clade (Mcvaughieae), 3. Barnebyoid clade (Barnebyeae), 4. Ptilochaetoid clade (Ptilochaeteae), 5. Bunchosioid clade (Bunchosieae), 6. Hiraeoid clade (Hiraeeae), 7. Tetrapteroid clade (Hiptageae), 8. Stigmaphylloid clade (Gaudichaudieae), and 9. Malpighioid clade (Malpighieae).

### ﻿Key to the tribes of Malpighioideae

**Table:** 

1	Stipules absent; leaves alternate (Fig. 3D) to subopposite; bracts usually 1–2-glandular; styles lyrate (Fig. 9P), deflexed in flower, reflexed in fruit	**Acridocarpeae**
–	Stipules present; leaves opposite (Fig. 3A); bracts always eglandular (Fig. 7G–I), styles curved to straight (Fig. 9L, M), always erect	**2**
2	Cincinni 2–7-flowered (Fig. 5A)	**3**
–	Cincinni 1-flowered (Fig. 5A–C)	**4**
3	Shrubs or subshrubs; leaves distributed along the branches (Fig. 3A, B); bracteoles glandular (Fig. 7B, C); posterior petal glandular; drupes (Fig. 10M, N)	**Mcvaughieae**
–	Trees; leaves congested at the apex of the branches (Fig. 3D); bracteoles eglandular (Fig. 7G–I); posterior petal eglandular; winged schizocarp (Fig. 11)	**Barnebyeae**
4	Leaves margin revolute when young (Fig. 4P); thyrse main axis inconspicuous (Fig. 5A), usually with 4 cincinni	**Ptilochaeteae**
–	Leaves margin plane when young (Fig. 4O); thyrse main axis well-developed (Fig. 5A, C), with more than 4 cincinni	**5**
5	Stipules inconspicuous (Fig. 3F); petals limb abaxially densely pubescent or claws pubescent	**Hiptageae**
–	Stipules conspicuous (Fig. 3H); petals limb abaxially usually glabrous or claws glabrescent	**6**
6	Stipules epipetiolar (Fig. 3G)	**7**
–	Stipules interpetiolar (Fig. 3E, H)	**8**
7	Leaf apex eglandular, tertiary veins reticulate; styles straight, parallel, apex capitate, stigma terminal (Fig. 9L); mericarps smooth (Fig. 10O, S), setose (Fig. 10W), drupaceous (Fig. 10H, I), or 4-winged (X-shaped), wings coriaceous, non-reticulate (Fig. 11J–L)	**Bunchosieae**
–	Leaf apex glandular, tertiary veins scalariform; styles curved, divergent, apex uncinate, stigma lateral (Fig. 9I); mericarps 2-winged, wings membranous, finely reticulate (Fig. 11G)	**Hiraeeae**
8	Flowers arranged in umbels (thyrses in *Bronwenia*); fertile stamens 2–3–4–5–6–10; mericarps with a well-developed dorsal wing (larger than the lateral wings, when present), lateral wings reduced to absent (always smaller than the dorsal wing), free (Fig. 11A)	**Gaudichaudieae**
–	Flowers arranged in corymbs (thyrses in *Amorimia* and *Ectopopterys*); fertile stamens 10; drupes (*Malpighia*; Fig. 10J–L) or mericarps with a reduced dorsal wing (smaller than the lateral wings), lateral wings well-developed (larger than the dorsal wing), free (*Ectopopterys* and *Amorimia*; Fig. 11F) or fused into an orbicular wing (Fig. 11I)	**Malpighieae**

#### ﻿﻿2.1. Acridocarpeae

Taxon classificationPlantaeMalpighialesMalpighiaceae

R.F.Almeida, trib. nov.

B73101A0-6F7E-5D6A-89F7-28F377814653

urn:lsid:ipni.org:names:77342389-1

##### Type genus.

*Acridocarpus* Guill. & Perr.

##### Diagnosis.

Lianas, shrubs to treelets; thyrses, many-flowered, cincinni 1-flowered, bracts 1-glandular, peduncle absent, bracteoles eglandular; sepals glandular, nectariferous; posterior petals 2, margin crenate, eglandular; connectives eglandular, anthers poricidal, pollen 3-zonosyncolporate; styles reflexed in fruits; mericarps 1-winged, dorsal wing more developed, chromosome number *n* = 9, presence of diazanaphthalenes, isoflavonoids, oxacyclic compounds, absence of tetrahydrofurans.

##### Notes.

Acridocarpeae currently comprises only two accepted genera (*Acridocarpus* and *Brachylophon*) and 38 species (20 threatened species; Suppl. material 1) of trees, shrubs or lianas endemic to Africa, Asia, and Oceania (POWO 2024).

### ﻿Key to the genera of Acridocarpeae

**Table:** 

1	Leaf apex rounded, acute or mucronate; corolla rotate, petals patent, margin not entire; dorsal wing well-developed; Africa to Western Asia	***Acridocarpus***
–	Leaf apex caudate; corolla campanulate, petals erect, margin entire; dorsal wing very reduced; Southeastern Asia	***Brachylophon***

#### ﻿2.1.1. Acridocarpus

Taxon classificationPlantaeMalpighialesMalpighiaceae

Guill., Perr. & A.Rich., Fl. Seneg. Tent.: 123, t. 29. 1831.

C16FB563-8ADD-5A5A-B09B-5AC07A5D0A2D

= Heteropteryssect.Anomalopterys DC., Prodr. 1: 592. 1824 ≡ Anomalopterys (DC.) G.Don, Gen. Hist. 1: 647. 1831. Type species: Anomalopterysspicata G.Don [= Acridocarpussmeathmanii (DC.) Guill. & Perr.].
= Rhinopteryx Nied., Nat. Pflanzenfam. 3(4): 352. 1896. Type species: Rhinopteryxspectabilis Nied. [≡ Acridocarpusspectabilis (Nied.) Doorn-Hoekm.].

##### Type species.

*Acridocarpusplagiopterus* Guill., Perr. & A.Rich.

##### Notes.

*Acridocarpus* currently comprises 36 accepted species (19 threatened species; Suppl. material 1) of trees, shrubs, scandent shrubs, or lianas endemic to rainforests, savannas, and seasonally dry tropical forests of Africa, Madagascar, the Arabic Peninsula, Iran, and Oceania (i.e., New Caledonia; POWO 2024). There is no updated identification key for all species of *Acridocarpus*, but Niedenzu’s (1928) treatment covers 25 out of the 36 currently accepted species.

#### ﻿2.1.2. Brachylophon

Taxon classificationPlantaeMalpighialesMalpighiaceae

Oliv., Hooker’s Icon. Pl. 16: 1566. 1887.

5D1C344F-830E-5B40-8B2E-8A9CBB08022C

##### Type species.

*Brachylophoncurtisii* Oliv.

##### Notes.

*Brachylophon* currently comprises two accepted species (one threatened species; Suppl. material 1) of shrubs endemic to the rainforest biome in Southeast Asia (Indonesia, Malaysia, and Thailand; POWO 2024). For a taxonomic treatment for *Brachylophon*, see Sirirugsa (1991) for Thailand.

#### ﻿2.2. Mcvaughieae

Taxon classificationPlantaeMalpighialesMalpighiaceae

R.F.Almeida trib. nov.

BD7E2AAE-38AA-5491-BEFB-3808BF720754

urn:lsid:ipni.org:names:77342390-1

##### Type genus.

*Mcvaughia* W.R.Anderson.

##### Diagnosis.

Trees, shrubs to subshrubs; thyrses, cincinni 1–7-flowered, bracteoles 1-glandular; pollen 4-zonocolporate (3-zonocolporate in *Glandonia*); drupes, epicarp striated, presence of linear 1,3-diarylpropanoids, and the absence of dithiols, indoles and derivatives.

##### Notes.

Mcvaughieae currently comprises three accepted genera, *Burdachia*, *Glandonia*, and *Mcvaughia*, and 12 species (five threatened species; Suppl. material 1) of trees, shrubs to subshrubs endemic to the Amazon rainforests and seasonally dry tropical forests of South America (POWO 2024).

### ﻿Key to the genera of Mcvaughieae

**Table:** 

1	Pedicel straight at pre-anthesis, lateral petals yellow, fertile stamens 7, staminodes 3, anthers horseshoe-shaped, ovary 1-locular, styles straight at apex, stigma lateral; fruit pubescent	***Mcvaughia* (Figs 8G, 10M)**
–	Pedicel circinate at pre-anthesis, lateral petals pink or white, fertile stamens 10, staminodes absent, anthers straight, ovary 3-locular, styles bent at apex, stigma terminal; fruit glabrous.	**2**
2	Stipules connate in epipetiolar pairs, persistent; inflorescences deflexed; floral buds globose, lateral petals pink, filaments glabrous, connective expanded, locule apex rounded, shorter than the connective	***Burdachia* (Fig. 10N)**
–	Stipules connate in interpetiolar pairs, deciduous; inflorescences erect; floral buds pyramidal, lateral petals white, filaments pubescent, connective inconspicuous, locule apex acute, longer than the connective	***Glandonia***

#### ﻿2.2.1. Burdachia

Taxon classificationPlantaeMalpighialesMalpighiaceae

A.Juss. ex Endl., Gen. Pl.: 1064. 1840.

B76755BF-66CC-57A4-8BAE-F9D696CB9D90

Fig. 10N

= Tetrapodenia Gleason, Bull. Torrey Bot. Club 53: 289. 1926. Type species: Tetrapodeniaglandifera Gleason (= Burdachiasphaerocarpa A.Juss.).

##### Type species.

*Burdachiaprismatocarpa* A.Juss.

##### Notes.

*Burdachia* comprises only six currently accepted species (one threatened species; Suppl. material 1) of trees or shrubs endemic to flooded forests of the Amazon rainforests of Brazil, Colombia, Guyana, Peru, and Venezuela, South America (POWO 2024). For an identification key for all species of *Burdachia*, see Almeida et al. (2020) for Brazil or Anderson (1981) for the Guyana Highland.

#### ﻿2.2.1.a. Burdachia glandifera

Taxon classificationPlantaeMalpighialesMalpighiaceae

(Gleason) R.F.Almeida & M.Pell. comb. nov.

9B403B7E-F2A9-5A1C-AFB3-39622ED6FB3A

urn:lsid:ipni.org:names:77342391-1

≡ Tetrapodeniaglandifera Gleason, Bull. Torrey Bot. Club 53: 289. 1926 ≡ Burdachiasphaerocarpavar.glandifera (Gleason) W.R.Anderson, Mem. New York Bot. Gard. 32: 139. 1981.

#### ﻿2.2.1.b. Burdachia loretoensis

Taxon classificationPlantaeMalpighialesMalpighiaceae

(W.R.Anderson) R.F.Almeida & M.Pell. stat. nov.

C4C40D24-FCB6-5C1F-AA63-058F4B3887AB

urn:lsid:ipni.org:names:77342392-1

≡ Burdachiaprismatocarpavar.loretoensis W.R.Anderson, Mem. New York Bot. Gard. 32: 143. 1981.

#### ﻿2.2.2. Glandonia

Taxon classificationPlantaeMalpighialesMalpighiaceae

Griseb. in Martius, Fl. Bras. 12(1): 23. 1858.

1D1FE5BE-2B7F-5100-BD83-250366140FFF

##### Type species.

*Glandoniamacrocarpa* Griseb.

##### Notes.

*Glandonia* comprises only three currently accepted species (one threatened species; Suppl. material 1) of trees or shrubs endemic to flooded forests of the Amazon rainforests of Brazil, Colombia, and Venezuela, South America (POWO 2024). For an identification key for all species of *Glandonia*, see Almeida et al. (2020) for Brazil, Anderson (1981) for the Guyana Highland, or Guesdon et al. (2018) for the Brazilian Amazon.

#### ﻿2.2.3. Mcvaughia

Taxon classificationPlantaeMalpighialesMalpighiaceae

W.R.Anderson, Taxon 28: 157. 1979.

9F943E0B-C26F-5C1A-8894-20D14CAD6FC2

Figs 8G , 10M

##### Type species.

*Mcvaughiabahiana* W.R.Anderson.

##### Notes.

*Mcvaughia* comprises only three currently accepted species (all threatened species; Suppl. material 1) of shrubs endemic to the seasonally dry tropical forests of Northeastern Brazil, South America (i.e., Caatinga biome; POWO 2024). For an identification key for all species of *Mcvaughia*, see the taxonomic treatment of Almeida et al. (2019).

#### ﻿2.3. Barnebyeae

Taxon classificationPlantaeMalpighialesMalpighiaceae

R.F.Almeida trib. nov.

BDC7F2C7-9729-567A-8562-AF2D04D97A63

urn:lsid:ipni.org:names:77342393-1

##### Type genus.

*Barnebya* W.R.Anderson & B.Gates.

##### Diagnosis.

Trees; thyrses, cincinni 2–3-flowered; pollen 4-zonoporate; mericarps 1-winged, dorsal wing more developed, presence of diarylheptanoids, keto acids and derivatives, oxazinanes, absence of benzopyrans, furanoid lignans, glycerophospholipids, lignan glycosides, naphthalenes, naphthopyrans, propargyl-type 1,3-dipolar organic compounds, pteridines and derivatives, tetrahydrofurans.

##### Notes.

Barnebyeae currently comprises a single genus, *Barnebya*, and two accepted species (one threatened species; Suppl. material 1) of trees endemic to Brazil, South America (POWO 2024).

#### ﻿2.3.1. Barnebya

Taxon classificationPlantaeMalpighialesMalpighiaceae

W.R.Anderson & B.Gates, Brittonia 33(3): 275. 1981.

8E5000C9-DB83-59EE-A3ED-FA61CD8D713A

##### Type species.

*Barnebyadispar* (Griseb.) W.R.Anderson & B.Gates.

##### Notes.

*Barnebya* comprises two currently accepted species (one threatened species; Suppl. material 1) of large trees endemic to non-flooded forests of the Atlantic rainforest and Caatinga biomes in Brazil, South America (Almeida et al. 2020; POWO 2024). For an identification key for *Barnebya*, see Almeida et al. (2020).

#### ﻿2.4. Ptilochaeteae

Taxon classificationPlantaeMalpighialesMalpighiaceae

R.F.Almeida trib. nov.

3B5A3244-BD08-5D13-8214-DB9ABBACBC8D

urn:lsid:ipni.org:names:77342394-1

##### Type genus.

*Ptilochaeta* Turcz.

##### Diagnosis.

Treelets to shrubs; thyrses reduced, 4-flowered; pollen 8-zonocolporate, styles apex geniculate to truncate; mericarp winged or setose, presence of 2-aryl-benzofuran flavonoids, dibenzyl-butane lignans, isoflavonoids, oxacyclic compounds, oxanes, pyrrolidines, thiocarbonyl compounds, absence of organothiophosphorus compounds, thiophenes.

##### Notes.

Ptilochaeteae currently comprises three accepted genera, *Dinemandra*, *Lasiocarpus*, and *Ptilochaeta*, and ten currently accepted species (one threatened species; Suppl. material 1) of small trees or shrubs endemic to the Americas (POWO 2024).

### ﻿Key to the genera of Ptilochaeteae

**Table:** 

1	Sepals with stipitate glands, posterior petal glandular, fertile stamens 2 or 8; mericarps winged; arid and desert areas of Argentina and Chile	***Dinemandra***
–	Sepals eglandular, posterior petal eglandular, fertile stamens 10; mericarps setose; seasonally dry forests of Argentina, Bolivia, Brazil, Paraguay, and Mexico	**2**
2	Plants dioecious, sepal apex erect, petals narrowly elliptic, style apex expanded; Mexico	***Lasiocarpus* (Fig. 10U)**
–	Plants monoecious; sepal apex convolute, petals widely elliptic to obovate, style apex truncate; Argentina, Bolivia, Brazil, Paraguay	***Ptilochaeta* (Fig. 8O)**

#### ﻿2.4.1. Dinemandra

Taxon classificationPlantaeMalpighialesMalpighiaceae

A.Juss. ex Endl., Ann. Sci. Nat., Bot., sér. 2, 13: 255. 1840.

C30F47BF-138B-524B-90DA-014E52A3F15B

= Dinemagonum A.Juss., Arch. Mus. Hist. Nat. 3: 585. 1843, syn. nov. Type species: Dinemagonumbridgesianum A.Juss. [= Dinemandragayana (A.Juss.) R.F.Almeida & M.Pell.].

##### Type species.

*Dinemandraericoides* A.Juss. ex Endl.

##### Notes.

*Dinemandra* and *Dinemagonum* were traditionally distinguished from each other based exclusively on their fruit morphology, with *Dinemandra* presenting dominant lateral wings and *Dinemagonum* presenting a dominant dorsal wing. Nonetheless, both genera are strongly supported as sister based on molecular data, being further morphologically supported by stalked sepal glands basally connate forming pairs (Simpson 1989) and 8-colporate and reticulate pollen (Lowrie 1982). Recognising them as distinct provides no phylogenetic information and unnecessarily inflates this already genus-rich family. Thus, we propose a broadly circumscribed *Dinemandra*, including *Dinemagonum*. In the current circumscription, *Dinemandra* comprises two currently accepted species (no threatened species; Suppl. material 1) of shrubs endemic to the semi-desert vegetation of Chile, South America (POWO 2024). For an identification key for all species of *Dinemandra*, see Simpson (2011).

#### ﻿2.4.1.a. Dinemandra gayana

Taxon classificationPlantaeMalpighialesMalpighiaceae

(A.Juss.) R.F.Almeida & M.Pell. comb. nov.

F4409644-295C-539D-AB47-AE3924FE4311

urn:lsid:ipni.org:names:77342395-1

≡ Dinemagonumgayanum A.Juss., Arch. Mus. Hist. Nat. 3: 585. 1843.

#### ﻿2.4.2. Lasiocarpus

Taxon classificationPlantaeMalpighialesMalpighiaceae

Liebm., Vidensk. Meddel. Dansk Naturhist. Foren. Kjøbenhavn 1853: 90. 1854.

B3B18449-5EB0-5E79-B216-60FA3CA1AB62

Fig. 10U

##### Type species.

*Lasiocarpussalicifolius* Liebm.

##### Notes.

*Lasiocarpus* comprises four currently accepted species (one threatened species; Suppl. material 1) of trees endemic to the seasonally dry tropical forests of Mexico, North America (POWO 2024). For an identification key for all species of *Lasiocarpus*, see Cardona-Cruz et al. (2021).

#### ﻿2.4.3. Ptilochaeta

Taxon classificationPlantaeMalpighialesMalpighiaceae

Turcz., Bull. Soc. Imp. Naturalistes Moscou 16: 52. 1843.

71600CCE-4B6B-54E4-A23E-9822386F043A

Fig. 8O

##### Type species.

*Ptilochaetabahiensis* Turcz.

##### Notes.

*Ptilochaeta* comprises only three currently accepted species (no threatened species; Suppl. material 1) of trees endemic to the seasonally dry tropical forests of Argentina, Bolivia, Brazil, and Paraguay, South America (POWO 2024). After carefully analysing all type specimens of this genus, *Ptilochaetadensiflora* Nied. is proposed here as a new synonym of *Ptilochaetanudipes* Griseb. An identification key for most species of *Ptilochaeta* can be found in Almeida et al. (2020) for Brazil.

#### ﻿2.5. Bunchosieae

Taxon classificationPlantaeMalpighialesMalpighiaceae

R.F.Almeida trib. nov.

8FCD9909-5BFC-57C3-9A16-45061DAC3B66

urn:lsid:ipni.org:names:77342396-1

= Thryallidinae Nied. in Engler & Prantl, Nat. Pflanzenfam. III, 4: 53, 67. 1890, syn. nov. Type genus: Thryallis Mart., nom. cons.

##### Type genus.

*Bunchosia* Rich. ex Kunth.

##### Diagnosis.

Trees, shrubs or lianas; thyrses, cincinni 1-flowered; pollen 4–12-pantoporate (colporate in *Echinopterys* and *Heladena*); styles free, rarely connate, parallel; stigma terminal, capitate; drupes or mericarps smooth or winged, presence of azolidines, benzodioxoles, organochlorides, quinolizines, absence of organic carbonic acids and derivatives, organic phosphoric acids and derivatives.

##### Notes.

Bunchosieae currently comprises five accepted genera, *Bunchosia*, *Echinopterys*, *Heladena*, *Thryallis*, and *Tristellateia*, and 122 species (68 threatened species; Suppl. material 1) of mostly American taxa, except for the Paleotropical (i.e., tropics of Africa, Asia, and Oceania) *Tristellateia* (POWO 2024).

### ﻿Key to the genera of Bunchosieae

**Table:** 

1	Lianas; leaves glandular at or along margin	**2**
–	Trees, shrubs or scandent shrubs; leaves eglandular or glandular at base	**3**
2	Floral buds smooth, sepals 2-glandular, glands pedunculate, petal margin fimbriate to denticulate, anthers rimose, styles 3; mericarps smooth or setose; Neotropics	***Heladena* (Figs 8D, 10O)**
–	Floral buds keeled, sepals eglandular, petal margin entire, anthers poricidal, styles 1; mericarps winged; Paleotropics	***Tristellateia***
3	Leaves eglandular; stamen filaments pubescent; mericarps setose	***Echinopterys* (Fig. 10W)**
–	Leaves glandular at base; stamen filaments glabrous; mericarps smooth or drupaceous	**4**
4	Trees or erect shrubs; inflorescence, flowers and fruits with malpighiaceous hairs, bracteoles glandular, not surrounding floral buds; sepals 2-glandular, erect at anthesis, anthers connivant; mericarps drupaceous	***Bunchosia* (Figs 8F, 10H)**
–	Scandent shrubs; inflorescence, flowers and fruits with stellate hairs, bracteoles eglandular, surrounding floral buds; sepals eglandular, deflexed at anthesis, anthers divergent; mericarps smooth	***Thryallis* (Figs 7J, P, 10S)**

#### ﻿2.5.1. Bunchosia

Taxon classificationPlantaeMalpighialesMalpighiaceae

Rich. ex Kunth, Nov. Gen. Sp. 5: 118. 1821.

43306047-9A28-5226-A23E-D04E88778CF8

Figs 8F , 10H

= Malacmaea Griseb., Linnaea 13: 248. 1839. Type species: Malacmaeafluminensis Griseb. [= Bunchosiamaritima (Vell.) J.F.Macbr.].

##### Type species.

*Bunchosiaodorata* (Jacq.) DC.

##### Notes.

*Bunchosia* comprises 93 currently accepted species (46 threatened species; Suppl. material 1) of trees or shrubs endemic to non-flooded rainforests and seasonally dry tropical forest biomes in the Neotropics from Mexico to Argentina (POWO 2024; Suppl. material 1). There is no updated identification key for all species of *Bunchosia*, but for regional treatments, see Anderson (1981) for the Guyana Highland, Almeida et al. (2020) for Brazil, Pool (in prep.) for Mesoamerica, González-Gutiérrez and Meyer (2019) for the Antilles, and Anderson (2016) for North America.

#### ﻿2.5.2. Echinopterys

Taxon classificationPlantaeMalpighialesMalpighiaceae

A.Juss., Arch. Mus. Hist. Nat. 3: 342. 1843.

A805192F-1316-5DE6-BE84-55D96B6DD16C

Fig. 10W

= Bunchosiasect.Coelostylis A.Juss., Ann. Sci. Nat., Bot., sér. 2, 13: 325. 1840 ≡ Coelostylis (A.Juss.) Kuntze, Revis. Gen. Pl. 1: 87. 1891, nom. illeg., non Coelostylis Torr. & A.Gray. Type species: Coelostylisglandulosa Kuntze [= Echinopteryseglandulosa (A.Juss.) Small].

##### Type species.

*Echinopteryslappula* A.Juss. [= *Echinopteryseglandulosa* (A.Juss.) Small].

##### Notes.

*Echinopterys* comprises only two currently accepted species of shrubs or lianas endemic to the seasonally dry tropical forests of Mexico (POWO 2024). For an identification key for all species of *Echinopterys*, see Pool (in prep.).

#### ﻿2.5.3. Heladena

Taxon classificationPlantaeMalpighialesMalpighiaceae

A.Juss., Ann. Sci. Nat., Bot., sér. 2, 13: 321. 1840.

6CF5A48F-889F-5BAF-8495-D62DFE5FCECA

Figs 8D , 10O

= Henlea Griseb., Abh. Königl. Ges. Wiss. Göttingen 9: 37. 1860, syn. nov., nom. illeg., non Henlea H.Karst. ≡ Henleophytum H.Karst., Fl. Columb. 1: 158. 1861. Type species: Henleophytumechinatum (Griseb.) Small [≡ Heladenaechinata (Griseb.) R.F.Almeida & M.Pell.].
= Malpigiantha Rojas Acosta, Cat. Hist. Nat. Corrientes: 55. 1897. Type species: Malpigianthavolubilis Rojas Acosta [= Heladenamultiflora (Hook. & Arn.) Nied.].

##### Type species.

*Heladenamultiflora* (Hook. & Arn.) Nied.

##### Notes.

Similar to *Dinemandra* and *Dinemagonum*, *Heladena* and *Henleophytum* are strongly supported as sister by molecular data, being exclusively distinguished by their fruit morphology (*Heladena* having smooth mericarps and *Henleophytum* having setose mericarps). However, both genera share unique stalked peltate sepal glands, added to hairy petals, weakly coherent but soon separating styles, and stigmas elliptic and geniculate. Thus, we also propose the expansion of *Heladena* to include two currently accepted species (one threatened species; Suppl. material 1) of lianas endemic to the seasonally dry tropical forests of Cuba, Antilles, Central America, and South America (Argentina, Brazil, Paraguay and Uruguay) (POWO 2024).

#### ﻿2.5.3.a. Heladena echinata

Taxon classificationPlantaeMalpighialesMalpighiaceae

(Griseb.) R.F.Almeida & M.Pell. comb. nov.

36C10AF5-FF1A-5CF1-A067-00F286A3CF86

urn:lsid:ipni.org:names:77342397-1

≡ Henleaechinata Griseb., Abh. Königl. Ges. Wiss. Göttingen 9: 37. 1860 ≡ Henleophytumechinatum (Griseb.) Small in Britton & al., N. Amer. Fl. 25: 149. 1910.

#### ﻿2.5.4. Thryallis

Taxon classificationPlantaeMalpighialesMalpighiaceae

Mart., Nov. Gen. Sp. Pl. 3: 77. 1829 nom. cons.

940A1A8E-1FFE-5EC9-A2CE-8C687540D458

Figs 7J, P , 10S

≡ Hemsleyna Kuntze, Revis. Gen. Pl. 1: 88. 1891.

##### Type species.

*Thryallislongifolia* Mart.

##### Notes.

*Thryallis* comprises five currently accepted species (one threatened species; Suppl. material 1) of shrubs or lianas endemic to the rainforests, savannas, and seasonally dry tropical forests of Bolivia, Brazil, and Paraguay, South America (POWO 2024). For an identification key for all species of *Thryallis*, see Anderson (1995).

#### ﻿2.5.5. Tristellateia

Taxon classificationPlantaeMalpighialesMalpighiaceae

Thouars, Madagasc.: 14. 1806.

A78CB0C4-1F33-59D6-AA5F-A151ABBF8538

= Zymum Noronha ex Thouars, Hist. Vég. Îsles Austral. Afriq.: 69. 1808. Type species: Zymummadagascariense Spreng. (= Tristellateiamadagascariensis Poir.).
= Platynema Wight & Arn., Edinburgh New Philos. J. 15: 179. 1833. Type species: Platynemalaurifolium Wight & Arn. (= Tristellateiaaustralasiae A.Rich.).

##### Type species.

*Tristellateiamadagascariensis* Poir.

##### Notes.

*Tristellateia* comprises 21 species of lianas endemic to rainforests and seasonally dry tropical forests of Madagascar (19 threatened species; Suppl. material 1), with a single species occurring in continental Africa (Comoros, Kenya, Mozambique, Somalia, Tanzania) and another species endemic to Southeast Asia (Cambodia, Myanmar, Thailand, Malaysia, Philippines, Taiwan, and Vietnam), and Oceania (Australia, Bismarck Archipelago, Caroline Islands, Jawa, Lesser Sunda Islands, Maluku, Marianas, Nansei-shoto, New Caledonia, New Guinea, and Vanuatu; POWO 2024). For an identification key for all species of *Tristellateia*, see Arènes (1947).

#### ﻿2.6. Hiraeeae

Taxon classificationPlantaeMalpighialesMalpighiaceae

A.Juss., Ann. Sci. Nat., Bot., sér. 2, 13: 255. 1840, as “Hireae”, emend. nov. R.F.Almeida.

9B9FE46A-5AEA-535E-80C3-2EFFDFE6E499

##### Type genus.

*Hiraea* Jacq.

##### Diagnosis.

Lianas; leaf blades with apex glandular; thyrses, many-flowered; pollen 4–12-pantocolporate (porate in *Psychopterys*); styles with apex uncinate, stigma lateral; mericarps winged, 2 lateral wings more developed than the dorsal, usually butterfly-shaped, presence of piperidines, absence of benzofurans, benzopyrans, dithiols, furanoid lignans, hydroxy acids and derivatives, naphthopyrans, pteridines and derivatives, pyrimidine nucleosides.

##### Notes.

Hiraeeae currently comprises five accepted genera, *Adelphia*, *Excentradenia*, *Hiraea*, *Lophopterys* and *Psychopterys*, and 105 species (54 threatened species; Suppl. material 1) of lianas or shrubs endemic to the Americas (POWO 2024).

### ﻿Key to the genera of Hiraeeae

**Table:** 

1	Flowers arranged in thyrses	**2**
–	Flowers arranged in umbels	**4**
2	Inflorescence branches longitudinally costate; sepals 1-glandular, posterior petal shorter than laterals; mericarps with 2 V-shaped lateral wings	***Lophopterys* (Fig. 11G)**
–	Inflorescence branches smooth; sepals 2-glandular, posterior petal equalling or longer than laterals; mericarps with 2 butterfly-shaped lateral wings	**3**
3	Leaves apex glandular; bracteoles glandular; petals yellow, deflexed, margin fimbriate, posterior petal longer than laterals, styles apex uncinate	***Adelphia***
–	Leaves apex eglandular; bracteoles eglandular; petals white, patent, margin dentate to erose, posterior petal equalling laterals, styles apex capitate	***Psychopterys***
4	Stipules at base of petioles; posterior petal with fimbriae two times longer than those from the laterals; mericarps with lateral wings connate at base	***Excentradenia***
–	Stipules at middle or apex of petioles; all petals with equally long fimbriae; mericarps with lateral wings free	***Hiraea* (Figs 3G, 4S)**

#### ﻿2.6.1. Adelphia

Taxon classificationPlantaeMalpighialesMalpighiaceae

W.R.Anderson, Novon 16(2): 170–171. 2006.

7AEBA0A2-BB90-5B7C-9EF9-1A9824C64637

##### Type species.

*Adelphiahiraea* (Gaertn.) W.R.Anderson.

##### Notes.

*Adelphia* comprises four currently accepted species (two threatened species; Suppl. material 1) of lianas endemic to non-flooded rainforests of Central America and the Amazon basin, South America (POWO 2024). For an identification key for all species of *Adelphia*, see Anderson (2006).

#### ﻿2.6.2. Excentradenia

Taxon classificationPlantaeMalpighialesMalpighiaceae

W.R.Anderson, Contr. Univ. Michigan Herb. 21: 29. 1997.

30465AF2-B70D-5F15-8F23-717C7280FE74

##### Type species.

*Excentradeniaadenophora* (Sandw.) W.R.Anderson.

##### Notes.

*Excentradenia* comprises four currently accepted species (two threatened species; Suppl. material 1) of lianas endemic to non-flooded forests of the Amazon rainforests of Bolivia, Brazil, Guyana, French Guyana, Suriname, and Venezuela, South America (POWO 2024). For an identification key for all species of *Excentradenia*, see Anderson (1997).

#### ﻿2.6.3. Hiraea

Taxon classificationPlantaeMalpighialesMalpighiaceae

Jacq., Enum. Syst. Pl. 4. 1760.

8AA48B4B-5D5A-5D18-B7C7-795BDCCAAB95

Figs 3G , 4S

##### Type species.

*Hiraeareclinata* Jacq.

##### Notes.

*Hiraea* comprises 81 currently accepted species (43 threatened species; Suppl. material 1) of scandent shrubs or lianas endemic to rainforests from Mexico (North America) to Argentina (South America) but absent in the Antilles (POWO 2024). There is no updated identification key for all species of *Hiraea*, but for regional treatments, see Anderson (1981) for the Guyana Highland, Almeida et al. (2020) for Brazil, and Pool (in prep.) for Mesoamerica.

#### ﻿2.6.4. Lophopterys

Taxon classificationPlantaeMalpighialesMalpighiaceae

A.Juss. in Deless., Icon. Sel. Pl. 3: 18. 1838 [1837].

F09F660A-EF72-5B6C-B25E-D230CB16B677

Fig. 11G

= Dolichopterys Kosterm., Recueil Trav. Bot. Néerl. 32: 279. 1935. Type species: Dolichopteryssurinamensis Kosterm. [≡ Lophopteryssurinamensis (Kosterm.) Sandwith].

##### Type species.

*Lophopteryssplendens* A.Juss.

##### Notes.

*Lophopterys* currently comprises seven accepted species (two threatened species; Suppl. material 1) of lianas endemic to the non-flooded forests of the Amazon and Atlantic rainforests of Bolivia, Brazil, Guyana, French Guyana, Peru, Suriname, and Venezuela, South America (POWO 2024). For an identification key for all species of *Lophopterys*, see Anderson and Davis (2001).

#### ﻿2.6.5. Psychopterys

Taxon classificationPlantaeMalpighialesMalpighiaceae

W.R.Anderson & S.Corso, Contr. Univ. Michigan Herb. 25: 116. 2007.

7BCB33A2-A1BC-5FE2-9395-38BC679D1133

##### Type species.

*Psychopterysdipholiphylla* (Small) W.R.Anderson & S.Corso.

##### Notes.

*Psychopterys* comprises nine currently accepted species (five threatened species; Suppl. material 1) of lianas endemic to seasonally dry tropical forests of Belize, Guatemala, Honduras, Mexico, and Nicaragua, Central and North America (Pool 2023; POWO 2024). For an identification key for all species of *Psychopterys*, see Anderson and Corso (2007) and Pool (2023).

#### ﻿2.7. Hiptageae

Taxon classificationPlantaeMalpighialesMalpighiaceae

DC., Prodr. 1: 583. 1824, emend. nov. R.F.Almeida.

15B8EC64-BE67-5A3F-AEA4-7106A1FE3EE3

= Banisterieae DC., Prodr. 1: 584. 1824, syn. nov. ≡ Banisteriinae Nied. in Engler & Prantl, Nat. Pflanzenfam. III, 4: 52, 60. 1890. Type genus: Banisteria L., nom. rej. (= Heteropterys Kunth).
= Tricomarieae Nied. in Engler & Prantl, Nat. Pflanzenfam. III, 4: 52, 66. 1890, syn. nov. Type genus: Tricomaria Gillies ex Hook. & Arn.

##### Type genus.

*Hiptage* Gaertn.

##### Diagnosis.

Treelets, shrubs or lianas; thyrses, multi-flowered; pollen 4–12-pantocolporate (porate in *Hiptage* and some *Heteropterys*); nuts or mericarps winged, with 2–4-wings, butterfly, Y or X-shaped, rarely setose, absence of organic phosphonic acids and derivatives.

##### Notes.

Hiptageae currently comprises 17 accepted genera, *Alicia*, *Callaeum*, *Carolus*, *Chlorohiptage*, *Christianella*, *Dicella*, *Flabellaria*, *Flabellariopsis*, *Glicophyllum*, *Heteropterys*, *Hiptage*, *Jubelina*, *Malpighiodes*, *Mezia*, *Niedenzuella*, *Tetrapterys*, *Tricomaria*, and 377 species (163 threatened species; Suppl. material 1) occurring in the Americas, Africa, Asia and Oceania (POWO 2024).

### ﻿Key to the genera of Hiptageae (modified from Almeida and van den Berg 2021)

**Table:** 

1	Styles 1–2; mericarps with 3 free lateral wings or setose	**2**
–	Styles 3; mericarps with 1–2–4 free lateral wings	**3**
2	Style 1, apex truncate, stigma terminal; mericarps with 3 free lateral wings; Africa and Asia	***Hiptage* (Figs 7K, 8A, 11M)**
–	Styles 2, apex uncinate, stigma lateral; mericarps setose; South America (Argentina)	***Tricomaria* (Fig. 10T)**
3	Petals green, styles shorter than the filaments; mericarps with 1 lateral wing; Asia (Vietnam)	***Chlorohiptage***
–	Petals white, lilac, yellow, orange or red, styles longer than the filaments; mericarps with 2–4 free lateral wings; Africa or Americas	**4**
4	Sepals deflexed, stigma terminal; Africa	**5**
–	Sepals erect, stigma lateral; Americas	**6**
5	Leaves glandular at margin, petiole with 2–3 gland pairs; stigma capitate	***Flabellaria***
–	Leaves glandular near or along margin, petiole eglandular; stigma truncate	***Flabellariopsis***
6	Petals glabrous to glabrescent	**7**
–	Petals densely pubescent	**10**
7	Sepals not enclosing petals in bud, filaments usually glabrous	**8**
–	Sepals enclosing petals in bud, filaments usually pubescent	**9**
8	Flowers arranged in thyrses, corymbs or umbels, inflorescences never arranged in dichasia; mericarps with 1 dominant dorsal wing	***Heteropterys* (Figs 8L, Q, U, 11C, D)**
–	Flowers arranged in umbels, inflorescences arranged in dichasia; mericarps with 4 dominant lateral wings	***Tetrapterys* (Figs 4Q, 8T, 11J)**
9	Leaves glandular near or along margin, petioles eglandular; flowers arranged in umbels, 4-flowered, secondarily arranged in dichasia, bracteoles elliptic; mericarps with 2 dominant lateral wings, connate at base	***Malpighiodes***
–	Leaves glandular at margin, petioles usually with 1 gland pair; flowers arranged in thyrses, many-flowered, solitary, bracteoles triangular; mericarps with 2–4 dominant lateral wings, free	***Niedenzuella* (Figs 5B, C, 7E, 8N, 11L)**
10	Bracteoles leaf-like; sepals enlarged in fruit; nuts	***Dicella* (Figs 7H, I, 10B–D)**
–	Bracteoles minute; sepals not enlarged in fruit; schizocarps	**11**
11	Flowers arranged in umbels, 4-flowered, secondarily arranged in dichasia	**12**
–	Flowers arranged in thyrses, many-flowered, solitary or grouped but never secondarily arranged in dichasia	14
12	Sepals deflexed at anthesis, anterior lateral petals deflexed at anthesis, posterior lateral petals patent at anthesis; mericarps with several lateral winglets, vertically inserted between lateral wings and the dorsal wing	***Jubelina***
–	Sepals erect to patent at anthesis, lateral petals deflexed at anthesis; mericarps without lateral winglets, when present (in *Mezia*) horizontally inserted between the lateral wings and the dorsal wing	**13**
13	Bracteoles not enclosing floral bud; connectives inconspicuous; lateral wings free	***Callaeum***
–	Bracteoles enclosing floral bud; connectives expanded; lateral wings connate at base	***Mezia* (Fig. 7D)**
14	Bracts, bracteoles, sepals and petals glandular at margin; mericarps with acicular (unbranched) hairs	***Christianella* (Figs 7C, M, 8B)**
–	Bracts, bracteoles, sepals and petals eglandular at margin; mericarps with 2-branched hairs	**15**
15	Stipules interpetiolar; petioles eglandular; petal margin fimbriate, anthers pubescent	***Carolus* (Fig. 11H)**
–	Stipules epipetiolar; petioles glandular; petal margin glandular, erose or dentate, anthers glabrous	**16**
16	Petioles with 2–4 gland pairs; bracteoles eglandular; lateral petals erect; mericarps bearing 2 dominant lateral wings, usually connate at base	***Alicia* (Figs 7G, 8Y)**
–	Petioles eglandular or with 1 gland pair; bracteoles glandular; lateral petals patent; mericarps bearing 2–4 dominant lateral wings, free	***Glicophyllum* (Figs 7B, 11K)**

#### ﻿2.7.1. Alicia

Taxon classificationPlantaeMalpighialesMalpighiaceae

W.R.Anderson, Novon 16: 174. 2006.

46D16844-5560-52AA-8E58-E4E258B5041E

Figs 7G , 8Y

##### Type species.

*Aliciaanisopetala* (A.Juss.) W.R.Anderson.

##### Notes.

*Alicia* comprises only two currently accepted species (no threatened species; Suppl. material 1) of lianas endemic to rainforests and seasonally dry tropical forests of Argentina, Bolivia, Brazil, Colombia, Ecuador, Guyana, Paraguay, Peru, Suriname, and Venezuela, South America (POWO 2024). For an updated identification key for all species of *Alicia*, see Anderson (2006) or Almeida et al. (2020).

#### ﻿2.7.2. Callaeum

Taxon classificationPlantaeMalpighialesMalpighiaceae

Small in Britton & al., N. Amer. Fl. 25: 128. 1910.

5B283EBB-DE18-5DB9-9D47-247935FA97F1

= Cabi Ducke, Arq. Serv. Florest. 2(1): 13. 1943. Type species: Cabiparaensis Ducke [= Callaeumantifebrile (Griseb.) D.M.Johnson].

##### Type species.

*Callaeumnicaraguense* (Griseb.) Small.

##### Notes.

*Callaeum* comprises 11 currently accepted species (five threatened species; Suppl. material 1) of scandent shrubs or lianas endemic to rainforests, savannas, and seasonally dry tropical forests from the United States (North America) to Argentina (South America; POWO 2024). For an identification key for all species of *Callaeum*, see Johnson (1986).

#### ﻿2.7.3. Carolus

Taxon classificationPlantaeMalpighialesMalpighiaceae

W.R.Anderson, Novon 16: 186. 2006.

754EC731-FEFD-5DEA-A1E3-D011CC71D8F7

Fig. 11H

##### Type species.

*Caroluschlorocarpus* (A.Juss.) W.R.Anderson.

##### Notes.

*Carolus* comprises eight currently accepted species (five threatened species; Suppl. material 1) endemic to rainforests, savannas, and seasonally dry tropical forests from Mexico (North America) to Brazil (South America; POWO 2024). For an identification key for all species of *Carolus*, see the synopsis of Anderson (2006) for the entire genus, Almeida et al. (2023c) for Brazil, and Pool (2023) for Mesoamerica.

#### ﻿2.7.4. Chlorohiptage

Taxon classificationPlantaeMalpighialesMalpighiaceae

T.V.Do, T.A.Le & R.F.Almeida, Plant Ecol. Evol. 157(2): 130. 2024.

AEC2CE96-D672-5323-A477-E331CC4886A6

##### Type species.

*Chlorohiptagevietnamensis* T.V.Do, T.A.Le & R.F.Almeida.

##### Notes.

*Chlorohiptage* comprises a single species (one threatened; Suppl. material 1) of lianas endemic to rainforests of Vietnam, Southeast Asia (Do et al. 2024). For a taxonomic treatment of the new genus, see Do et al. (2024).

#### ﻿2.7.5. Christianella

Taxon classificationPlantaeMalpighialesMalpighiaceae

W.R.Anderson, Novon 16: 190. 2006.

45187F68-04C9-5809-A0DB-E727B04D3C40

Figs 7C, M , 8B

##### Type species.

*Christianellamesoamericana* (W.R.Anderson) W.R.Anderson.

##### Notes.

*Christianella* comprises five currently accepted species (two threatened species; Suppl. material 1) of lianas endemic to rainforests, savannas, and seasonally dry tropical forests from Mexico (North America) to Brazil (South America; POWO 2024). For an identification key for all species of *Christianella*, see Anderson (2006).

#### ﻿2.7.6. Dicella

Taxon classificationPlantaeMalpighialesMalpighiaceae

Griseb., Linnaea 13: 249. 1839.

67E12857-783F-5763-8D21-2510B685F2B8

Figs 7H, I , 10B–D

##### Type species.

*Dicellabracteosa* (A.Juss.) Griseb.

##### Notes.

*Dicella* comprises seven currently accepted species (one threatened species; Suppl. material 1) of lianas endemic to rainforests, savannas, and seasonally dry tropical forests from Costa Rica (Central America) to Argentina (South America; POWO 2024). For an identification key for all species of *Dicella*, see Chase (1981).

#### ﻿2.7.7. Flabellaria

Taxon classificationPlantaeMalpighialesMalpighiaceae

Cav., Diss. 9: 436. 1790.

000B4BEA-2201-51B0-9680-9C79B9D03CD4

##### Type species.

*Flabellariapaniculata* Cav.

##### Notes.

*Flabellaria* comprises a single currently accepted species of liana endemic to rainforests, savannas, and seasonally dry tropical forests of Africa (POWO 2024). For a taxonomic treatment for *Flabellaria*, see Wilczek (1958).

#### ﻿2.7.8. Flabellariopsis

Taxon classificationPlantaeMalpighialesMalpighiaceae

R.Wilczek, Bull. Jard. Bot. État 25: 303, pl. 8. 1955.

74AF65F2-277E-5984-A65C-7AD519E4267B

##### Type species.

*Flabellariopsisacuminata* (Engl.) R.Wilczek.

##### Notes.

*Flabellariopsis* comprises a single currently accepted species (not threatened; Suppl. material 1) of liana endemic to rainforests, savannas, and seasonally dry tropical forests of Cameroon, Congo, Gabon, Tanzania, Uganda, and Zaire, Africa (POWO 2024). For a taxonomic treatment for *Flabellariopsis*, see Wilczek (1958).

#### ﻿2.7.9. Glicophyllum

Taxon classificationPlantaeMalpighialesMalpighiaceae

R.F.Almeida, Nordic J. Bot. 39: 12. 2021.

47FF6EEF-EA6D-53EA-804B-0B714B79A94F

Figs 7B , 11K

##### Type species.

*Glicophyllumchamaecerasifolium* (A.Juss.) R.F.Almeida.

##### Notes.

*Glicophyllum* comprises 28 currently accepted species (four threatened species; Suppl. material 1) of shrubs, subshrubs or lianas endemic to rainforests, savannas, and seasonally dry tropical forests from Mexico (North America) to Argentina (South America; POWO 2024). There is no updated identification key for all species of *Glicophyllum*, but for regional treatments, see Anderson (1981) for the Guyana Highland (under *Tetrapterys*), Almeida et al. (2020) for Brazil, and Pool (in prep.) for Mesoamerica.

#### ﻿2.7.9.a. Glicophyllum argenteum

Taxon classificationPlantaeMalpighialesMalpighiaceae

(A.Juss.) R.F.Almeida & M.Pell. comb. nov.

61176D04-E8D3-53E5-9877-F389755C2D9C

urn:lsid:ipni.org:names:77342398-1

≡ Hiraeaargentea A.Juss., Fl. Bras. Merid. 3: 17. 1833 ≡ Tetrapterysjussieuana Nied. in Engler, Nat. Pflanzenr. 93: 169. 1928 ≡ Glicophyllumjussieuanum (Nied.) R.F.Almeida, Nordic J. Bot. 39(1)-e02876: 15. 2021.

#### ﻿2.7.10. Heteropterys

Taxon classificationPlantaeMalpighialesMalpighiaceae

Kunth, Nov. Gen. Sp. 5 [quarto]: 163. 1822 [1821] nom. cons.

A553EDE3-2633-53D1-B45F-3D42B151C659

Figs 8L, Q, U , 11C, D

= Banisteria L., Sp. Pl.: 427. 1753, nom. rej. Type species: Banisteriabrachiata L. [≡ Heteropterysbrachiata (L.) DC.].
= Banisteriasect.Holopetalon Griseb., Linnaea 13: 199. 1839 ≡ Holopetalon (Griseb.) Rchb., Deut. Bot. Herb.-Buch: 207. 1841. Type species: Banisteriapatens Griseb. [≡ Heteropteryspatens (Griseb.) A.Juss.]
= Clonodia Griseb. in Martius, Fl. Bras. 12(1): 26. 1858. Type species: Clonodiaverrucosa Griseb. (= Heteropterysracemosa A.Juss.).
= Atopocarpus Cuatrec., Webbia 13: 454. 1958. Type species: Atopocarpuspapillosus Cuatrec. (= Heteropterysracemosa A.Juss.).
= Skoliopteris Cuatrec., Webbia 13: 451. 1958. Type species: Skoliopterislehmanniana (Nied.) Cuatrec. [= Heteropteryscomplicata (Kunth) W.R.Anderson & C.Davis].

##### Type species.

*Heteropteryspurpurea* (L.) Kunth.

##### Notes.

*Heteropterys* comprises 166 currently accepted species (75 threatened species; Suppl. material 1) of treelets, shrubs, subshrubs or lianas endemic to rainforests, savannas, and seasonally dry tropical forests from North America (Mexico) to South America (Argentina), and West Africa (Angola, Cameroon, Congo, Gabon, Ghana, Guinea, Guinea-Bissau, Ivory Coast, Liberia, Senegal, Sierra Leone, and Zaire; POWO 2024). There is no updated identification key for all species of *Heteropterys*, but for regional treatments, see Anderson (1981) for the Guyana Highland, Almeida et al. (2020) for Brazil, and Pool (in prep.) for Mesoamerica. *Glicophyllumjussieuanum* (Nied.) R.F.Almeida is here placed in the synonymy of *G.argenteum* (A.Juss.) R.F.Almeida & M.Pell. *comb. nov.*, due to its basionym being a replacement name for *Hiraeaargentea* A.Juss.

#### ﻿2.7.11. Hiptage

Taxon classificationPlantaeMalpighialesMalpighiaceae

Gaertn., Fruct. Sem. Pl. 2: 169. 1790 nom. cons.

BB51D20B-8102-5389-83B5-5B69B42C69B0

Figs 7K , 8A , 11M

= Gaertnera Schreb., Gen. Pl., ed. 8[a]. 1: 290. 1789, nom. rej. Type species: Gaertneraindica J.F. Gmel. [= Hiptagebenghalensis (L.) Kurz].
= Molina Cav., Diss. 9: 435. 1790. Type species: Molinaracemosa Cav. [= Hiptagebenghalensis (L.) Kurz].
= Succowia Dennst., Schlüssel Hortus Malab.: 32. 1818, nom. illeg., non Succowia Medik. Type species: Succowiafimbriata Dennst. [= Hiptagebenghalensis (L.) Kurz].

##### Type species.

*Hiptagemadablota* Gaertn. [= *Hiptagebenghalensis* (L.) Kurz].

##### Notes.

*Hiptage* comprises 47 currently accepted species (39 threatened species; Suppl. material 1) of lianas endemic to rainforests and seasonally dry tropical forests of Southeast Asia (Bangladesh, Cambodia, China, India, Indonesia, Laos, Malaysia, Myanmar, Nepal, Pakistan, Philippines, Sri Lanka, Taiwan, Thailand, and Vietnam) and Oceania (Fiji; POWO 2024). There is no updated identification key for all species of *Hiptage*, but for regional treatments, see Srivastava (1997) for India, Sirirugsa (1991) for Thailand, Chen and Funston (2008) for China, and Lim (2017) for Malaysia.

#### ﻿2.7.12. Jubelina

Taxon classificationPlantaeMalpighialesMalpighiaceae

A.Juss. in Deless., Icon. Sel. Pl. 3: 19, pl. 32. 1838 [1837].

24C1DEE5-300B-5AF9-9527-51187A78C95B

= Sprucina Nied., Arbeiten Bot. Inst. Königl. Lyceums Hosianum Braunsberg 3: 18. 1908. Type species (designated here): J.grisebachiana W.R.Anderson.

##### Type species.

*Jubelinariparia* A.Juss.

##### Notes.

No names have ever been published under the generic name *Sprucina*, but the collection cited in the protologue (Spruce 2853) refers to *J.grisebachiana* W.R.Anderson. Thus, *J.grisebachiana* is here designated as the type of *Sprucina* under Art. 10.2 Ex. 2 (Turland et al. 2018). *Jubelina* comprises six currently accepted species (one threatened species; Suppl. material 1) of lianas endemic to the rainforests of Brazil, Colombia, and Venezuela, South America (POWO 2024). For an identification key for all species of *Jubelina*, see Anderson (1990).

#### ﻿2.7.13. Malpighiodes

Taxon classificationPlantaeMalpighialesMalpighiaceae

Nied., Verz. Vorles. Königl. Lyceum Hosianum Braunsberg 1909–1910: 31. 1909.

D3B179D6-DB44-5D68-893C-5AE436A42F70

##### Type species.

*Malpighiodesspruceana* Nied. [=*Malpighiodesbracteosa* (Griseb.) W.R.Anderson].

##### Notes.

*Malpighiodes* comprises four currently accepted species (one threatened species; Suppl. material 1) of lianas endemic to the rainforests of the Amazon basin of Brazil, French Guiana, Guyana, Suriname, and Venezuela, South America (POWO 2024). For an identification key for all species of *Malpighiodes*, see Anderson (2006).

#### ﻿2.7.14. Mezia

Taxon classificationPlantaeMalpighialesMalpighiaceae

Schwacke ex Nied. in Engler & Prantl, Nat. Pflanzenfam. III, 4: 58. 1890.

FBD8F819-CF4F-5B02-8C6D-5D48A7048670

Fig. 7D

= Stenocalyx Turcz., Bull. Soc. Imp. Naturalistes Moscou 31(1): 393. 1858, nom. illeg., non Stenocalyx O.Berg. (1856). Type species: Stenocalyxinvolutus Turcz. [= Meziaincludens (Benth.) Cuatrec.].

##### Type species.

*Meziaaraujoi* Schwacke ex Nied.

##### Notes.

*Mezia* comprises 15 currently accepted species (eight threatened species; Suppl. material 1) of lianas endemic to the rainforests of the Amazon and Atlantic rainforest biomes in South America (Bolivia, Brazil, Colombia, Ecuador, French Guiana, Guyana, Peru, Suriname, and Venezuela) and Panama, Central America (POWO 2024). For a taxonomic treatment for all species of *Mezia*, see Anderson and Anderson (2018).

#### ﻿2.7.15. Niedenzuella

Taxon classificationPlantaeMalpighialesMalpighiaceae

W.R.Anderson, Novon 16(2): 194–198. 2006.

99DA5546-92F9-5FB3-8160-1343AA966002

Figs 5B, C , 7E , 8N , 11L

= Aenigmatanthera W.R.Anderson, Novon 16: 173. 2006. Type species: Aenigmatantheralasiandra (A.Juss.) W.R.Anderson [≡ Niedenzuellalasiandra (A.Juss.) R.F.Almeida].

##### Type species.

*Niedenzuellapoeppigiana* (A.Juss.) W.R.Anderson.

##### Notes.

*Niedenzuella* currently comprises 18 accepted species (three threatened species; Suppl. material 1) of lianas endemic to rainforests, savannas, and seasonally dry tropical forests of South America (Argentina, Bolivia, Brazil, Colombia, Ecuador, French Guiana, Guyana, Paraguay, Peru, Suriname, and Venezuela) and Central America (Costa Rica and Panama; POWO 2024). For an identification key for all species of *Niedenzuella*, see Anderson (2006, also under *Aenigmatanthera*). *Aenigmatanthera* was reduced to a synonym of *Niedenzuella* by Almeida and van den Berg (2021) since its two species were recovered strongly supported as nested within the latter.

#### ﻿2.7.16. Tetrapterys

Taxon classificationPlantaeMalpighialesMalpighiaceae

Cav., Diss. 9: 433. 1790.

2E741D23-54F8-59A6-A604-BF8F251DEEB9

Figs 4Q , 8T , 11J

= Adenoporces Small in Britton & al., N. Amer. Fl. 25: 128. 1910. Type species: Adenoporcesbuxifolius (Cav.) Small (≡ Tetrapterysbuxifolia Cav.).

##### Type species.

*Tetrapterysinaequalis* Cav.

##### Notes.

*Tetrapterys* comprises 56 currently accepted species (18 threatened species; Suppl. material 1) of shrubs and lianas endemic to rainforests, savannas, and seasonally dry tropical forests of the Neotropics from Mexico (North America to Argentina (South America; POWO 2024). There is no updated identification key for all species of *Tetrapterys*, but for regional treatments, see Anderson (1981) for the Guyana Highland, Almeida et al. (2020) for Brazil, and Pool (in prep.) for Mesoamerica.

#### ﻿2.7.16.a. Tetrapterys andina

Taxon classificationPlantaeMalpighialesMalpighiaceae

(Nied.) R.F.Almeida & M.Pell. stat. nov.

06A8CB2B-BB1F-5234-B788-D3F6FCDEBFC2

urn:lsid:ipni.org:names:77342399-1

≡ Tetrapterysdiscolorvar.andina Nied., Verz. Vorles. Königl. Lyceum Hosianum Braunsberg 1909–1910: 42. 1909.

#### ﻿2.7.17. Tricomaria

Taxon classificationPlantaeMalpighialesMalpighiaceae

Gillies ex Hook. & Arn., Bot. Misc. 3: 157. 1833.

E5DCC0BC-929C-5611-864B-ED9011BF9A80

##### Type species.

*Tricomariausillo* Gillies ex Hook. & Arn.

##### Notes.

*Tricomaria* comprises a single currently accepted species (no threatened species; Suppl. material 1) of shrubs endemic to the seasonally dry tropical forests of Argentina, South America (POWO 2024). For a taxonomic treatment of *Tricomaria*, see Aliscioni and Torretta (2017).

#### ﻿2.8. Malpighieae

Taxon classificationPlantaeMalpighialesMalpighiaceae

DC., Prodr. 1: 577. 1824, emend. nov. R.F.Almeida

1AA4A913-426C-5D9C-A779-98FDF1CBA67B

≡ Malpighiinae Nied. in Engler & Prantl, Nat. Pflanzenfam. III, 4: 53, 71. 1890.
= Aspidopteryinae Nied. in Engler & Prantl, Nat. Pflanzenfam. III, 4: 52, 53. 1890, as “Aspidopteridinae”, syn. nov. Type genus: Aspidopterys A.Juss. ex Endl.
= Mascagniinae Nied. in Engler & Prantl, Nat. Pflanzenfam. III, 4: 52, 55. 1890, syn. nov. Type genus: Mascagnia (Bertero ex DC.) Bertero.
= Rhynchophoreae Arènes, Notul. Syst. (Paris) 12: 135. 1946, syn. nov. Type genus: Rhynchophora Arènes.

##### Type genus.

*Malpighia* L.

##### Diagnosis.

Treelets, shrubs or lianas; thyrses or corymbs; pollen 4–12-pantocolporate (porate in the Paleotropical species); styles with apex uncinate to truncate, stigma lateral; mericarps winged, 1–2-wings, butterfly-shaped to orbicular, rarely drupaceous or with dorsal wing more developed than lateral ones, presence of (3’–>5’)-dinucleotides and analogues, piperidines, absence of benzofurans, furanoid lignans, imidolactams, lignan glycosides.

##### Notes.

Malpighieae currently comprises 13 accepted genera: *Amorimia*, *Aspidopterys*, *Calcicola*, *Caucanthus*, *Diaspis*, *Digoniopterys*, *Ectopopterys*, *Madagasikaria*, *Malpighia*, *Mascagnia*, *Microsteira*, *Rhynchophora*, and *Triaspis*, and 253 species (157 threatened species; Suppl. material 1) occurring in the Americas, Africa, and Asia (POWO 2024).

### ﻿Key to the genera of Malpighieae (modified from Almeida 2018)

**Table:** 

1	Plants androdioecious; flowers actinomorphic, sepals eglandular, stigmas terminal	**2**
–	Plants dioecious (androdioecious in *Triaspis*); flowers zygomorphic, sepals glandular, stigmas lateral (terminal in *Triaspis*)	**8**
2	Style apex truncate; continental Africa, Arabian Peninsula, and Asia	**3**
–	Style apex with projections (1–2-lobed); Madagascar	**5**
3	Flowers arranged in umbels; flower buds obovoid, petal margin entire, reflexed, filaments slightly longer than sepals; Asia	***Aspidopterys***
–	Flowers arranged in corymbs; flower buds ovoid to oblongoid, petal margin fimbriate to lobed, patent, filaments shorter or two times longer than sepals; Africa and Arabian Peninsula	**4**
4	Leaves spirally-alternate, glabrous; petal margin fimbriate, limb base obtuse, 2-carpellate	***Diaspis***
–	Leaves opposite, tomentose; petals margin undulate, limb base sagittate, 3-carpellate	***Caucanthus***
5	Leaves spirally-alternate, up to 5 mm wide; umbels 1-flowered; petals narrowly spatulate, abaxially completely densely sericeous, style apex long-lobed	***Digoniopterys***
–	Leaves opposite, at least 1 cm wide (mostly much wider); umbels 4–many-flowered; petals elliptic to orbicular, glabrous or abaxially sparsely sericeous along the keel, style apex shortly-lobed	**6**
6	Stipules enlarged, leaf-like, persistent; flowers in thyrses; mericarps with wings fused into an orbicular wing	***Madagasikaria***
–	Stipules reduced, triangular, persistent to deciduous; flowers in umbels; mericarps with lateral wings fused into a single apical geniculate wing or a Y-shaped wing	**7**
7	Ovary bearing conspicuous initials for lateral wings and dorsal crest on each carpel, visible even in young flowers; mericarps dehiscent, lateral wings fused into a Y-shaped wing	***Microsteira***
–	Ovary lacking initials for wings or crests; mericarps indehiscent, lateral wings fused into a single apical geniculate wing	***Rhynchophora***
8	Plants monoecious; stipules connate, leaf-like; bracteoles inserted at middle or below peduncle apex; floral buds keeled; sepals 1–2-glandular, glands very reduced, secreting nectar, petal margin long-fimbriate, limb base sagittate; Paleotropics	***Triaspis***
–	Plants dioecious; stipules free, triangular; bracteoles inserted at peduncle apex (except in *Calcicola*); floral buds smooth (except in few *Mascagnia* spp.); sepals 2-glandular, glands large, secreting oil, petal margin entire, limb base obtuse, cuneate or rounded; Neotropics	**9**
9	Sepals deflexed at anthesis, connectives bearing large glands, style apex lobed; mericarp with a dominant dorsal wing	***Ectopopterys***
–	Sepals erect at anthesis, connectives bearing inconspicuous glandular tissue, style apex truncate; mericarp with dominant lateral wings or wings greatly reduced and fleshy at maturity	**10**
10	Lianas; flowers arranged in thyrses or corymbs, bracteoles 1–6-glandular, rarely eglandular	**11**
–	Shrubs to treelets; flowers arranged in umbels, bracteoles eglandular	**12**
11	Flowers arranged in thyrses, bracteoles 2–6-glandular; floral buds smooth; petals yellow, turning orange to red at post-anthesis, pubescent; mericarp with lateral wings free, coriaceous	***Amorimia* (Figs 7A, 8M, 9I, J, 11F)**
–	Flowers in thyrses or corymbs, bracteoles 0–1-glandular; floral buds keeled; petals white, pink or lilac, if yellow not turning orange to red at post-anthesis; glabrous; mericarp with lateral wings fused into an orbicular wing, membranous	***Mascagnia* (Figs 3E, 6B, 8Z, 11I)**
12	Leaves with stiff, spine-like (generally urticating) hairs; bracteoles inserted at peduncle apex; ovary glabrous; mericarps indehiscent, fleshy, dorsal and lateral wings much reduced, free, fleshy at maturity	***Malpighia* (Fig. 10J–L)**
–	Leaves with soft hairs; bracteoles inserted at peduncle middle; ovary pubescent; mericarps dehiscent, dry, lateral wings conspicuous, fused	***Calcicola***

#### ﻿2.8.1. Amorimia

Taxon classificationPlantaeMalpighialesMalpighiaceae

W.R.Anderson, Novon 16: 176. 2006.

4F2C918B-8EE1-5745-AF10-CCE4A4F34438

Figs 7A , 8M , 9I, J , 11F

##### Type species.

*Amorimiarigida* (A.Juss.) W.R.Anderson.

##### Notes.

*Amorimia* comprises 15 currently accepted species (eight threatened species; Suppl. material 1) of lianas endemic to rainforests, savannas, and seasonally dry tropical forests of Argentina, Bolivia, Brazil, Colombia, Ecuador, Paraguay, and Peru, South America (POWO 2024). For a taxonomic treatment for all species of *Amorimia*, see Almeida (2018).

#### ﻿2.8.2. Aspidopterys

Taxon classificationPlantaeMalpighialesMalpighiaceae

A.Juss. ex Endl., Ann. Sci. Nat., Bot., sér. 2, 13: 266. 1840.

98EAA7E9-B318-5976-87B0-0882129F255D

##### Type species.

*Aspidopteryselliptica* (Blume) A.Juss. ex Endl.

##### Notes.

*Aspidopterys* comprises 24 currently accepted species (ten threatened species; Suppl. material 1) of lianas endemic to rainforests of Bangladesh, Cambodia, China, India, Indonesia, Laos, Malaysia, Myanmar, Nepal, Philippines, Thailand, Tibet, and Vietnam, Southeast Asia (POWO 2024). For an updated identification key for all species of *Aspidopterys*, see Hutchinson (1917) for a partial revision, Sirirugsa (1991) for Thailand, and Srivastava (1997) for India.

#### ﻿2.8.3. Calcicola

Taxon classificationPlantaeMalpighialesMalpighiaceae

W.R.Anderson & C.Davis, Contr. Univ. Michigan Herb. 25: 148. 2007.

DC5190CC-7BB9-594F-9157-63BFA2CEF28A

##### Type species.

*Calcicolaparvifolia* (A.Juss.) W.R.Anderson & C.Davis.

##### Notes.

*Calcicola* comprises only two currently accepted species (no threatened species; Suppl. material 1) of shrubs endemic to the seasonally dry tropical forests of Mexico, North America (POWO 2024). For an identification key for all species of *Calcicola*, see Anderson and Davis (2007).

#### ﻿2.8.4. Caucanthus

Taxon classificationPlantaeMalpighialesMalpighiaceae

Forssk., Fl. Aegypt.-Arab.: 91. 1775.

D63CD3F0-2AA6-5106-BB4F-E68529065571

= Caucanthussect.Eriocaucanthus Nied., Bull. Herb. Boissier, sér. 2, 4: 1010. 1904 ≡ Eriocaucanthus (Nied.) Chiov., Ann. Bot. (Rome) 10: 29. 1912. Type species: Caucanthusargenteus Nied.

##### Type species.

*Caucanthusedulis* Forssk.

##### Notes.

*Caucanthus* comprises only two currently accepted species (no threatened species; Suppl. material 1) of shrubs or lianas endemic to seasonally dry tropical forests of east Africa (Ethiopia, Kenya, Malawi, Mozambique, Somalia, Tanzania, Uganda, and Zimbabwe) and the Arabic Peninsula (Saudi Arabia and Yemen; POWO 2024). For an updated identification key for all species of *Caucanthus*, see Launert (1968).

#### ﻿2.8.5. Diaspis

Taxon classificationPlantaeMalpighialesMalpighiaceae

Nied., Bot. Jahrb. Syst. 14: 314. 1892.

8BE4F286-D348-5658-A7F5-58263EF42951

##### Type species.

*Diaspisalbida* Nied.

##### Notes.

*Diaspis* comprises a single currently accepted species (no threatened species; Suppl. material 1) of liana endemic to the seasonally dry tropical forests of Ethiopia, Kenya, and Somalia, Africa (POWO 2024). For a taxonomic treatment of *Diaspis*, see Niedenzu (1928).

#### ﻿2.8.6. Digoniopterys

Taxon classificationPlantaeMalpighialesMalpighiaceae

Arènes, Notul. Syst. (Paris) 12: 133. 1946.

256E56B3-A034-56A4-BDD8-BCDF3F9F738C

##### Type species.

*Digoniopterysmicrophylla* Arènes.

##### Notes.

*Digoniopterys* comprises a single currently accepted species (one threatened species; Suppl. material 1) of shrub endemic to the seasonally dry tropical forests of Madagascar, Africa (POWO 2024). For a taxonomic treatment of *Digoniopterys*, see Arènes (1946).

#### ﻿2.8.7. Ectopopterys

Taxon classificationPlantaeMalpighialesMalpighiaceae

W.R.Anderson, Contr. Univ. Michigan Herb. 14: 11. 1980.

940ABFB4-27D7-5CE5-BAD9-131F6E1D94FC

##### Type species.

*Ectopopteryssoejartoi* W.R.Anderson.

##### Notes.

*Ectopopterys* comprises a single currently accepted species (no threatened species; Suppl. material 1) of liana endemic to rainforests and seasonally dry tropical forests of Colombia, Ecuador, and Peru, South America (POWO 2024). For a taxonomic treatment of *Ectopopterys*, see Anderson (1980).

#### ﻿2.8.8. Madagasikaria

Taxon classificationPlantaeMalpighialesMalpighiaceae

C.Davis, Amer. J. Bot. 89: 702. 2002.

5615696D-6C54-5FB8-97BF-CF188568CD64

##### Type species.

*Madagasikariaandersonii* C.Davis.

##### Notes.

*Madagasikaria* comprises a single currently accepted species (one threatened species; Suppl. material 1) of liana endemic to the seasonally dry tropical forests of Madagascar, Africa (POWO 2024). For a taxonomic treatment of *Madagasikaria*, see Davis (2002).

#### ﻿2.8.9. Malpighia

Taxon classificationPlantaeMalpighialesMalpighiaceae

Plum. ex L., Sp. Pl. 425. 1753.

87636663-8455-5B20-B318-FF549B52ACA8

Fig. 10J–L

= Rudolphia Medik., Malvenfam.: 111. 1787. Type species: Rudolphiaedulis Medik. (= MalpighiaurensL.subsp.urens).

##### Type species.

*Malpighiaglabra* Plum. ex L.

##### Notes.

*Malpighia* comprises 110 currently accepted species (85 threatened species; Suppl. material 1) of treelets or shrubs endemic to rainforests and seasonally dry tropical forests from South America (Colombia, Ecuador, and Venezuela) to Central (Aruba, Bahamas, Belize, Cayman Islands, Costa Rica, Cuba, Dominican Republic, El Salvador, Guatemala, Haiti, Honduras, Jamaica, Leeward Islands, Netherlands Antilles, Nicaragua, Panamá, Puerto Rico, Trinidad-Tobago, Turks-Caicos Islands, and Windward Islands) and North America (Mexico and United States of America; POWO 2024). For identification keys for all species of *Malpighia*, see the monographs by Vivaldi (1979) and Meyer (2000), the studies of González-Gutiérrez and Meyer (2019) for Cuba, and Pool (2023) for Mesoamerica.

#### ﻿2.8.10. Mascagnia

Taxon classificationPlantaeMalpighialesMalpighiaceae

(Bertero ex DC.) Bertero, Hortus Ripul.: 85. 1824 nom. cons.

3F2C965E-A5D8-593A-9FDC-F1D584471F50

Figs 3E , 6B , 8Z , 11I

≡ Hiraea [unranked] Mascagnia Bertero ex DC., Prodr. 1: 585. 1824.
= Triopterys L., Sp. Pl.: 428. 1753, nom. rej. Type species: Triopterysjamaicensis L. [= Mascagnialucida (Kunth) W.R.Anderson & C.Davis].

##### Type species.

*Mascagniaamericana* Bertero [= *Mascagniamacradena* (DC.) Nied.].

##### Notes.

*Mascagnia* comprises 48 currently accepted species (19 threatened species; Suppl. material 1) of shrubs or lianas endemic to rainforests, savannas, and seasonally dry tropical forests from Mexico (North America) to Argentina (South America; POWO 2024). There is no current updated identification key for all species of *Mascagnia*, but for regional treatments, see Anderson (1981) for the Guyana Highland, Almeida et al. (2020) for Brazil, and Pool (in prep.) for Mesoamerica.

#### ﻿2.8.11. Microsteira

Taxon classificationPlantaeMalpighialesMalpighiaceae

Baker, J. Linn. Soc., Bot. 20: 111. 1883.

EB2A660E-94DD-5CA6-91EC-010CC2849A3A

##### Type species.

*Microsteiracurtisii* Baker.

##### Notes.

*Microsteira* comprises 27 currently accepted species (all threatened species; Suppl. material 1) of lianas endemic to rainforests, savannas, and seasonally dry tropical forests of Madagascar, Africa (POWO 2024). For an identification key for all species of *Microsteira*, see Arènes (1945).

#### ﻿2.8.12. Rhynchophora

Taxon classificationPlantaeMalpighialesMalpighiaceae

Arènes, Notul. Syst. (Paris) 12: 127. 1946.

7D5AFC7B-A6BE-556C-93B4-4B5B3D30E23E

= Calyptostylis Arènes, Notul. Syst. (Paris) 12: 131. 1946. Type species: Calyptostylishumbertii Arènes (= Rhynchophoraphillipsonii W.R.Anderson).

##### Type species.

*Rhynchophorahumbertii* Arènes.

##### Notes.

*Rhynchophora* comprises only two currently accepted species (all threatened species; Suppl. material 1) of lianas endemic to the seasonally dry tropical forests of Madagascar, Africa (POWO 2024). For an identification key for all species of *Rhynchophora*, see Anderson (2001a). Despite *Madagasikaria* causing the non-monophyly of *Rhynchophora* (Fig. 1), the bootstrap support value for this clade is below 60%. Therefore, we have chosen to retain both genera as independent until further phylogenetic evi­dence sheds some light on the matter.

#### ﻿2.8.13. Triaspis

Taxon classificationPlantaeMalpighialesMalpighiaceae

Burch., Trav. S. Africa 2: 280. 1824.

1B086974-BDE0-5DCB-97AA-964C78644CBD

= Umbellulanthus S.Moore, J. Bot. 58: 220. 1920. Type species: Umbellulanthusfloribundus S.Moore (≡ Triaspismooreana Exell & Mendonça).

##### Type species.

*Triaspishypericoides* Burch.

##### Notes.

*Triaspis* comprises 19 currently accepted species (five threatened species; Suppl. material 1) endemic to rainforests, savannas, and seasonally dry tropical forests of Angola, Benin, Botswana, Cameroon, Cape Green, Congo, Ethiopia, Gabon, Ghana, Guinea, Ivory Coast, Kenya, Liberia, Malawi, Mozambique, Namibia, Nigeria, Sierra Leone, Somalia, South Africa, Tanzania, Togo, Zambia, Zaire, and Zimbabwe, Africa (POWO 2024). There is no current identification key for all species of *Triaspis*, but for regional keys, see Niedenzu (1928), Launert (1968) for East Africa, Badré (1972) for Cameroon, Badré (1973) for Gabon, Hutchinson and Dalziel (1958) for West Tropical Africa, Wilczek (1958) for Democratic Republic of Congo, and Almeida et al. (2024) for Southern Africa.

#### ﻿2.9. Gaudichaudieae

Taxon classificationPlantaeMalpighialesMalpighiaceae

Horan., Char. Ess. Fam.: 182. 1847, emend. nov. R.F.Almeida

5B8AFA16-06EC-57B1-A48B-E791697BE053

≡ Gaudichaudioideae A.Juss. ex C.V.Morton, Taxon 17: 318. 1968.
= Sphedamnocarpinae Nied. in Engler & Prantl, Nat. Pflanzenfam. III, 4: 52, 59. 1890, syn. nov. Type genus: Sphedamnocarpus Planch. ex Benth. & Hook.f.

##### Type genus.

*Gaudichaudia* Kunth.

##### Diagnosis.

Lianas, shrubs to subshrubs; umbels, rarely thyrses, usually 4-flowered; pollen 4–12-pantocolporate (porate in Stigmaphyllonsubg.Ryssopterys, *Philgamia*, and *Sphedamnocarpus*); mericarps winged, 1-winged, dorsal wing more developed, rarely reduced, presence of macrolactams, absence of biotin and derivatives, sulfenyl compounds.

##### Notes.

Gaudichaudieae currently comprises 14 accepted genera, *Aspicarpa*, *Banisteriopsis*, *Bronwenia*, *Camarea*, *Cottsia*, *Diplopterys*, *Janusia*, *Mamedea*, *Mionandra*, *Peixotoa*, *Philgamia*, *Schwannia*, *Sphedamnocarpus*, and *Stigmaphyllon*, and 336 species (154 threatened species; Suppl. material 1) occurring in the Americas, Africa, Asia and Oceania (POWO 2024).

Anderson (1993) proposed Gaudichaudieae (A.Juss.) W.R.Anderson with the aim to “validate” the name published by Jussieu (1840). Nonetheless, the name published by Jussieu (1840) was proposed as unranked and was not validly published until Morton (1968) provided a Latin diagnosis while also correcting its spelling and rank to be used as a subfamily. However, both authors overlooked that the name had already been validly published as a tribe, accompanied by a Latin diagnosis by Horaninow (1847). Therefore, Anderson’s name is a superfluous, later homonym of Gaudichaudieae Horan.

### ﻿Key to the genera of Gaudichaudieae

**Table:** 

1	Petiole or leaf base glands ellipsoid, sunken; flowers arranged in thyrses, secondarily arranged in thyrses; sepal glands decurrent into the pedicel, stamens homomorphic	***Bronwenia* (Figs 3A, 8H, R, V, 9E, L)**
–	Petioles or leaf base glands discoid or cupuliform, not sunken; flowers arranged in corymbs or umbels, solitary or secondarily arranged in dichasia or thyrses, rarely solitary thyrses; sepals not decurrent, stamens heteromorphic	2
2	Leaves apex long-acuminate; petals abaxially pubescent, styles pubescent; mericarps with dominant lateral wings	***Diplopterys* (Figs 8S, 9O, 11A)**
–	Leaves apex emarginate, rounded, obtuse, acute, short-acuminate or acuminate; petals abaxially glabrous, styles glabrous; mericarps with a dominant dorsal wing, lateral wings reduced or absent	**3**
3	Branches with scale-like hairs; leaves long-petiolate, rarely short-petiolate; flowers arranged in corymbs or umbels, 5–many-flowered, peduncle curved; styles divergent and lyrate, apex expanded (foliaceous), rarely reduced	***Stigmaphyllon* (Figs 3C, N, 4A–C, E–G, I–N, 6D, E, 9N, P, Q)**
–	Branches without scale-like hairs; leaves short-petiolate; flowers arranged in umbels or thyrses, 1–4-flowered, peduncle straight; styles parallel and straight, apex truncate or cylindrical	**4**
4	Cincinni sessile or short-pedunculate; styles 3	**5**
–	Cincinni long-pedunculate; style 1(–2–3), if styles 2–3 then carpels slightly rotated so that no carpel aligns with the anterior sepals and posterior petal, and mericarps with dominant lateral wings	**9**
5	Stipules minute, free; flowers chasmogamous, sepal apex straight, fertile stamens 10, staminodes absent	**6**
–	Stipules expanded, fused or bifid; flowers chasmogamous or cleistogamous, sepal apex revolute or involute along margins, fertile stamens 5, staminodes 3–5	**8**
6	Flowers zygomorphic, sepals at anthesis bent towards the centre of the flower, connectives glandular; Neotropics	***Banisteriopsis* (Figs 3M–S, 4B, D, O, R, T, V, 6C, 7F, 9A–D, H, 11B)**
–	Flowers actinomorphic, sepals erect at anthesis, connectives eglandular; Africa	**7**
7	Petals yellow; mericarp dorsal wing well-developed; Africa and Madagascar	***Sphedamnocarpus***
–	Petals white; mericarp dorsal wing absent or very reduced; Madagascar	***Philgamia***
8	Stipules connate at base or up to the middle (i.e., bifid); flowers arranged in umbels, 1-flowered, bract and bracteoles absent; sepals free, completely revolute or involute along margins, antherodes filiform, minute, styles apex truncate to slightly expanded	***Mionandra***
–	Stipules connate (i.e., entire); flowers arranged in umbels, 4-flowered, bract and bracteoles present; sepals connate at base, revolute only at apex, antherodes globose, conspicuous, styles apex capitate	***Peixotoa* (Figs 3H, 8X, 9F, 11E)**
9	Flowers chasmogamous, fertile stamens 2, staminodes 3, antherodes absent, carpels syncarpic	***Cottsia***
–	Flowers chasmogamous or cleistogamous, fertile stamens 3–4–5–6, staminodes 0–2, antherodes present, carpels syncarpic at base and apically apocarpic	**10**
10	Flowers enantiostylous, petal margin long-fimbriate, fertile stamens 6, style curved	***Schwannia* (Figs 3P, 8W, 9G, M)**
–	Flowers non-enantiostylous, petal margin entire, erose, denticulate or dentate, rarely short-fimbriate, fertile stamens 3–4–5, style straight	11
11	Fertile stamens 5, staminodes absent; mericarp dorsal wing developed	***Janusia***
–	Fertile stamens 3–4(–5), staminodes present; mericarp dorsal wing absent or reduced to a crest	**12**
12	Branches herbaceous, annual, with acicular hairs; leaf blades reduced and narrow, margin usually revolute; fertile stamens 4, homomorphic	***Camarea* (Figs 7L, 8I, 10V)**
–	Branches woody, perennial, with malpighiaceous hairs; leaf blades usually expanded and broad, margin plane; fertile stamens 3(–5), heteromorphic	**13**
13	Leaf base never with filamentous or tooth-like projections; flowers arranged in 1–4-flowered umbels, peduncles absent to reduced, without associated reduced leaves; antherodes equalling or larger than anthers (reduced to an apical swelling in *M.harleyi* and *M.lanata*), pubescent (glabrous in *M.harleyi*), usually red to orange at post-anthesis, 2 posterior carpels rotated so that all face the posterior petal; mericarp wings reduced to crests to teeth; central to southern South America	***Mamedea* (Fig. 4U)**
–	Leaf base generally with a filamentous or tooth-like projection at each side of the blade; flowers arranged in 3–4-flowered umbels, peduncles long, with associated reduced leaves; antherodes smaller than anthers (few species with all anthers fertile), glabrous, yellow at post-anthesis, carpels slightly rotated so that no carpel aligns with the anterior sepals and posterior petal; mericarp lateral wings dominant (often fused), sometimes all wings equally developed into wings or reduced to crests; North and Central America	***Aspicarpa***

#### ﻿2.9.1. Aspicarpa

Taxon classificationPlantaeMalpighialesMalpighiaceae

Rich., Mém. Mus. Hist. Nat. 2: 396–400, pl. 13. 1815.

D3A63C4E-AAE8-51DA-BD5D-15BA083BF939

= Acosmus Desv., J. Bot. Agric. 3: 229. 1816. Type species: Acosmuspruriens Desv. (=Aspicarpahirtella Rich.).
= Gaudichaudia Kunth, Nov. Gen. Sp. (quarto ed.) 5: pl. 445. 1821, syn. nov. Type species: Gaudichaudiacynanchoides Kunth [≡ Aspicarpacynanchoides (Kunth) Hassl.].
= Gaudichaudia [unranked] Tritomopterys A.Juss. ex Endl., Gen. Pl. 1058. 1840 ≡ Tritomopterys (A.Juss. ex Endl.) Nied., Arbeiten Bot. Inst. Königl. Lyceums Hosianum Braunsberg 4: 28. 1912. Type species (designated here): Gaudichaudiaconfertiflora A.Juss. [≡ Aspicarpaconfertiflora (A.Juss.) R.F.Almeida & M.Pell.].
= Rosanthus Small in Britton & al., N. Amer. Fl. 25: 131. 1910. Type species: Rosanthussubverticellatus (Rose) Small [≡ Aspicarpasubverticillata (Rose) Hassl.].

##### Type species.

*Aspicarpahirtella* Rich.

##### Notes.

In its current circumscription, *Aspicarpa* (now including *Gaudichaudia*) comprises 27 species (ten threatened species; Suppl. material 1) of shrubs, subshrubs or lianas with a long and tortuous taxonomic history. Most species have already been placed in the genera *Banisteria* (= *Heteropterys* Kunth), *Gaudichaudia*, *Hiraea*, *Triopterys* [= *Mascagnia* (Bertero ex DC.) Bertero], and *Tritomopterys*. However, *Aspicarpasensu* W.R.Anderson is greatly non-monophyletic, with a South American clade recovered sister to *Janusia* s.str. and the mostly North and Central American species recovered mixed with *Gaudichaudia*. Thus, *Gaudichaudia* and the mostly North and Central American species of *Aspicarpa* are combined here, while the exclusively South American clade is proposed as a new genus, *Mamedea* (see below).

Most of the morphological diversity found in *Aspicarpa* s.lat. (especially the production of cleistogamous flowers and variation in the number of style number) might be attributed to polyploidy events (Jessup 2003). *Aspicarpa* species occur in seasonally dry tropical forests from North America (Mexico and the United States), Central America (Costa Rica, El Salvador, Guatemala, Honduras, and Nicaragua), to northern South America (Colombia and Venezuela; POWO 2024). No complete revision is available for the current circumscription of *Aspicarpa* or any of the previous circumscriptions of *Aspicarpa* and *Gaudichaudia*. A taxonomic revision of this genus is urgently needed, and species boundaries are especially fuzzy in the former *Gaudichaudia*.

#### ﻿2.9.1.a. Aspicarpa andersonii

Taxon classificationPlantaeMalpighialesMalpighiaceae

(S.L.Jessup) R.F.Almeida & M.Pell. comb. nov.

53C14CC2-E0D4-5BE8-A3F2-89A5919242D4

urn:lsid:ipni.org:names:77342400-1

≡ Gaudichaudiaandersonii S.L.Jessup, Madroño 49: 254. 2002.

#### ﻿2.9.1.b. Aspicarpa arnottiana

Taxon classificationPlantaeMalpighialesMalpighiaceae

(A.Juss.) R.F.Almeida & M.Pell. comb. nov.

C25C615C-153B-59B3-B20F-2A6DED0531C2

urn:lsid:ipni.org:names:77342401-1

≡ Gaudichaudiaarnottiana A.Juss., Ann. Sci. Nat., Bot., sér. 2, 13: 252. 1840.

#### ﻿2.9.1.c. Aspicarpa chasei

Taxon classificationPlantaeMalpighialesMalpighiaceae

(W.R.Anderson) R.F.Almeida & M.Pell. comb. nov.

8AA63670-CCD8-50EF-9B58-EED9BB60DEC1

urn:lsid:ipni.org:names:77342402-1

≡ Gaudichaudiachasei W.R.Anderson, Contr. Univ. Michigan Herb. 16: 68. 1987.

#### ﻿2.9.1.d. Aspicarpa confertiflora

Taxon classificationPlantaeMalpighialesMalpighiaceae

(A.Juss.) R.F.Almeida & M.Pell. comb. nov.

EDA6FE12-344E-586D-A553-4DB8C0F67807

urn:lsid:ipni.org:names:77342403-1

≡ Gaudichaudiaconfertiflora A.Juss., Ann. Sci. Nat., Bot., sér. 2, 13: 252. 1840.

#### ﻿2.9.1.e. Aspicarpa cycloptera

Taxon classificationPlantaeMalpighialesMalpighiaceae

(DC.) R.F.Almeida & M.Pell. comb. nov.

51BCCC21-8646-5CE2-8263-8292BE4E6368

urn:lsid:ipni.org:names:77342404-1

≡ Hiraeacycloptera DC., Prodr. 1: 586. 1824.

#### ﻿2.9.1.f. Aspicarpa filipendula

Taxon classificationPlantaeMalpighialesMalpighiaceae

(A.Juss.) R.F.Almeida & M.Pell. comb. nov.

BF055656-0F74-5904-9A98-BCE7EF5C4E26

urn:lsid:ipni.org:names:77342405-1

≡ Gaudichaudiafilipendula A.Juss., Ann. Sci. Nat., Bot., sér. 2, 13: 252. 1840.

#### ﻿2.9.1.g. Aspicarpa implexa

Taxon classificationPlantaeMalpighialesMalpighiaceae

(S.L.Jessup) R.F.Almeida & M.Pell. comb. nov.

80873FCC-1EA6-586A-8BD9-52F81A3492D3

urn:lsid:ipni.org:names:77342406-1

≡ Gaudichaudiaimplexa S.L.Jessup, Madroño 49: 247. 2002.

#### ﻿2.9.1.h. Aspicarpa intermixteca

Taxon classificationPlantaeMalpighialesMalpighiaceae

(S.L.Jessup) R.F.Almeida & M.Pell. comb. nov.

073DFE34-48E2-555A-9AD4-8564A30B1D12

urn:lsid:ipni.org:names:77342407-1

≡ Gaudichaudiaintermixteca S.L.Jessup, Madroño 49: 251. 2002.

#### ﻿2.9.1.i. Aspicarpa krusei

Taxon classificationPlantaeMalpighialesMalpighiaceae

(W.R.Anderson) R.F.Almeida & M.Pell. comb. nov.

4C890914-F323-518C-958A-BEB72F429259

urn:lsid:ipni.org:names:77342408-1

≡ Gaudichaudiakrusei W.R.Anderson, Contr. Univ. Michigan Herb. 16: 69. 1987.

#### ﻿2.9.1.j. Aspicarpa mcvaughii

Taxon classificationPlantaeMalpighialesMalpighiaceae

(W.R.Anderson) R.F.Almeida & M.Pell. comb. nov.

107318E9-4FA5-5B8E-B44B-FE22D2C44934

urn:lsid:ipni.org:names:77342409-1

≡ Gaudichaudiamcvaughii W.R.Anderson, Contr. Univ. Michigan Herb. 16: 72. 1987.

#### ﻿2.9.1.k. Aspicarpa oxyota

Taxon classificationPlantaeMalpighialesMalpighiaceae

(DC.) R.F.Almeida & M.Pell. comb. nov.

95D3F418-2C09-56E2-922C-079A2B2715FB

urn:lsid:ipni.org:names:77342410-1

≡ Hiraeaoxyota DC., Prodr. 1: 586. 1824.

#### ﻿2.9.1.l. Aspicarpa palmeri

Taxon classificationPlantaeMalpighialesMalpighiaceae

(S.Watson) R.F.Almeida & M.Pell. comb. nov.

03D435E3-8080-59CA-B23D-E5D1F32FDAD3

urn:lsid:ipni.org:names:77342411-1

≡ Gaudichaudiapalmeri S.Watson, Proc. Amer. Acad. Arts 21: 421. 1886.

#### ﻿2.9.1.m. Aspicarpa symplecta

Taxon classificationPlantaeMalpighialesMalpighiaceae

(S.L.Jessup) R.F.Almeida & M.Pell. comb. nov.

660F2873-3C84-5DCB-BC30-4A98C4D88A37

urn:lsid:ipni.org:names:77342412-1

≡ Gaudichaudiasymplecta S.L.Jessup, Madroño 49(4): 253. 2002.

#### ﻿2.9.1.n. Aspicarpa synoptera

Taxon classificationPlantaeMalpighialesMalpighiaceae

(S.L.Jessup) R.F.Almeida & M.Pell. comb. nov.

9BC0C044-1E63-56C3-9321-EA4C72A1C2A6

urn:lsid:ipni.org:names:77342413-1

≡ Gaudichaudiasynoptera S.L.Jessup, Madroño 49(4): 251. 2002.

#### ﻿2.9.1.o. Aspicarpa zygoptera

Taxon classificationPlantaeMalpighialesMalpighiaceae

(S.L.Jessup) R.F.Almeida & M.Pell. comb. nov.

F384F884-A3A2-5A05-8190-75419B96686E

urn:lsid:ipni.org:names:77342414-1

≡ Gaudichaudiazygoptera S.L.Jessup, Madroño 49: 249. 2002.

#### ﻿2.9.2. Banisteriopsis

Taxon classificationPlantaeMalpighialesMalpighiaceae

C.R.Rob. in Britton & al., N. Amer. Fl. 25(2): 131. 1910.

5FE09D59-39F5-5298-99BD-73B8E97454EC

Figs 3M–O, Q–S , 4B, D, O, R, T, V , 6C , 7F , 9A–D, H , 11B

##### Type species.

*Banisteriopsisargentea* (Kunth) C.R.Rob. [= *Banisteriopsismuricata* (Cav.) Cuatrec.]

##### Notes.

*Banisteriopsis* comprises 65 currently accepted species (24 threatened species; Suppl. material 1) of treelets, shrubs, subshrubs or lianas, endemic to rainforests, savannas, and seasonally dry tropical forests from Mexico (North America) to Argentina (South America; POWO 2024). For an identification key for all species of *Banisteriopsis*, see Gates (1982) and Almeida et al. (2020).

#### ﻿2.9.2.a. Banisteriopsis appressa

Taxon classificationPlantaeMalpighialesMalpighiaceae

(B.Gates) R.F.Almeida & M.Pell. stat. nov.

1E763840-EA32-51FD-AB8C-4310B7C93DCA

urn:lsid:ipni.org:names:77342415-1

≡ Banisteriopsismalifoliavar.appressa B.Gates, Fl. Neotrop. Monogr. 30: 79. 1982.

#### ﻿2.9.2.b. Banisteriopsis subenervia

Taxon classificationPlantaeMalpighialesMalpighiaceae

(B.Gates) R.F.Almeida & M.Pell. stat. nov.

8CB3F849-B3A0-5D1F-9BFB-FF3AE1D57487

urn:lsid:ipni.org:names:77342416-1

≡ Banisteriopsismartinianavar.subenervia Cuatrec., Webbia 13: 501. 1958.

#### ﻿2.9.2.c. Banisteriopsis glabrata

Taxon classificationPlantaeMalpighialesMalpighiaceae

(B.Gates) R.F.Almeida & M.Pell. stat. nov.

E36CC462-E866-5562-A4EB-1A21E0F5472D

urn:lsid:ipni.org:names:77342417-1

≡ Banisteriopsispulchravar.glabrata B.Gates, Fl. Neotrop. Monogr. 30: 109. 1982.

#### ﻿2.9.3. Bronwenia

Taxon classificationPlantaeMalpighialesMalpighiaceae

W.R.Anderson & C.Davis, Contr. Univ. Michigan Herb. 25: 138–140. 2007.

EEF0314D-D12B-5068-9811-0B91CF218289

Figs 3A , 8H, R, V , 9E, L

##### Type species.

*Bronweniaferruginea* (Cav.) W.R.Anderson & C.Davis.

##### Notes.

*Bronwenia* comprises 13 currently accepted species (four threatened species; Suppl. material 1) of shrubs or lianas endemic to rainforests and seasonally dry tropical forests from Mexico (North America) to Brazil (South America; POWO 2024). For an identification key for all species of *Bronwenia*, see Gates (1982) and Anderson and Davis (2007).

#### ﻿2.9.3.a. Bronwenia llanensis

Taxon classificationPlantaeMalpighialesMalpighiaceae

(B.Gates) R.F.Almeida & M.Pell. stat. nov.

F6C4F3FC-5861-5265-9627-E9D415FCF781

urn:lsid:ipni.org:names:77342418-1

≡ Banisteriopsisacapulcensisvar.llanensis B.Gates, Fl. Neotrop. Monogr. 30: 46. 1982 ≡ Bronweniaacapulcensisvar.llanensis (B.Gates) W.R.Anderson & C.Davis, Contr. Univ. Michigan Herb. 25: 141. 2007.

#### ﻿2.9.3.b. Bronwenia maracaybensis

Taxon classificationPlantaeMalpighialesMalpighiaceae

(A.Juss.) R.F.Almeida & M.Pell., comb. et stat. nov.

E23844D4-8902-534D-91E8-1C6BA97E8AB1

urn:lsid:ipni.org:names:77342419-1

≡ Banisteriamaracaybensis A.Juss., Ann. Sci. Nat., Bot., sér. 2, 13: 285. 1840 ≡ Banisteriopsiscornifoliavar.maracaybensis (A.Juss.) W.R.Anderson, Contr. Univ. Michigan Herb. 20: 15. 1995 ≡ Bronweniacornifoliavar.maracaybensis (A.Juss.) W.R.Anderson & C.Davis, Contr. Univ. Michigan Herb. 25: 143. 2007.

#### ﻿2.9.3.c. Bronwenia standleyi

Taxon classificationPlantaeMalpighialesMalpighiaceae

(B.Gates) R.F.Almeida & M.Pell., comb. et stat.

597CF429-92D5-5978-A572-2473EFB42E0B

urn:lsid:ipni.org:names:77342420-1

≡ Banisteriopsiscornifoliavar.standleyi B.Gates, Fl. Neotrop. Monogr. 30: 44. 1982 ≡ Bronweniacornifoliavar.standleyi (B.Gates) W.R.Anderson & C.Davis, Contr. Univ. Michigan Herb. 25: 143. 2007.

#### ﻿2.9.4. Camarea

Taxon classificationPlantaeMalpighialesMalpighiaceae

A.St.-Hil., Bull. Philom.: 133. 1823.

6A1FD7B9-C6D5-5DA2-843D-D414C00D1E1D

Figs 7L , 8I , 10V

= Camareasect.Cryptolappa A.Juss., Ann. Sci. Nat., Bot., sér. 2, 13: 254. 1840 ≡ Cryptolappa (A.Juss.) Kuntze, Revis. Gen. Pl. 1: 88. 1891. Type species: Camareaaffinis A.St.-Hil.

##### Type species.

*Camareaericoides* A.St.-Hil.

##### Notes.

*Camarea* comprises eight currently accepted species (three threatened species; Suppl. material 1) of subshrubs endemic to savannas and campos rupestres of Bolivia, Brazil, Guyana, Paraguay, and Suriname, South America (POWO 2024). *Camareaglazioviana* Nied. and *Camareatriphylla* Mart. ex A.Juss. are listed by POWO (2024) as accepted but represent synonyms of *Camareasericea* A.St.-Hil. and *Camareaaxillaris* A.St.-Hil., respectively. Alternatively, *Camarealinearifolia* A.St.-Hil. is listed by POWO (2024) as a synonym of *Camareaericoides* A.St.-Hil., but actually represents a distinct species. For an identification key for all species of *Camarea*, see Mamede (1990) and Almeida et al. (2020).

#### ﻿2.9.5. Cottsia

Taxon classificationPlantaeMalpighialesMalpighiaceae

Dubard & Dop, Rev. Gén. Bot. 20: 359. 1908.

224CF096-19B7-5E30-8603-E5469DD9CB68

= Janusiasect.Metajanusia Nied., Verz. Vorles. Königl. Lyceum Hosianum Braunsberg 1912–1913: 50. 1912 ≡ Gaudichaudiasect.Erostratae Chodat, Bull. Soc. Bot. Genève, sér. 2, 9: 100. 1917, nom. superfl. ≡ Aspicarpasect.Metajanusia (Nied.) Hassl., Annuaire Conserv. Jard. Bot. Genève 20: 212. 1918. Type species: Janusiagracilis A.Gray [≡ Cottsiagracilis (A.Gray) W.R.Anderson & C.Davis].

##### Type species.

*Cottsiascandens* Dubard & Dop [= *Cottsiacalifornica* (Benth.) W.R.Anderson & C.Davis].

##### Notes.

*Cottsia* comprises four currently accepted species (one threatened species; Suppl. material 1) of lianas endemic to the seasonally dry tropical forests of Mexico and the United States, North America (POWO 2024). For an identification key for all species of *Cottsia*, see Anderson and Davis (2007).

#### ﻿2.9.6. Diplopterys

Taxon classificationPlantaeMalpighialesMalpighiaceae

A.Juss. in Deless., Icon. Sel. Pl. 3: 20, pl. 33. 1837.

BBF1FA1C-906F-574D-868A-D95E060166DA

Figs 8S , 9O , 11A

= Jubistylis Rusby, Mem. New York Bot. Gard. 7: 273. 1927. Type species: Jubistylismollis Rusby [= Diplopteryslutea (Ruiz ex Griseb.) W.R.Anderson & C.Davis].

##### Type species.

*Diplopterysparalias* A.Juss. [=*Diplopteryspauciflora* (G. Mey.) Nied.]

##### Notes.

*Diplopterys* comprises 31 currently accepted species (11 threatened species; Suppl. material 1) of shrubs or lianas endemic to rainforests, savannas, and seasonally dry tropical forests from Mexico (North America) to Argentina (South America; POWO 2024). For an identification key for all species of *Diplopterys*, see Gates (1982).

#### ﻿2.9.7. Janusia

Taxon classificationPlantaeMalpighialesMalpighiaceae

A.Juss. ex Endl., Arch. Mus. Par. 3: 608. 1843.

B0EB4146-4A75-53C5-AE88-307597FC5CD1

= Peregrina W.R.Anderson, Syst. Bot. 10(3): 303. 1985, **syn. nov.** Type species: Peregrinalinearifolia (A.St.-Hil.) W.R.Anderson [≡ Janusialinearifolia (A.St.-Hil.) A.Juss.].

##### Type species.

*Janusiaguaranitica* (A.St.-Hil.) A.Juss. ex Endl.

##### Notes.

With the reestablishment of *Schwannia*, the recognition of *Peregrina* as independent of *Janusia* based only on the subshrub habit (vs liana) and laterally flattened stigmas (vs rounded stigmas) unnecessarily inflates the number of genera in Malpighiaceae without providing any taxonomic or systematic benefits. Since *Janusia* and *Peregrina* share non-enantioustilous flowers and androecia with five fertile stamens, without staminodes, we choose to return *Peregrina* to *Janusia*. In its current sense, *Janusia* comprises only two currently accepted species (one threatened species; Suppl. material 1) of subshrubs or lianas endemic to rainforests, savannas, and seasonally dry tropical forests of Argentina, Bolivia, Brazil, Paraguay, and Uruguay, South America (POWO 2024). For an identification key for all species of *Janusia*, see Sebastiani (2010).

#### ﻿2.9.8. Mamedea

Taxon classificationPlantaeMalpighialesMalpighiaceae

R.F.Almeida & M.Pell. gen. nov.

8D9EAC87-D4D2-5EBA-BDC7-12D8D679FC7F

urn:lsid:ipni.org:names:77342421-1

Fig. 4U

##### Type species.

*Mamedeapulchella* (Griseb.) R.F.Almeida & M.Pell.

##### Diagnosis.

*Mamedea* can be recognised by its erect shrub to subshrub habit, present xylopodium, leaves entire at base, umbels, 1–4-flowered, peduncle usually absent or reduced, not bearing reduced leaves, sepals bent inwards between the petals at anthesis, petals fimbriate, androecium with 3 fertile stamens, anthers glabrous to pubescent, staminodes 2, antherodes present or not, when present larger than the fertile anthers, glabrous to pubescent, usually red to orange at post-anthesis, 2 posterior carpels rotated so that all face the posterior petal, mericarps with dorsal and lateral wings reduced to ribs or teeth, and with a rugose nut, chromosome number *n* = (20–)40.

##### Description.

***Shrubs to subshrubs***. ***Roots*** fibrous, woody near the xylopodium. ***Xylopodium*** present, small to large. ***Branches*** erect, slender, woody to herbaceous, sometimes brittle, sericeous to glabrescent; internodes inconspicuous to elongated. ***Stipules*** interpetiolar, minute, free to connate, sericeous or distally glabrous, deciduous or persistent. ***Leaves*** opposite or decussate; petioles short, sericeous, tomentose, lanate or glabrescent, eglandular; lamina entire, elliptical, lanceolate to ovate, velutinous, sericeous, lanate or tomentose, base cuneate or rounded, margin entire, apex acute, obtuse, rounded or mucronate; venation eucamptodromous or brochidodromous, secondary veins strongly ascending and subparallel. ***Umbels*** solitary, axillary, (1–)2–4-flowered, sessile to pedunculate; inflorescence leaves not reduced; bract alternate, minute, plane, persistent, sericeous to glabrous, eglandular, persistent; cincinni (1–)2–4, alternate, 1-flowered, pedunculate; bracteoles opposite, minute, plane, persistent, sericeous to glabrous, eglandular, persistent. ***Flowers*** chasmogamous or cleistogamous, bisexual, zygomorphic, hypogynous; pedicel elongated, longer or shorter than the peduncle, sparsely sericeous, tomentose, velutinous or glabrescent; sepals 5, free valvate in bud, erect in bud, bent inwards between the petals at anthesis, triangular to broadly ovate, sericeous or tomentose, apex acute, the anterior eglandular and narrower, the lateral 4 biglandular, the glands green, yellowish-green, dark red, or reddish-purple, secreting oil, in fruit persistent, somewhat accrescent, enclosing nutlets until maturity; petals 5, imbricate in bud, yellow to orange-yellow at anthesis, glabrous or abaxially sparsely tomentose, limb plane, margin short-fimbriate, basal fimbriae mostly tipped with tiny glands, posterior petal with claw slightly thicker, sometimes with a pair of glands near the limb, limb slightly broader than the 4 lateral ones; androecium with 5 stamens, filaments free or connate at base with adjacent filaments, fertile stamens 3, opposite anterior and posterior-lateral sepals, heteromorphic, filaments stout, glabrous, anthers rimose, glabrous or locules tomentose at apex, connective glandular; staminodes 2, opposite anterior-lateral sepals, homomorphic, filaments slender, antherode equalling or larger than anthers (reduced to an apical swelling in *M.harleyi* and *M.lanata*), glandular, pubescent (glabrous in *M.harleyi*); ovary superior, 3-carpellate, carpels syncarpic, the posterior 2 rotated so that all face the posterior petal, minutely puberulent, style 1, basal, straight, glabrous, borne low on inner face of anterior carpel, stigma terminal, truncate, held above anthers or at the same level at anthesis. ***Schizocarp*** with 3 mericarps, dorsal and lateral wings reduced to ribs or teeth, glabrous to velutine; carpophore absent. ***Chromosome number****n* = (20–)40.

##### Etymology.

The genus name honours the Brazilian botanist Dra Maria Candida Henrique Mamede (b. 1956), friend, colleague, and long-time contributor to the Brazilian Malpighiaceae.

##### Notes.

*Mamedea* currently comprises seven accepted species (one threatened species; Suppl. material 1) of shrubs or subshrubs endemic to altitudinal grasslands, savannas, campos rupestres, and seasonally dry tropical forests of Argentina, Bolivia, Brazil, Paraguay, and Uruguay, South America. For partial identification keys, see Almeida et al. (2020) for Brazil and Aliscioni and Torretta (2018, under *Aspicarpa*) for Argentina.

#### ﻿2.9.8.a. Mamedea boliviense

Taxon classificationPlantaeMalpighialesMalpighiaceae

(Nied.) R.F.Almeida & M.Pell. comb. nov.

4982FA82-A827-5491-8D8D-A657851BF725

urn:lsid:ipni.org:names:77342422-1

≡ Aspicarpaboliviensis Nied., Meded. Rijks-Herb. 19: 72. 1913.

#### ﻿2.9.8.b. Mamedea harleyi

Taxon classificationPlantaeMalpighialesMalpighiaceae

(W.R.Anderson) R.F.Almeida & M.Pell. comb. nov.

77967168-E055-5489-8AFB-28C3CE5A520C

urn:lsid:ipni.org:names:77342423-1

≡ Aspicarpaharleyi W.R.Anderson, Contr. Univ. Michigan Herb. 16: 55. 1987.

#### ﻿2.9.8.c. Mamedea pulchella

Taxon classificationPlantaeMalpighialesMalpighiaceae

(Griseb.) R.F.Almeida & M.Pell. comb. nov.

7E729492-3FFA-5998-80E6-2CCEC81D8640

urn:lsid:ipni.org:names:77342424-1

≡ Camareapulchella Griseb. in Martius, Fl. Bras. 12(1): 105. 1858.

#### ﻿2.9.8.d. Mamedea lanata

Taxon classificationPlantaeMalpighialesMalpighiaceae

(Chodat) R.F.Almeida & M.Pell. comb. nov.

972BA927-5E7C-5619-8CE4-F5FBD1D13950

urn:lsid:ipni.org:names:77342425-1

≡ Camarealanata Chodat, Mém. Soc. Phys. Genève 31(2): 20. 1892 ≡ Aspicarpaschininii W.R.Anderson, Contr. Univ. Michigan Herb. 16: 59. 1987.

#### ﻿2.9.8.e. Mamedea salicifolia

Taxon classificationPlantaeMalpighialesMalpighiaceae

(Chodat.) R.F.Almeida & M.Pell. comb. nov.

7502BDF4-8008-513C-A260-DA1F47D596CB

urn:lsid:ipni.org:names:77342426-1

≡ Camareasalicifolia Chodat, Arch. Sci. Phys. Nat., sér. 3, 24: 500. 1890.

#### ﻿2.9.8.f. Mamedea sericea

Taxon classificationPlantaeMalpighialesMalpighiaceae

(Griseb.) R.F.Almeida & M.Pell. comb. nov.

776FD9A8-C391-5107-A480-7A0CAE97B690

urn:lsid:ipni.org:names:77342427-1

≡ Aspicarpasericea Griseb., Abh. Königl. Ges. Wiss. Göttingen 24: 68. 1879.

#### ﻿2.9.8.g. Mamedea uruguariensis

Taxon classificationPlantaeMalpighialesMalpighiaceae

(Nied.) R.F.Almeida & M.Pell. comb. nov.

8BB1F006-1D31-5108-B1D4-7C5D7F93774E

urn:lsid:ipni.org:names:77342428-1

≡ Aspicarpauruguariensis Nied., Verzeichnis Vorles. Konigl. Akad. Braunsberg 1912/13: 62. 1912.

#### ﻿2.9.9. Mionandra

Taxon classificationPlantaeMalpighialesMalpighiaceae

Griseb., Abh. Königl. Ges. Wiss. Göttingen 19: 101. 1874.

F12D22F9-2D3D-5BF6-8160-5DF3C416CC50

= Brittonella Rusby, Bull. Torrey Bot. Club 20: 429. 1893. Type species: Brittonellapilosa Rusby [= Mionandracamareoides Griseb.].
= Cordobia Nied., Verzeichnis Vorles. Konigl. Akad. Braunsberg 1912–13: 41. 1912. Type species: Cordobiaargentea (Griseb.) Nied. [≡ Mionandraargentea Griseb.].
= Gallardoa Hicken, Physis (Buenos Aires) 2: 101. 1916. Type species: Gallardoafischeri Hicken [≡ Mionandrafischeri (Hicken) R.F.Almeida]

##### Type species.

*Mionandracamareoides* Griseb.

##### Notes.

*Mionandra* comprises four currently accepted species (one threatened species; Suppl. material 1) of shrubs endemic to savannas and seasonally dry tropical forests of Argentina, Bolivia, and Paraguay, South America (Almeida et al. 2023b). For an identification key for all species of *Mionandra*, see Almeida et al. (2023b).

#### ﻿2.9.10. Peixotoa

Taxon classificationPlantaeMalpighialesMalpighiaceae

A.Juss., Fl. Bras. Merid. (quarto ed.) 3(22): 59. 1832 [1833].

C2696B64-80EE-5311-AAB0-DEAB75ADA770

Figs 3H , 8X , 9F , 11E

##### Type species.

*Peixotoaglabra* A.Juss.

##### Notes.

*Peixotoa* comprises 29 currently accepted species (18 threatened species; Suppl. material 1) of shrubs, subshrubs or lianas endemic to rainforests, savannas, and seasonally dry tropical forests of Bolivia, Brazil, and Paraguay, South America (POWO 2024). For an identification key for all species of *Peixotoa*, see Anderson (1982, 2001b).

#### ﻿2.9.11. Philgamia

Taxon classificationPlantaeMalpighialesMalpighiaceae

Baill., Hist. Phys. Madagascar 35, tome 5 (Atlas 3): pl. 265. 1894.

C95C0465-3989-58DF-95B8-E279C28638B8

##### Type species.

*Philgamiahibbertioides* Baill.

##### Notes.

*Philgamia* comprises four currently accepted species (all threatened species; Suppl. material 1) of shrubs endemic to grasslands and savannas of Madagascar, Africa (POWO 2024). For an identification key for all species of *Philgamia*, see Arènes (1943a).

#### ﻿2.9.12. Schwannia

Taxon classificationPlantaeMalpighialesMalpighiaceae

Endl., Gen. Plan.: 1058. 1840

B8029BD2-A7E7-5D94-92E6-CFE4E7AC3389

Figs 3P , 8W , 9G, M

≡ Fimbriaria A.Juss., Fl. Bras. Merid. (quarto ed.) 3(22): 63. 1833, nom. illeg., non Stackh. (1809), nec Nees ex Steud. (1824).

##### Type species.

*Fimbriariaelegans* A.Juss. [= *Schwanniamediterranea* (Vell.) R.F.Almeida & M.Pell.].

##### Notes.

Despite being the oldest available name for this genus, *Fimbriaria* A.Juss. is illegitimate for being a later homonym to *Fimbriaria* Stackh. (Rhodomelaceae, Rhodophyta). This is unaffected by the posterior rejection of *Fimbriaria* Stackh. against *Odonthalia* Lyngbye. Furthermore, even if this rejection made “*Fimbriaria*” available as a generic name, *Fimbriaria* Nees ex Steud. (Aytoniaceae, Marchantiophyta) still has priority over the Malpighiaceae name. Therefore, *Schwannia* is the earliest available name for this genus.

*Schwannia* comprises 14 currently accepted species (seven threatened species; Suppl. material 1) of shrubs or lianas endemic to rainforests, savannas, and seasonally dry tropical forests of Bolivia, Brazil, and Paraguay, South America (POWO 2024). For an identification key for all species of *Schwannia*, see Sebastiani (2010) and Sebastiani and Mamede (2014), both under *Janusia*.

#### ﻿2.9.12.a. Schwannia christianeae

Taxon classificationPlantaeMalpighialesMalpighiaceae

(W.R.Anderson) R.F.Almeida & M.Pell. comb. nov.

480A1D88-0218-5F81-900A-C4912F3A35D3

urn:lsid:ipni.org:names:77342430-1

≡ Janusiachristianeae W.R.Anderson, Contr. Univ. Michigan Herb. 16: 80. 1987.

#### ﻿2.9.12.b. Schwannia diminuta

Taxon classificationPlantaeMalpighialesMalpighiaceae

(R.Sebast. & Mamede) R.F.Almeida & M.Pell. comb. nov.

6D1EDDBF-16F5-537E-8239-FD5530F9F0A8

urn:lsid:ipni.org:names:77342431-1

≡ Janusiadiminuta R.Sebast. & Mamede, Hoehnea 41(1): 121. 2014.

#### ﻿2.9.12.c. Schwannia hexandra

Taxon classificationPlantaeMalpighialesMalpighiaceae

(Vell.) R.F.Almeida & M.Pell. comb. nov.

60505C3C-1AE2-5E5E-A200-49D8A3150D33

urn:lsid:ipni.org:names: 77342435-1

≡ Banisteriahexandra Vell., Fl. Flum.: 188. 1825.

#### ﻿2.9.12.d. Schwannia mediterranea

Taxon classificationPlantaeMalpighialesMalpighiaceae

(Vell.) R.F.Almeida & M.Pell. comb. nov.

70013DCB-0ED8-5A48-94B8-7621ACB579CB

urn:lsid:ipni.org:names:77342436-1

≡ Banisteriamediterranea Vell., Fl. Flumin.: 191. 1829.

#### ﻿2.9.12.e. Schwannia occhionii

Taxon classificationPlantaeMalpighialesMalpighiaceae

(W.R.Anderson) R.F.Almeida & M.Pell. comb. nov.

EF186D55-9D9D-5C40-ADA2-081477FF7021

urn:lsid:ipni.org:names:77342438-1

≡ Janusiaocchionii W.R.Anderson, Contr. Univ. Michigan Herb. 16: 84. 1987.

#### ﻿2.9.12.f. Schwannia paraensis

Taxon classificationPlantaeMalpighialesMalpighiaceae

(R.Sebast. & Mamede) R.F.Almeida & M.Pell. comb. nov.

0B8886F9-B77F-51FE-830E-2DBBA14BD1F9

urn:lsid:ipni.org:names: 77342437-1

≡ Schwanniaparaensis R.Sebast. & Mamede, Hoehnea 41(1): 123. 2014.

#### ﻿2.9.12.g. Schwannia prancei

Taxon classificationPlantaeMalpighialesMalpighiaceae

(W.R.Anderson) R.F.Almeida & M.Pell. comb. nov.

8ACAB1C3-A029-5E1E-831A-B1F942BFD977

urn:lsid:ipni.org:names:77342439-1

≡ Janusiaprancei W.R.Anderson, Contr. Univ. Michigan Herb. 16: 87. 1987.

#### ﻿2.9.12.h. Schwannia schwannioides

Taxon classificationPlantaeMalpighialesMalpighiaceae

(W.R.Anderson) R.F.Almeida & M.Pell. comb. nov.

1035EF4F-69CD-512B-8E2D-ABED73D6524A

urn:lsid:ipni.org:names:77342440-1

≡ Janusiaschwannioides W.R.Anderson, Contr. Univ. Michigan Herb. 15: 133–135, f. 14. 1982.

#### ﻿2.8.13. Sphedamnocarpus

Taxon classificationPlantaeMalpighialesMalpighiaceae

Planch. ex Benth. & Hook.f., Gen. Pl. 1: 256. 1862.

2A7833B1-DC67-5085-90F5-705E9B1E2DCB

= Tricomariopsis Dubard, Compt. Rend. Hebd. Séances Acad. Sci. 145: 1190. 1907. Type species: Tricomariopsismadagascariensis Dubard (= Sphedamnocarpusdubardii R.Vig. & Humbert ex Arènes).
= Banisterioides Dubard & Dop, Rev. Gén. Bot. 20: 356. 1908. Type species: Banisterioidesmadagascariensis (Baker) Dubard & Dop (= Sphedamnocarpusmultiflorus Nied.).

##### Type species.

*Sphedamnocarpusangolensis* (A.Juss.) Oliv.

##### Notes.

*Sphedamnocarpus* comprises ten currently accepted species (nine threatened species; Suppl. material 1) of shrubs or lianas endemic to savannas of Angola, Madagascar, Malawi, Mozambique, Namibia, South Africa, Swaziland, Zambia, and Zimbabwe, Africa (POWO 2024). For an identification key for all species of *Sphedamnocarpus*, see Arènes (1943b).

#### ﻿2.9.14. Stigmaphyllon

Taxon classificationPlantaeMalpighialesMalpighiaceae

A.Juss., Fl. Bras. Merid. 3: 48. 1833 [1832].

1A98E79D-FAFD-5FF5-8E2B-54F248BF6219

Figs 3C, N , 4A–C, E–G, I–N , 6D, E , 9N, P, Q

= Brachypterys A.Juss. in Deless., Icon. Sel. Pl. 3: 20. 1838. Type species: Brachypterysaustralis A.Juss. (= Stigmaphyllonparalias A.Juss.).
= Ryssopterys Blume ex A.Juss. in Deless., Icon. Sel. Pl. 3: 21. 1838. Type species: Ryssopterystimoriensis (DC.) Blume ex A.Juss. [≡ Stigmaphyllontimoriense (DC.) C.E.Anderson].

##### Type species.

*Stigmaphyllonauriculatum* (Cav.) A.Juss.

##### Notes.

*Stigmaphyllon* comprises 119 currently accepted species (60 threatened species; Suppl. material 1) of shrubs, subshrubs or lianas endemic to rainforests, savannas, and seasonally dry tropical forests of the Americas (from Mexico to Argentina), West Africa (Guinea, Guinea-Bissau, Liberia, Senegal, and Sierra Leone), Southeast Asia (Indonesia, Malaysia, Philippines, Timor-Leste), and Oceania (Australia, Papua New Guinea, Solomon Islands, Vanuatu, and New Caledonia; POWO 2024). For an identification key for all species of *Stigmaphyllon*, see Anderson (1997b, 2011) or Almeida et al. (2020) for Brazil.

## ﻿Discussion

The phylogenetics of Malpighiaceae has been the subject of at least 17 different studies based on plastid and nuclear markers over the last two decades. Eight of these studies focused on the family as a whole, trying to sample its main morphological or phylogenetic groups (Cameron et al. 2001; Davis et al. 2001; Davis and Chase 2004; Davis and Anderson 2010; Davis et al. 2014; Willis et al. 2014; Cai et al. 2016; Almeida et al. 2023a, b, c; Do et al. 2024). Nine of these studies focused on phylogenetic clades (i.e., Tetrapteroid clade ≡ tribe Hiptageae) or specific genera (i.e., *Acridocarpus*, *Amorimia*, *Chlorohiptage*, *Hiptage*, *Lasiocarpus*, *Mionandra*, and *Stigmaphyllon*; Davis 2002; Almeida et al. 2018; 2023a, b, c; Tan et al. 2019; Almeida and van den Berg 2020, 2021, 2022; Cardona-Cruz et al. 2021; Do et al. 2024). All ten phylogenetic clades recognised by us here in the new classification system proposed for Malpighiaceae have consistently been recovered in almost all previous molecular phylogenetic studies (Cameron et al. 2001; Davis et al. 2001; Davis and Chase 2004; Davis and Anderson 2010; Davis et al. 2014; Willis et al. 2014; Almeida et al. 2023a, b, c; Do et al. 2024).

In fact, different lines of evidence, besides DNA, support our new classification system. Pollen grain morphology also recovered different pollen types in Malpighiaceae that characterise both subfamilies and most of the tribes recognized in our study (Lowrie 1982). Tribes Gaudichaudieae, Hiptageae, and Malpighieae were the only ones palynotaxonomically poorly characterised due to the incomplete taxonomic sampling for their currently accepted genera (Lowrie 1982). Secondary metabolites have also been recently evidenced as important characters supporting the classification system proposed here. Mannochio-Russo et al. (2022) studied over 300 samples of all phylogenetic clades of Malpighiaceae by GNPS + molecular networking methods, evidencing the presence of at least 78 different secondary metabolites produced by this family. The authors also evidenced that all subfamilies and tribes recognised here by us are supported by the presence/absence of one to twelve secondary metabolites.

Finally, the previously mentioned non-monophyly of *Rhynchophora* and *Madagasikaria* is a minor issue that will be easily solved by additional taxonomic and genomic sampling in future studies in the family. Additionally, the uncertain placement of *Ectopopterys* within tribe Malpighieae can only lead to its placement in another currently recognised tribe or to the proposition of a new tribe to accommodate this peculiar monospecific genus. Thus, the integrity and phylogenetic confidence of the new classification system proposed here for Malpighiaceae will remain strong, further advancing the taxonomic knowledge of this important family of flowering plants.

## ﻿Conclusions

After over two decades of phylogenetic studies in Malpighiaceae and almost three centuries of taxonomic work, we finally have the proposition of the first classification system with a monophyletic recircumscription of all currently accepted genera and an updated species list for this family. A total of two subfamilies, 12 tribes, 72 genera, and 1,499 species are accepted in this study for Malpighiaceae worldwide as a solid basis for future systematic and taxonomic study in this family. Even though generic circumscriptions are now monophyletic and re-circumscribed, generic relationships within its most species-rich tribes (i.e., Gaudichaudieae, Hiptageae, Hiraeeae, and Malpighieae) still need further phylogenetic/omic studies for better statistical support and proposition of a subtribal system. Taxonomic revisions are urged for most genera of this family, especially the most species-rich ones [i.e., *Heteropterys* (166 spp.), *Byrsonima* (164), *Bunchosia* (93), *Hiraea* (81), *Tetrapterys* (56), *Mascagnia* (48), *Hiptage* (47), *Acridocarpus* (36), *Glicophyllum* (28), *Aspicarpa* (27), *Aspidopterys* (24), *Triaspis* (19), *Niedenzuella* (18), *Schwannia* (14), *Bronwenia* (13), *Spachea* (12), *Sphedamnocarpus* (10), *Psychopterys* (9), *Carolus* (8), *Mamedea* (7), *Jubelina* (6), *Christianella* (5), *Adelphia* (4), *Excentradenia* (4), *Malpighiodes* (4), *Ptilochaeta* (3), *Brachylophon* (2), *Calcicola* (2), *Caucanthus* (2), *Echinopterys* (2), *Heladena* (2), and *Janusia* (2)]. Finally, special attention is urgently needed to the taxonomy of the long-neglected and highly threatened African and Asian Malpighiaceae.

## ﻿Additional information

### Author ORCIDs

Rafael F. de Almeida https://orcid.org/0000-0002-9562-9287

Isa L. de Morais https://orcid.org/0000-0001-8748-9723

Thais Alves-Silva https://orcid.org/0009-0001-0760-6019

Higor Antonio-Domingues https://orcid.org/0000-0001-9405-1930

Marco O. O. Pellegrini https://orcid.org/0000-0002-8783-1362

### Data availability

All of the data that support the findings of this study are available in the main text or Supplementary Information.

## Supplementary materials

Supplementary material 1 Checklist of accepted species names of Malpighiaceae Data type xlsx https://binary.pensoft.net/file/1053715This dataset is made available under the Open Database License (http://opendatacommons.org/licenses/odbl/1.0/). The Open Database License (ODbL) is a license agreement intended to allow users to freely share, modify, and use this Dataset while maintaining this same freedom for others, provided that the original source and author(s) are credited.Rafael F. de Almeida, Isa L. de Morais, Thais Alves-Silva, Higor Antonio-Domingues, Marco O. O. Pellegrini

Supplementary material 2 Morphological matrix including 31 scored and coded characters used in the phylogenetic optimization Data type xlsx https://binary.pensoft.net/file/1053716This dataset is made available under the Open Database License (http://opendatacommons.org/licenses/odbl/1.0/). The Open Database License (ODbL) is a license agreement intended to allow users to freely share, modify, and use this Dataset while maintaining this same freedom for others, provided that the original source and author(s) are credited.Rafael F. de Almeida, Isa L. de Morais, Thais Alves-Silva, Higor Antonio-Domingues, Marco O. O. Pellegrini
